# Mechanistic Characterization of Biologically Inspired Oral Neutrophil Isolation for AI-Assisted Oral Inflammatory Load Assessment

**DOI:** 10.3390/biomimetics11090673

**Published:** 2026-09-18

**Authors:** Fatemeh Soheili, Mahdi S. M. H. Daneshvar, Navid Mohaghegh, John V. L. Nguyen, Abbas Panahi, Mahdi Amrollahi Biouki, Yasaman Tahernezhad, Saghi Forouhi, Chunxiang Sun, Michael Glogauer, Ebrahim Ghafar-Zadeh

**Affiliations:** 1 Biologically Inspired Sensors and Actuators Laboratory (BioSA), Department of Electrical Engineering and Computer Science, Lassonde School of Engineering, York University, 4700 Keele Street, Toronto, ON M3J 1P3, Canadasaghi.forouhi@liu.se (S.F.); 2 Department of Biology, York University, 4700 Keele Street, Toronto, ON M3J 1P3, Canada; 3 Department of Biomedical Engineering, McGill University, 3775 University Street, Montreal, QC H3A 2B4, Canada; 4 Division of Electronics and Computer Engineering (ELDA), Department of Electrical Engineering (ISY), Linköping University, 581 83 Linköping, Sweden; 5 Faculty of Dentistry, University of Toronto, Toronto, ON M5G 1G6, Canada

**Keywords:** biologically inspired sensors and actuators, periodontal diseases, oral neutrophil, artificial intelligence, adhesion-based cellular isolation from saliva

## Abstract

Non-invasive quantitative assessment of oral inflammatory burden could complement conventional periodontal evaluation; however, current clinical methods primarily characterize disease after structural and tissue changes have occurred. In our previously reported DePerio framework, we demonstrated the feasibility of enriching oral polymorphonuclear neutrophils (oPMNs) from saliva through their differential adhesion to a hydrophilic cornstarch (CS)-coated surface, followed by brightfield imaging and AI-assisted quantification. Building on this framework, here we present BioSA, with an emphasis on the mechanistic and quantitative characterization of this biologically inspired adhesion-based oPMN isolation process, analogous to leukocyte adhesion behavior in blood capillaries. We systematically investigate the contributions of surface physicochemical properties, oPMN surface characteristics, and physical forces governing preferential oPMN retention and epithelial-cell removal. The optimized adhesion-based isolation reduced epithelial-cell contamination by 91% while preserving >98% of oPMNs, outperforming the evaluated filtration- and poly-L-lysine (PLL)-based approaches. Following isolation, brightfield images were analyzed using an AI-assisted deep-learning model for automated oPMN quantification. Across 30 independent test days, BioSA measurements were strongly associated with those obtained using the reference HEMO method (R^2^ ≈ 0.99; MAE ≈ 2.06 × 10^5^ cells/10 mL), while method agreement and systematic differences were further evaluated using Bland–Altman analysis. The deep-learning detector achieved 98% sensitivity, 97% precision, and an F1 score of 0.97 on a dataset containing 4617 manually annotated oPMNs. Using data-driven oPMN concentration ranges informed by prior literature, we further explored five oral inflammatory load (OIL) strata. These strata are hypothesis-generating and should not be interpreted as validated diagnostic stages or as replacements for the 2017 periodontitis staging and grading framework. Operating with standard brightfield microscopy and a cloud-based AI interface, BioSA reduces analysis time from 15 to 20 min to less than 2 min per sample. Collectively, this study extends the DePerio framework by providing mechanistic insight into biologically inspired adhesion-based oPMN isolation and demonstrates its potential for rapid, quantitative, and exploratory assessment of oral inflammatory burden.

## 1. Introduction

Periodontal disease (PD) is a chronic, progressive inflammatory disorder that compromises the integrity of gingival tissue, alveolar bone, and the connective framework supporting the teeth [1,2]. Clinically, its advancement can lead to tooth loss through the destruction of the periodontal ligament and surrounding structures [3]. Its prevalence makes it a pressing public health issue: over 47% of adults aged 30 and older are affected, with rates escalating to 70% in those aged 65 and above [2]. The onset of PD is gradual yet insidious. Early PD remains difficult to diagnose because conventional clinical measures—probing depth, radiography, and clinical attachment loss—primarily detect pathology after irreversible tissue destruction.

As shown in Figure S1, in the Supplementary Information (SI), a thin pellicle layer forms within minutes of cleaning [4], eventually evolving into plaque and hardening into calculus. This microbial biofilm initiates gingivitis within days to weeks [5,6]. Without timely intervention, inflammation deepens, leading to periodontal pocket formation, clinical attachment loss (CAL) [7,8], and progressive bone resorption, culminating in tooth loss [9]. More than a localized condition, PD has systemic implications. Chronic inflammation in the periodontal tissue facilitates the translocation of bacteria and pro-inflammatory mediators into the bloodstream [10,11]. As depicted in Figure S1B, this systemic inflammatory burden has been implicated in a wide range of comorbidities, including cardiovascular diseases [12], diabetes mellitus [13,14,15,16,17], rheumatoid arthritis [18], and adverse pregnancy outcomes like preterm birth and low birth weight [19,20,21]. Emerging evidence also links PD to oral cancer [22,23,24,25], Alzheimer’s disease [26,27], and osteoporosis [18,22,28,29,30,31,32]. The growing recognition of these associations emphasizes the critical need for effective, early-stage diagnostic and prevention strategies, particularly those that move beyond clinical manifestations to detect underlying inflammatory activity before irreversible damage occurs.

Early detection of PD remains a challenge with conventional diagnostic tools. Methods such as visual examination, CAL measurement [33], bleeding on probing (BOP) [34], and radiographic bone loss (RBL) [35] (Figure S1C) are standard in dental practice but detect PD only after irreversible structural changes have occurred. Even advanced imaging modalities like cone beam computed tomography (CBCT) [36]—though precise—remain limited to identifying post-damage pathology. To address this gap, recent advances have turned toward molecular biosensing (Figure S1D) for early-stage detection. Joe et al. developed a portable electrochemical aptasensor targeting odontogenic ameloblast-associated protein (ODAM), achieving a detection limit of 1 nM through aptamer duos and chronoamperometry [37]. Building on this, Nguyen et al. implemented a sandwich-type aptamer configuration (OD64/OD35) that enabled patient risk stratification using clinical parameters such as BOP and probing depth [38]. Other studies have explored molecular biomarkers, including matrix metalloproteinase-8 (MMP-8) [39], oxidative stress markers like hydrogen peroxide and hypochlorous acid [40], and inflammatory proteins such as C-reactive protein (CRP) [41,42] and interleukin-1β (IL-1β) [43,44] in saliva or Gingival Crevicular Fluid (GCF) [45]. While promising, these molecular platforms are constrained by limitations such as biological variability, low specificity, and a tendency to yield binary rather than graded outputs. To overcome these barriers, we propose a cellular biomarker-based biosensing strategy for the early detection and dynamic severity scoring of PD. By targeting the functional and adhesive characteristics of immune cells—specifically oral neutrophils—our approach provides a biologically relevant, quantifiable, and scalable alternative to conventional molecular diagnostics.

oPMNs serve as frontline immune defenders in the oral cavity (Figure S1E), rapidly migrating to infection sites to eliminate pathogens via phagocytosis and antimicrobial release [46,47]. Under persistent microbial stress—especially in dysbiotic environments—this defense becomes chronic, fueling a self-sustaining inflammatory cycle and amplifying tissue damage [48]. Due to their direct involvement in oral immune responses, oPMNs have emerged as promising biomarkers for OIL and PD severity [18,22,29,49,50,51]. Their salivary concentrations correlate with disease stages from gingivitis to severe periodontitis [52]. However, systemic influences—such as chemotherapy, autoimmune treatments, infections, or chronic diseases like diabetes—can significantly alter oPMN levels [53,54,55,56,57,58,59,60], complicating interpretation [61].

Accurate oPMN quantification remains a technical challenge. Filtration techniques isolate oPMNs by size (~10–12 µm [62] vs. >20 µm for epithelial cells [49,63]), but the process is labor-intensive, prone to debris contamination, and not suited for high-throughput use [49,64]. Alternatively, fluorescent-based manual hemocytometry (HEMO) using dyes like Acridine Orange allows direct oPMN visualization [25,65,66,67,68,69,70,71,72], but requires costly microscopy and skilled personnel. Papanicolaou (Pap) staining has recently been proposed for cellular analysis in saliva [73], yet faces similar barriers for routine or point-of-care deployment. Despite these limitations, oPMNs remain a biologically relevant, non-invasive indicator of PD activity. When interpreted within the broader clinical context, their quantification offers meaningful insights into inflammatory status and disease severity. Although oPMNs are recognized indicators of OIL, current oPMN enumeration methods rely on fluorescence microscopy, flow cytometry, or staining procedures that require specialized equipment and laboratory expertise, limiting their routine use in dental clinics or point-of-care settings. To address this limitation, our previously reported DePerio framework established the feasibility of differential adhesion-based oPMN isolation from saliva using a hydrophilic CS-coated surface, followed by brightfield imaging and AI-assisted quantification. Building on this framework, the present work focuses on the mechanistic and quantitative characterization of the biologically inspired adhesion-based isolation process and its application to OIL assessment.

**Figure 1:**
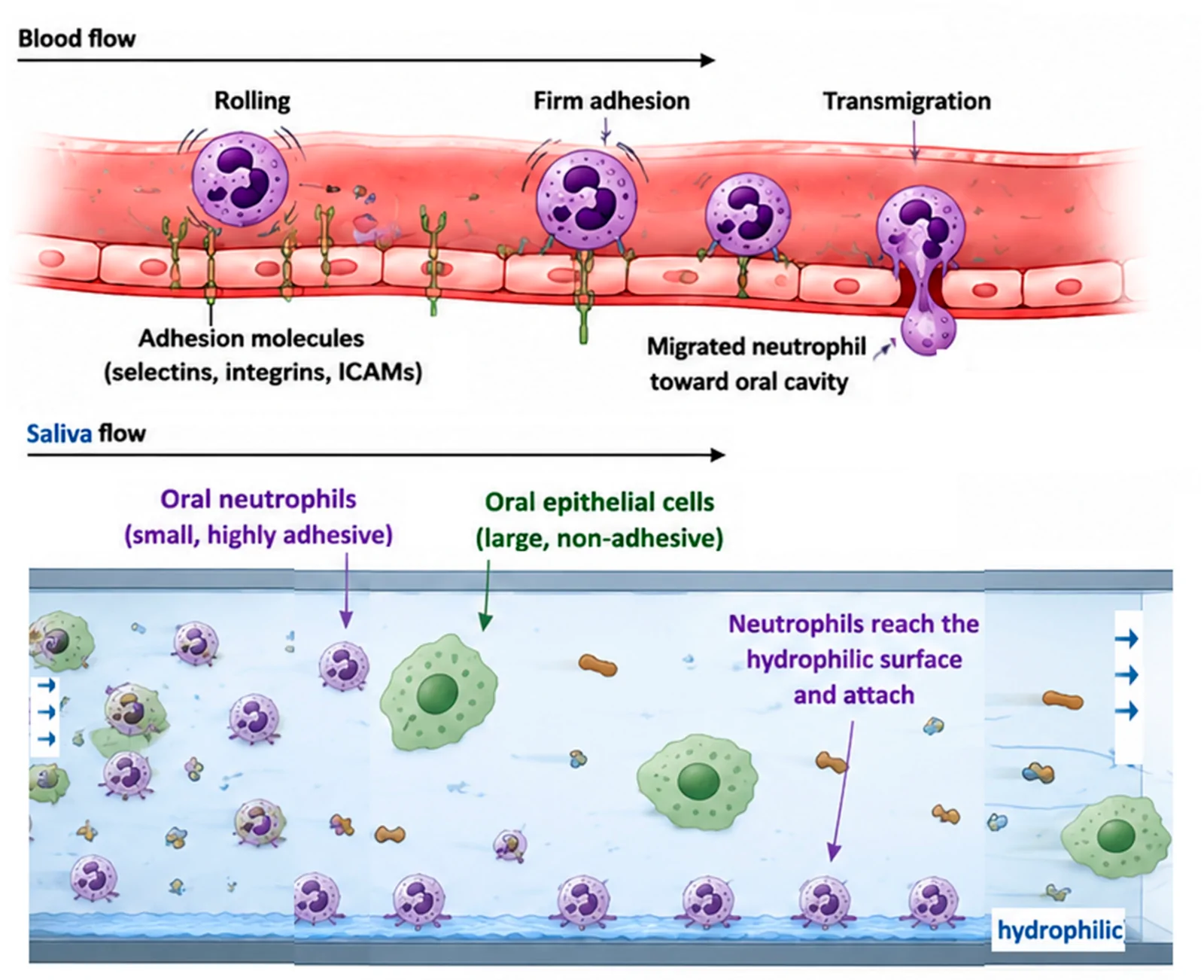
Biologically inspired adhesion mechanism underlying the BioSA platform. Top: Physiological recruitment of neutrophils from blood vessels into the oral cavity through rolling, firm adhesion, and transmigration mediated by adhesion molecules (selectins, integrins, and ICAMs). Bottom: BioSA mimics this natural adhesion process by exploiting the intrinsic adhesive properties of oral polymorphonuclear neutrophils (oPMNs) on a hydrophilic cornstarch-coated surface. Small, highly adhesive oPMNs selectively attach to the hydrophilic substrate, whereas larger oral epithelial cells and debris remain non-adherent and are removed by fluid flow, enabling label-free enrichment of oPMNs for rapid brightfield imaging and AI-based quantification.

oPMNs originate from WBCs that transmigrate from blood vessels into the gingival tissue and then into the oral cavity [74]. This migration is facilitated by adhesion molecules such as CD18/CD11b. In addition to immune cells, saliva also contains exfoliated epithelial cells, contributing to sample complexity [75,76]. As illustrated in Figure 1, BioSA emulates the natural adhesion behavior of neutrophils during vascular recruitment. Similar to the rolling, firm adhesion, and transmigration of neutrophils mediated by endothelial adhesion molecules, oPMNs preferentially adhere to the hydrophilic BioSA substrate, while larger epithelial cells and debris remain non-adherent and are removed during washing. This biologically inspired mechanism enables label-free enrichment of oPMNs for subsequent AI-assisted brightfield analysis. Unlike circulating neutrophils, oPMNs exhibit enhanced surface activity due to upregulated adhesion-related CD markers, including CD11a, CD11b, CD18, CD62L, CD66a, and CD66b [77,78,79]. These markers are rich in hydrophilic residues, such as hydroxyl and sialic acids, which promote strong adhesion to surfaces modified with oxygen plasma or hydrophilic materials like CS [80,81,82]. Building on the adhesion-based isolation principle previously demonstrated in DePerio, the present study further investigates the biological and physicochemical basis of this process, including the contribution of oPMN surface properties and the physical forces governing cell retention and removal. [83]. To validate this method, manual cell counts were conducted, revealing reliable separation. To streamline this process, we developed a brightfield AI-based detection pipeline, offering a cost-effective alternative to fluorescence microscopy [84]. This system distinguishes oPMNs even in debris-rich saliva samples, advancing the potential for affordable, automated, and point-of-care monitoring of PD.

Deep learning has transformed cell analysis, particularly through convolutional neural networks (CNNs), which have achieved high accuracy in blood cell detection, classification, and segmentation [85,86,87,88,89]. Models like AlexNet [90], YOLO [91], ResNet50 [92], and InceptionV3 [92] have been applied to blood smears, while U-Net variants—including U-Net++ [93,94,95] and deformable U-Net [96]—have advanced segmentation tasks. Faster R-CNN has also proven effective for red blood cell detection [72]. However, these methods remain underutilized for analyzing saliva-derived immune cells. Building on the AI-assisted oPMN quantification demonstrated in the DePerio framework, BioSA employs a YOLO-based AI pipeline for detecting and quantifying oral neutrophils (oPMNs) in brightfield images [91,97]. YOLO’s single-stage architecture supports real-time, multiscale detection via modules like Feature Pyramid Networks (FPN) and Path Aggregation Networks (PANet) [98,99]—enhancing accuracy in identifying small, overlapping cells typical of saliva samples. Details on the performance of the custom-designed DNN model, along with statistical analyses, are provided in the Supplementary Materials document. By deploying the deep learning model on a cloud platform or a private server, remote access can be enabled for dental or periodontal clinics, integrating them into the Internet of Medical Things (IoMT). This represents a significant step toward the global adoption of this technology (see Figure S1F in the SI). In this paper, we also address this challenge by developing an AI platform that includes a private server along with associated front-end and back-end software components.

In this work, we build upon the previously demonstrated adhesion-based strategy by systematically investigating the biological and physical mechanisms underlying selective oPMN isolation. The approach draws directly from the natural adhesion behavior of oPMNs in the oral cavity, where these cells exhibit strong surface affinity due to their hydrophilic membranes and abundant surface receptors such as CD11b. In contrast, epithelial cells show lower intrinsic hydrophilicity and detach more readily under mild fluid motion. By engineering a hydrophilic CS-based coating that enhances this inherent adhesion differential, our method mimics the biological mechanism through which oPMNs naturally interact with mucosal surfaces. The present study provides further mechanistic characterization of this biologically inspired capture process, which forms the foundation of the AI-assisted quantification pipeline.

This study builds directly on the foundational clinical works that established oPMNs as a reliable indicator of OIL. While earlier methods relied on fluorescence microscopy, flow cytometry, and antibody-based labeling for oPMN enumeration, our previously reported DePerio study translated these biological insights into an adhesion-based engineering framework for oPMN isolation and AI-assisted quantification. The present study builds upon that framework by providing a more systematic mechanistic and quantitative investigation of the factors governing differential oPMN adhesion and retention (See Table S1 in the SI). By combining this mechanistically characterized bio-inspired, low-cost adhesion-based isolation method with an AI-powered quantification pipeline and an accessible web-based interface, the BioSA platform provides a practical pathway toward scalable OIL assessment. This translational direction aims to support future deployment in dental clinics, community screening programs, and mobile periodontal care.

Central to this work is the mechanistic characterization of a bio-inspired, adhesion-based isolation strategy that leverages the intrinsic hydrophilicity and surface-receptor profile of oPMNs to enable selective retention on a hydrophilic substrate, forming the foundation of the BioSA workflow.

## 2. Materials and Methods

This method consists of two parts: a biologically inspired isolation technique and AI-powered quantification, as described in this section and elaborated in Figure 1. Building on the adhesion-based isolation and AI-assisted quantification framework previously demonstrated in DePerio, the present study further investigates the biological and physicochemical mechanisms underlying the selective adhesion and retention of oPMNs. All methods were performed following the relevant guidelines and regulations as outlined in the Nature Research editorial policies.

All clinical saliva samples were provided by the Matrix Dynamics Group under approved ethics protocol Certificate No. 41730, issued by the Faculty of Dentistry, University of Toronto. Healthy control samples collected at York University were obtained under ethics approval 2025-143. In accordance with these protocols, saliva samples were collected from an equal number of male and female volunteers aged 25–60 years, and all samples were anonymized and randomly selected prior to analysis.

### 2.1. Biologically Inspired Isolation Method

**Figure 2:**
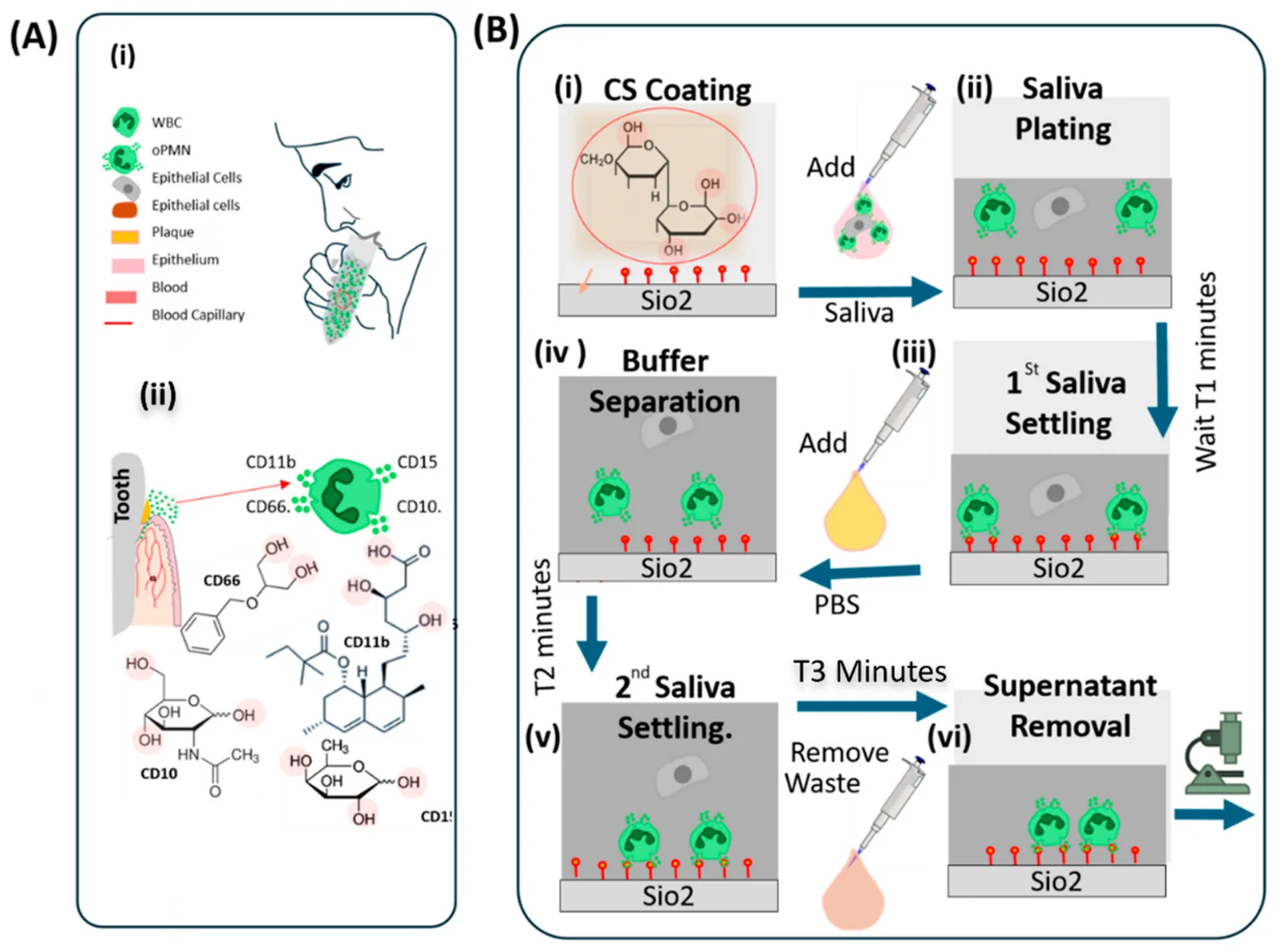
Illustration of the proposed oPMN isolation workflow: (**A**) Overview of the method, including (**i**) the biologically inspired adhesion-based capture strategy, (**ii**) the role of CD markers in oPMN adhesion, (**B**) Experimental procedure: (**i**) surface treatment of the Petri dish using corn starch, (**ii**) saliva deposition, (**iii**) after the sample settling period (T1) or pre-WB waiting period, (**iv**) addition of WB, (**v**) post-WB phase (T2), and (**vi**) removal of the supernatant followed by microscopy imaging.

The adhesive behavior of WBCs within capillaries [74] inspired the hypothesis that similar properties may be exhibited by oPMNs within the oral cavity. Preliminary observations of plated saliva samples indicate that oPMNs adhere to surfaces more rapidly and firmly than epithelial cells (Supplementary Materials, Video). Notably, during pipette suction, oPMNs exhibit stronger surface affinity, often remaining attached, while epithelial cells detach more readily. This differential adhesion—marked by the strong binding of oPMNs to hydrophilically coated Petri dishes (HCPDs) treated with CS, in contrast to the weaker attachment of epithelial cells—forms the basis of the Biologically Inspired Isolation Method. Both oPMNs and epithelial cells, the largest constituents in saliva rinse samples, are readily visualized under brightfield microscopy at appropriate magnifications. After removal of the epithelial cells, the HCPD surface remains primarily populated with oPMNs and minimal residual debris, allowing for identification through a deep learning-based AI model. The strong adherence of oPMNs is influenced by their expression of hydrophilic CD markers such as CD11, CD16, and CD64 [83,100] (Figure 2A(ii)), which are known to promote binding to hydrophilic substrates [101,102,103,104,105]. Although antibody-based targeting of these markers [106,107] can enable selective isolation, we hypothesized that their intrinsic hydrophilic interaction could be leveraged as a low-complexity, cost-effective alternative. In this approach, a saliva rinse sample (Figure 2A(ii)) is simply deposited onto a hydrophilic coating material (HCM) surface (Figure 2B(i)). The selective adhesion of oPMNs occurs naturally, without requiring biochemical surface modifications. To enhance this effect, surface hydrophilicity can be increased using oxygen plasma treatment or hydrophilic coatings such as CS. Additionally, differences in cell size and morphology—oPMNs (~5 µm diameter [63]) versus epithelial cells (>15 µm, sheet-like)—as well as gravitational and drag forces, further contribute to distinct sedimentation and suction dynamics [108,109]. Following deposition of the saliva rinse sample onto the HCM (Figure 2B(ii–vi)), the suspension was allowed to settle for 10–15 min (T1). During this period, oPMNs rapidly adhered to the CS-coated glass through hydrophilic interactions between the glucose hydroxyl groups of the CS matrix and exposed polar surface markers on oPMNs (e.g., CD11b, CD18, CD16, CD62L, CD66a/b). Exfoliated epithelial cells, which are significantly larger (15–80 µm), sheet-like, and exhibit relatively low hydrophilicity, remained suspended or settled loosely on the surface without stable adhesion. After the settling step, 1 mL of PBS was gently added along the wall of the dish (T2). The large size and flat morphology of epithelial cells resulted in greater drag force during PBS flow, causing them to detach easily and remain in the supernatant, whereas adherent oPMNs remained stably attached to the CS surface. The supernatant containing epithelial cells was then removed by gentle pipette aspiration (T3), effectively eliminating epithelial contaminants while preserving oPMNs on the coating. This workflow was validated across multiple donors, and optimization data (Tables S6 and S7) confirmed that epithelial cells were consistently removed while oPMNs remained firmly anchored to the CS-coated substrate.

In our previously reported DePerio study [110], we established the feasibility of enriching oPMNs from saliva through their differential adhesion to a hydrophilic corn-starch-coated surface, followed by bright-field imaging and AI-assisted detection and quantification. DePerio further evaluated oPMN concentration as a measure of Oral Inflammatory Load (OIL) and compared the resulting measurements with the reference HEMO method. While DePerio established the feasibility of this overall workflow, the biological, physicochemical, and physical mechanisms governing preferential oPMN retention and epithelial-cell removal were not comprehensively characterized. Building on this framework, the present BioSA study focuses on the mechanistic and quantitative characterization of the adhesion-based separation process by investigating the effects of surface properties, oPMN surface characteristics, hydrophilicity, cell morphology, sedimentation, and fluid-induced forces on differential cell retention and removal.

### 2.2. Reagents and Protocols

#### 2.2.1. Sample Collection

Healthy volunteers aged 18–60 (both male and female) were recruited following approval from the York University Ethics Committee. Participants were asked to refrain from eating or drinking (except water) for two hours before sample collection. Saliva was collected by rinsing the mouth with 10 mL of tap water for 15 s and discarding the fluid. After two minutes, the mouth was rinsed with 10 mL of 0.9% NaCl (normal saline) for 30 s, and this sample was collected. To increase sample volume, this rinse was repeated until a total of 40 mL was obtained. All samples were transferred into sterile conical centrifuge tubes. On average, 10 mL of collected sample contained 5 × 10^5^ to 7 × 10^5^ oPMNs in healthy individuals [111]. Additionally, Clinical Samples were obtained from the Matrix Dynamic Group Laboratory, collected with University of Toronto ethical approval. BioSA isolation and counting were conducted, and all measurement data and detailed statistical analyses are provided in Supplementary Materials, section “Statistical Analysis” (Tables S6–S20).

#### 2.2.2. Rinse Buffer Preparation

A 0.9% NaCl solution was prepared by dissolving 9 g of sodium chloride (Sigma-Aldrich, Toronto, ON, Canada) in 1 L of distilled water. Sodium chloride was measured using a precision balance (Accuris Inc, Toronto, ON, Canada), and the solution was stirred thoroughly until fully dissolved. The final solution was stored in a sterile container until used [112].

#### 2.2.3. HCM Surface Coating

In this study, all oPMN isolation procedures were performed using a HCM composed of a CS-based polysaccharide layer applied to sterile glass Petri dishes. Upon partial drying, the CS layer forms a gel-like network in which glucose hydroxyl (–OH) groups hydrogen-bond to silanol groups on the glass surface, creating a stable hydrophilic substrate. During oPMN capture, exposed polar residues on CD11b can form complementary hydrogen bonds with the CS matrix, promoting selective adhesion. This CS coating was selected after preliminary optimization because it provides stable hydrophilicity for at least 48 h, remains intact under wet conditions during the isolation workflow, and offers a simple, reproducible, and clinically practical method for adhesion-based oPMN capture. CS (Sigma-Aldrich Canada) was used as an HCM on sterile glass Petri dishes following the protocol in [113]. Dry starch (10 mg) was wet with 20 mL of ethanol, then mixed with 5 mL of 90% DMSO. The mixture was heated in a boiling water bath for 1 h with magnetic stirring, followed by 24 h of mild stirring at room temperature (155 RPM). Then, 600 µL of the solution was spread across the Petri dish surface and dried for one hour at 60 °C to form a hydrophilic layer. After drying, the coated plates were stored at room temperature (20 to 25 °C) under ambient laboratory conditions (40–60% relative humidity). No rehydration step was performed prior to use. Plates were analyzed within 24 h of preparation, typically within a few hours. Over this period, no qualitative change in wetting behavior or handling characteristics was observed. More details can be found in the Supplementary Materials Section “Further CS characterization results”.

#### 2.2.4. Oxygen Plasma Treatment

Prior to plasma activation using the Zepto LF PC (Diener Electronic, Ebhausen, Baden-Württemberg, Germany), ensure the machine and Petri dishes are dust-free by cleaning with 70% ethanol and air drying in a clean environment. Immediately place the cleaned dish inside the plasma chamber. Set the machine to 70% power, close the lid, and activate the vacuum pump. Once pressure drops to 0–0.1 mbar, introduce oxygen until stabilized at 0.2 mbar. Initiate the plasma treatment, verifying activation by the presence of violet light, and maintain exposure for 3 min and 30 s. Upon completion, switch off the generator and pump, allow pressure to return to ~1 bar, then open the ventilation. Seal the Petri dish promptly and store it in a sterile, airtight condition until the saliva solution is added.

#### 2.2.5. Microscopy and Image Analysis

Brightfield images were captured using a Fisher brand inverted microscope (Thermo Fisher Scientific, Toronto, ON, Canada) with a 20× objective and a Moticam 2 camera. Each image (Si = 0.36 mm^2^) was analyzed using Motic Images Plus 3.0. Given a Petri dish surface area (Sp = 13,952 mm^2^) and image field of view (FOV) Si = 0.64 mm^2^, the scaling factor k = Sp/Si ≈ 2.18 × 10^4^. By sampling m = 10 fields, the average cell count N per image was multiplied by k to estimate total oPMNs in each 10 mL sample [114]. These calculations were integrated into our AI-based counting system.

#### 2.2.6. AI-Based oPMN Quantification

**Figure 3:**
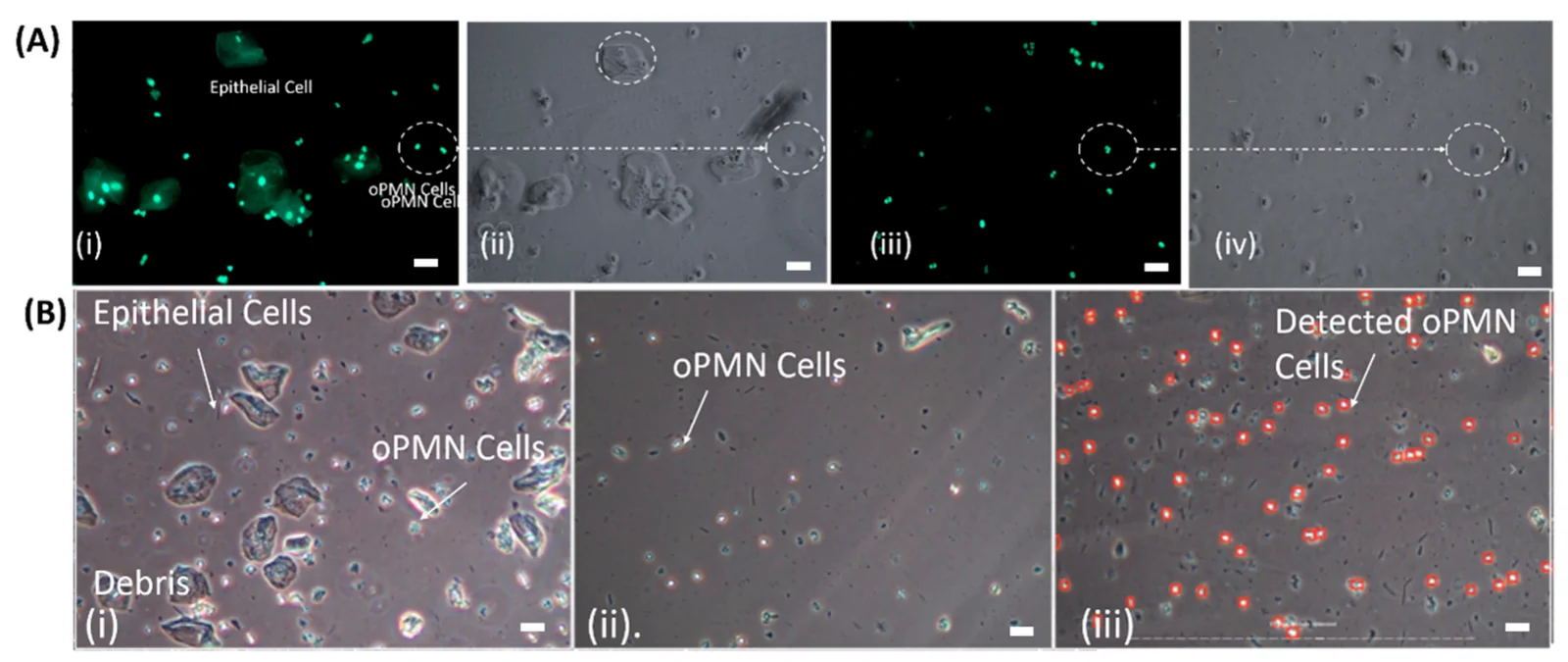
Brightfield and fluorescence microscopy images, along with AI-based oPMN detection results. (**A**) Fluorescence images (**i**,**iii**) and their corresponding brightfield microscopy images (**ii**,**iv**). (**B**) Images captured (**i**) before and (**ii**) after the BioSA isolation process (scale bar: 20 µm), and (**iii**) the result of oPMN detection using the trained AI model. Scale bar: 10 µm.

A web-based application was developed to host the BioSA AI detection model. Users can upload up to 30 images at a time, and the system returns per-image oPMN counts and an overall OIL score. The coefficient k, calculated as described in the Supplementary Materials, section “Categorization: PD prediction”, is user-configurable to reflect individual imaging setups. Snapshots of the interface are shown in Figure 3.

#### 2.2.7. Standard HEMO Counting Method

From each 10 mL saliva sample, 100 µL was mixed with 100 µL of 10% formaldehyde, then centrifuged at 3400 rpm for 5 min. After removing 90 µL of the supernatant, 10 µL of Acridine Orange was added and incubated for 15 min. A 10 µL aliquot was placed on a hemocytometer (BLAUBRAND Neubauer) and observed using a fluorescent microscope (Olympus BX50, 20× objective). Cell counts from four squares were averaged and adjusted using standard calculations to obtain oPMNs per 10 mL [54,64]. The HEMO method served as a control.

#### 2.2.8. Phosphate-Buffered Saline (PBS) Washing Buffer (WB)

PBS (Sigma-Aldrich) was used as a WB to stabilize pH and maintain osmolarity and was also used for mouth rinsing and sample collection, as well as for the separation of non-OPMNs from the CS surface during isolation processing. Its physiological buffering capacity and ionic composition minimize cellular stress and reduce nonspecific interactions during washing steps [115,116].

#### 2.2.9. Calcium Chloride Treatment

Calcium chloride (CaCl_2_; Sigma, Canada) was dissolved in normal saline at concentrations of 0, 0.25, 0.5, and 1 M/mL to generate varying levels of Ca^2+^. These solutions were applied to evaluate the effect of extracellular calcium on the adhesion of oPMNs to the substrate surface.

#### 2.2.10. Hydrophilicity Computational Analysis

We obtained amino acid sequences of various CD markers from the UniProt database [117], a widely used resource for protein sequence and functional information. By querying markers such as CD11b, CD14, and CD16 [118] with their UniProt IDs, we accessed full sequences (in plain text or FASTA format) under the “Sequence” section. These sequences were used for computational analysis of hydropathy profiles using the Kyte–Doolittle scale [117] (see Table S4 in the SI). For instance, the CD11b sequence (UniProt ID: P11215; see Table S5 in the SI) starts as: ALRVLLLTALTLCHGFNLDTENAMTFQENARGFGQSVVQLQGSRVVVGA PQEIVAANQRGSLYQCDYSTGSCEPIRLQVPVEAVNMSLGLSLAATTSPPQ. Hydrophilicity indices were calculated by averaging Kyte–Doolittle values over a sliding window, revealing hydrophilic/hydrophobic regions. We computed each amino acid’s frequency and multiplied it by its hydropathy value, with the sum yielding the overall hydropathy score. In parallel, we compiled the expression frequency of CD markers on oPMNs from the literature, primarily obtained via flow cytometry [119,120,121,122,123]. To estimate cellular hydrophilicity, we multiplied each marker’s expression frequency by its hydropathy index and summed the results for a composite score. This score was then contextualized based on cell size (e.g., ~5 µm for oral neutrophils), reflecting the total surface-exposed hydrophilic potential.

#### 2.2.11. Statistical Analysis

All statistical analyses were performed using R (version 4.3.2). To derive exploratory data-driven oral inflammatory load (OIL) strata from oPMN measurements, we applied k-means clustering with k = 5. The number of clusters was selected to provide a descriptive five-level inflammatory gradient that could be compared with the periodontal health-to-disease spectrum, rather than to establish diagnostic disease-stage cutoffs. This approach was biologically motivated by prior evidence showing that oral neutrophil counts increase with periodontal disease severity and reflect oral inflammatory [21].

Normality of group-wise distributions was assessed using the Shapiro–Wilk test (stats package), and homogeneity of variances was evaluated using Levene’s test (car package, version 3.0-12) [124,125]. When variance homogeneity was violated, comparisons were performed using Welch’s *t*-test (stats package). When both normality and equal variance assumptions were met, analysis of variance (ANOVA) was applied (car package) [124,125,126]. For non-parametric comparisons, the Mann–Whitney U test (coin package, version 1.4-2) [127] was used. A *p*-value of less than 0.05 was considered statistically significant.

Given the limited number of clinical samples and the small size of some PD subgroups, all statistical comparisons and derived thresholds should be interpreted cautiously. In particular, clustering results and downstream disease-stage differentiation are intended to provide exploratory, proof-of-concept insights rather than definitive diagnostic cutoffs. Validation of these statistical groupings and thresholds in larger, independent cohorts is required before clinical generalization.

### 2.3. Proposed oPMN Quantification Method

#### 2.3.1. oPMN Isolation

Following a standardized mouth rinse with 10 mL of normal saline—aligned with established periodontal sampling protocols [64,72]—saliva samples from human participants were plated onto HCPDs treated with CS, as shown in Figure 2B(i–ii). A settling period (T1) allowed oPMNs to predominantly and strongly adhere to the surface (Figure 2B(iii)), while epithelial cells displayed weaker and delayed attachment. Subsequent addition of phosphate-buffered saline (PBS) as a wash effectively removed loosely attached epithelial cells from the surface (Figure 2B(iv)), while oPMNs remained anchored due to their strong hydrophilic interactions—mediated by surface-expressed CD markers. After a brief second interval (T2) (Figure 2B(v)), the supernatant was gently pipetted out (Figure 2B(vi)), removing suspended epithelial cells and leaving a purified oPMN population on the HCPD surface. This process is visually demonstrated in the Supplementary Materials Video, which clearly shows oPMNs remaining adherent while epithelial cells are rapidly separated during suction. After discarding the supernatant, cell enumeration was performed by capturing ten brightfield microscopy images across the dish. While manual quantification proved time-consuming and subject to variability, automated cell detection and counting using a deep learning-based AI model significantly improved speed, accuracy, and reproducibility. BioSA can be streamlined by integrating controllers and mini-pumps instead of a pipette to leverage advantages for dental clinic applications. Herein, critical parameters—including T1, T2, WB volume, surface hydrophilicity, and the number of microscopy images—were systematically optimized through iterative testing. These observations directly guided the design of our adhesion-based isolation protocol by demonstrating that oPMNs exhibit stronger hydrophilic surface affinity than epithelial cells, enabling selective capture on the hydrophilic coating material.

The mechanism of selective removal of epithelial cells and selective enrichment of oPMNs on the hydrophilic surface occurs due to the interplay of differential adhesion strength, cell size, and shear forces during washing. oPMNs (5–10 µm) display rapid and firm adhesion to CS-coated glass due to high expression of hydrophilic adhesion markers such as CD11b, CD18, CD16, and CD66a/b. In contrast, exfoliated epithelial cells (>15 µm, sheet-like morphology) exhibit weak and delayed attachment to hydrophilic substrates.

During the initial settling period (T1), oPMNs adhere strongly within 10–15 min, whereas epithelial cells sediment later and remain only loosely attached. Upon addition of WB, the larger size and irregular morphology of epithelial cells result in higher hydrodynamic drag, causing them to detach and enter the supernatant. oPMNs remain firmly bound to the substrate and therefore are not displaced by low-shear PBS flow. The detached epithelial cells suspended in the supernatant are then removed by gentle pipette aspiration in step (Figure 2B(vi)). Optimization data (Tables S6–S9) confirm that epithelial cell counts are reduced to near zero after washing, while oPMN levels remain unchanged.

Our method was repeated under varied conditions, and oPMN counts were recorded to determine the optimal protocol configuration, as detailed in Supplementary Materials, Section “DNN-based oPMN counting: Training and Assessment”.

#### 2.3.2. AI-Powered oPMN Counting

Although salivary oPMNs have been observed using microscopy in prior studies, there remains a lack of standardized, large-scale, annotated image datasets suitable for training and benchmarking AI models for oPMN detection and quantification. In contrast, extensive annotated datasets and AI pipelines exist for blood white blood cells, highlighting a clear gap for AI-enabled analysis of oPMNs in saliva. This necessitated the implementation of the isolation protocol described in Supplementary Materials Section “DNN-based oPMN counting: Training and Assessment”, followed by manual acquisition of Brightfield microscopy images. Trained biologists annotated these images using a graphical labeling tool [128], differentiating oPMNs from non-oPMNs.

The resulting dataset comprises high-resolution images (1080 × 1920 pixels), with a total of 4617 oPMNs annotated using bounding boxes in YOLO format [98]. Epithelial cells and debris were excluded from labeling and were treated as background during training. To enhance model performance, we employed transfer learning by initializing the YOLOv4 model with weights pre-trained on the MS COCO dataset [129,130,131,132,133,134]. The dataset was randomly partitioned into training, validation, and test subsets. Model performance was assessed using three standard metrics, including sensitivity, precision and F1 score, as defined in the Supplementary Materials, section of Optimization and Quantification Methods. To minimize false positives and enhance diagnostic accuracy, we applied a confidence threshold, retaining only predictions with class probabilities exceeding 95%. The image dataset was divided into independent training and test subsets, with 80% of the images used for model training and the remaining 20% reserved exclusively for testing. The data were partitioned at the patient/sample level to ensure that images originating from the same patient/sample were not included in both subsets, thereby preventing data leakage between the training and test datasets. The reported sensitivity, precision, and F1 score were calculated exclusively using the held-out test dataset.

A complete front-end and back-end infrastructure was developed to facilitate remote oPMN detection in clinical environments, as detailed in Supplementary Materials, Section “DNN based oPMN counting: Training and Assessment”. The back-end architecture follows a hybrid cloud model combining a secure private server, hosted at York University and equipped with high-performance GPUs, with a public cloud interface using AWS EC2 instances for scalability and accessibility. Inference tasks are handled by a YOLOv4-based AI engine implemented in Darknet, and all communications are secured via OpenVPN and SSL/TLS protocols. A Redis-based communication bus enables efficient job handling and concurrent request management.

On the front-end, a desktop application developed in .NET allows clinics to upload bright-field microscopy images, annotate results, and input patient metadata. Additionally, a web application provides remote access to the same services without requiring direct VPN access, enabling broader clinical usability. The web interface allows users to upload image batches (see Figure S3 in the SI), visualize detection outcomes, and review analysis summaries, while maintaining data security and supporting future scalability.

#### 2.3.3. Reproducibility and Quantification Workflow Summary

To ensure reproducibility and standardization, the BioSA procedure consisted of six defined steps: (1) coating the Petri dish surface with a cornstarch layer to create a hydrophilic substrate for adhesion-based retention; (2) collecting a saliva rinse sample (20 mL) and aliquoting it onto the coated Petri dish; (3) adding phosphate-buffered saline (PBS) to rinse the sample and facilitate removal of loosely associated debris; (4) applying a wash and removing the supernatant to eliminate non-adherent material while retaining adhered oral polymorphonuclear neutrophils (oPMNs) on the surface; (5) acquiring brightfield images from multiple non-overlapping fields of view per sample (>10 fields) using a 20× objective; and (6) estimating oPMN concentration by counting oPMNs in each field of view, computing the average count across images, and scaling by the ratio of Petri dish surface area to the calibrated field-of-view area (derived from pixel size). For the 20× objective used in this study, the effective field-of-view area was approximately 0.64 mm^2^. Final oPMN concentrations were reported as cells per 20 mL of saliva rinse.

## 3. Results and Discussions

This study was approved by the institutional ethics boards at York University and the University of Toronto, under the supervision of the Principal Investigators. Informed consent was obtained from all participants before their involvement in the experiments.

### 3.1. Validation of BioSA: Isolation and Quantification

Figure 3A(i) and Figure 3A(iii) present fluorescence microscopy images of a saliva rinse sample before and after the isolation process, respectively, while Figure 3A(ii) and Figure 3A(iv) show the corresponding brightfield microscopy images of the same samples. As observed in these images, unprocessed saliva contains a heterogeneous mixture of small oPMNs and large epithelial cells, illustrating the challenge of direct oPMN identification and underscoring the need for an effective isolation strategy. Figure 3B(i) shows a representative brightfield image of the saliva sample prior to isolation, in which oPMNs, epithelial cells, cellular debris, and surface-related artifacts on the HCPD are all present. Because oPMNs, epithelial cells, and background elements can exhibit overlapping size ranges, similar contrast, and comparable morphological features in brightfield images, accurate identification of oPMNs in raw, unprocessed samples is inherently difficult.

To address this limitation, we applied the BioSA adhesion-based isolation method, which selectively enriches oPMNs on the HCPD surface while removing the majority of epithelial cells and debris. The resulting improvement in sample purity is evident in the post-isolation brightfield image shown in Figure 3B(ii), where oPMNs are more clearly distinguishable from the background. Building on this isolation step, we subsequently applied an AI-based detection model to further improve the precision and consistency of oPMN identification, as illustrated in Figure 3B(iii).

To evaluate the performance of the AI model, Figure 3B(iii) presents representative examples from the held-out test dataset (see Supplementary Materials, Section “DNN-based oPMN counting: Training and Assessment”). Across this dataset, the model analyzed 505 detections, yielding 488 true positives, 17 false positives, and 9 false negatives. This corresponds to a sensitivity of 0.98, indicating that the model successfully identified 98% of annotated oPMNs, and reflects strong recall performance within the constraints of the available dataset. A precision of 0.97 indicates that the majority of detected objects were true oPMNs, demonstrating that the model effectively minimizes misclassification of non-oPMN components such as epithelial cells or debris. The resulting F_1_ score of 0.97, representing the harmonic mean of sensitivity and precision, indicates a balanced performance with a limited trade-off between detecting true oPMNs and restricting false detections.

These results support the robustness of the manual annotation process and confirm that the BioSA isolation protocol provides sufficiently clean and consistent input data for AI-based analysis. The relatively low number of false positives further supports the use of a confidence threshold greater than 95%, which was selected to prioritize reliability and reduce spurious detections. Together, these findings demonstrate that the combination of adhesion-based isolation and AI-assisted detection enables reliable oPMN quantification from brightfield microscopy images, forming a solid technical foundation for subsequent analyses, while recognizing that performance metrics reflect results obtained within this experimental and dataset-specific context.

### 3.2. Characterization: Hydrophilic Adhesion

**Figure 4:**
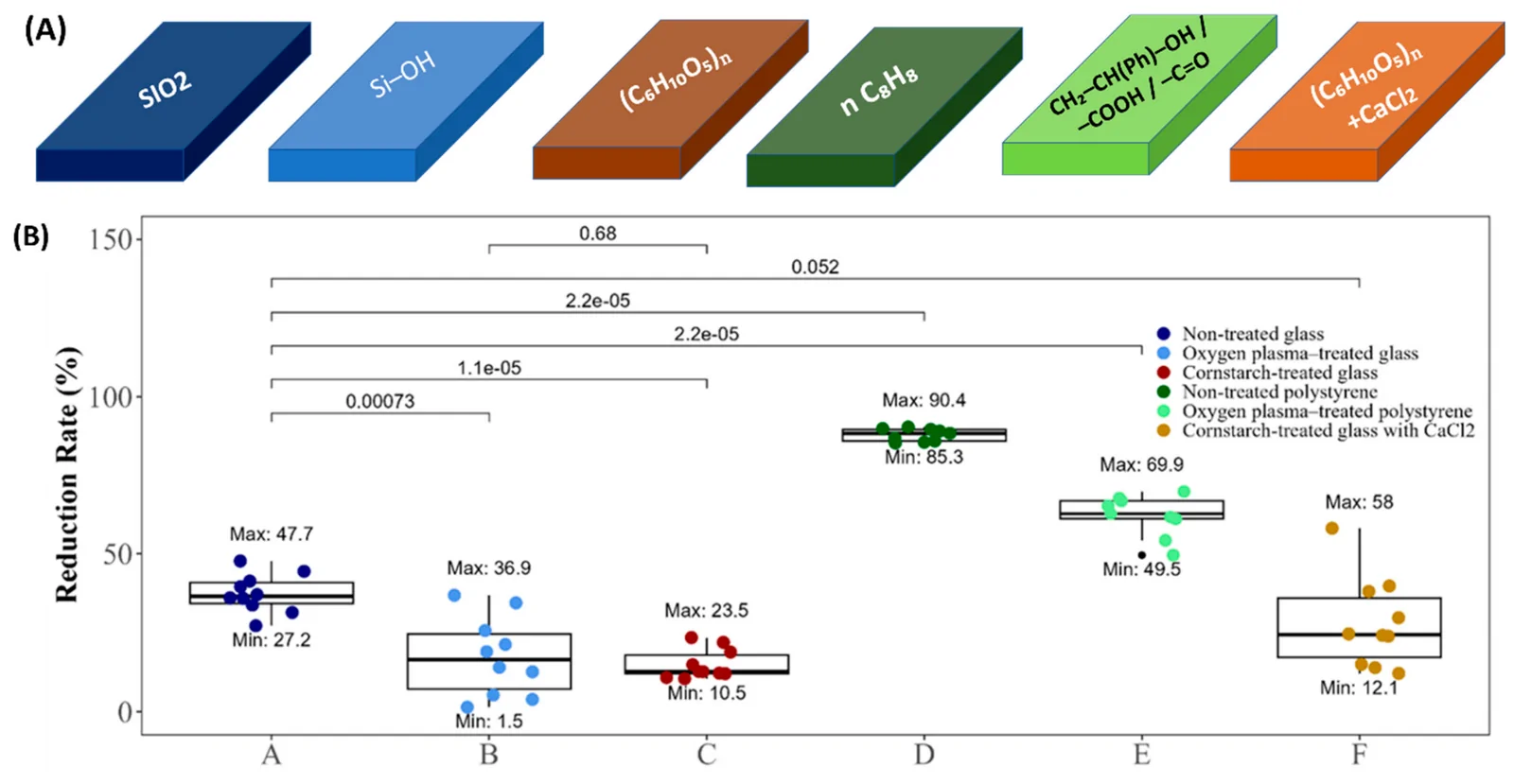
Hydrophilic adhesion (**A**) Surfaces used in the experiments: bare glass (SiO_2_), oxygen plasma–treated glass (Si–OH modified SiO_2_), polystyrene (–CH_2_–CH(Ph)–)_n_ from styrene (C_8_H_8_), oxygen plasma–treated polystyrene (introducing –OH, –COOH, and –C=O groups), CS ((C_6_H_10_O_5_)_n_), and CS crosslinked with CaCl_2_. (**B**) Box Plot of oPMN levels before and after isolation on six different surfaces: bare glass (A), oxygen plasma-treated glass (B), CS-treated glass (C), polystyrene (D), oxygen plasma polystyrene (E), and CS-treated glass and CaCl_2_ (F). Number format: 1.1e−5 is equivalent to $1.1\times 10^{-5}$, or 0.000011.

Two sets of experiments were conducted using bare and hydrophilic-coated (HC) substrates to evaluate the role of surface adhesion in oPMN isolation using the BioSA method. As summarized in Tables S6–S9 (SI), each experimental condition consisted of 10 independent experimental groups, with three technical replicates per group and 10 brightfield microscopy images acquired per replicate. A trained deep neural network (DNN) model was used to automatically quantify oPMNs in all acquired images to ensure consistency across conditions. The aggregated results were visualized using box plots (Figure 4), enabling direct comparison of oPMN retention across different surface treatments.

These experiments were performed on substrates with distinct surface chemistries, including bare glass (SiO_2_), oxygen plasma–treated glass, cornstarch (CS)-coated glass, polystyrene, oxygen plasma–treated polystyrene, and CS-coated glass in the presence of CaCl_2_, as shown in Figure 4A. Silicon dioxide (SiO_2_) consists of a network of Si–O–Si bonds arranged in a tetrahedral structure; upon exposure to moisture, surface hydrolysis generates hydroxyl (–OH) groups that increase hydrophilicity. Cornstarch, a natural polysaccharide composed of amylose and amylopectin ((C_6_H_10_O_5_)_n_), serves as a hydrophilic, biodegradable coating material. In contrast, polystyrene (PS), formed by polymerization of styrene monomers, is intrinsically hydrophobic due to its non-polar phenyl groups. Oxygen plasma treatment enhances surface wettability by introducing polar functional groups: on glass, it increases surface hydroxylation, while on polystyrene it introduces oxygen-containing groups such as –OH, –COOH, and –C=O, thereby improving hydrophilicity and biocompatibility.

As shown in Figure 4B, oPMN counts decreased after the isolation process across all surface types; however, the magnitude of reduction varied substantially by substrate. Bare glass exhibited a pronounced decrease, whereas hydrophilic-coated surfaces showed more modest reductions, indicating improved oPMN retention. CS-coated substrates consistently demonstrated the smallest reductions, supporting the role of hydrophilic interactions in stabilizing oPMN adhesion. Individual measurements are displayed as jittered points to illustrate variability across replicates and to capture the distribution of values within each condition.

To strengthen the statistical interpretation of Figure 4, oPMN retention was evaluated using paired before–after measurements for each surface condition. Reduction and retention rates were calculated for each replicate, and group differences were assessed using both parametric and non-parametric approaches. Because normality and equal-variance assumptions were not fully satisfied, Kruskal–Wallis testing, Welch-based comparisons, and corrected pairwise analyses were applied. The underlying raw measurements are provided in Tables S6 and S7 (Supplementary Information), with derived metrics summarized in Table S7.1 and group-level statistics in Table S7.2. Assumption testing results are reported in Tables S7.3 and S7.4, and global group comparisons using ANOVA/Welch and Kruskal–Wallis methods are presented in Tables S7.5 and S7.6. A visual summary of paired changes is shown in Figure S9.

Across conditions, mean reduction rates ranged from approximately 15% for CS-coated glass to nearly 88% for non-treated polystyrene, with global testing confirming a highly significant effect of surface type (ANOVA *p* < 10^−24^; Kruskal–Wallis *p* < 10^−8^). Pairwise comparisons (Tables S7.7–S7.9) further showed that CS-coated glass exhibited significantly lower reduction rates compared to most alternative surfaces.

Together, these analyses confirm that surface chemistry has a statistically significant effect on oPMN retention. CS-coated glass consistently exhibits the highest retention (lowest reduction), whereas non-treated and polystyrene-based substrates show substantially greater cell loss after washing. These findings highlight the critical role of surface hydrophilicity in oPMN adhesion and provide quantitative support for the biologically inspired isolation mechanism underlying the BioSA platform.

It is noteworthy that calcium ions play a critical role in neutrophil activation, where IL-8 triggers PLC via GPCRs, leading to the release of DAG and IP_3_, subsequent calcium flux, and PKC activation—essential steps in neutrophil adhesion [93,94]. Similarly, epithelial adhesion relies on calcium-dependent CAMs such as cadherins, which mediate cell–cell junctions [95,96]. However, since both cell types depend on calcium, calcium chloride does not selectively enhance neutrophil attachment or support their isolation. Instead, despite this fact, the adhesion of oPMNs has increased in some of the group experiments observed in Figure 4B. Adhesion in this study is primarily mediated by hydrophilic interactions between cell surface CD markers and the treated substrate, a mechanism rooted in the cells’ biophysical properties rather than calcium signaling.

#### 3.2.1. Role of CD Marker in Adhesion

**Figure 5:**
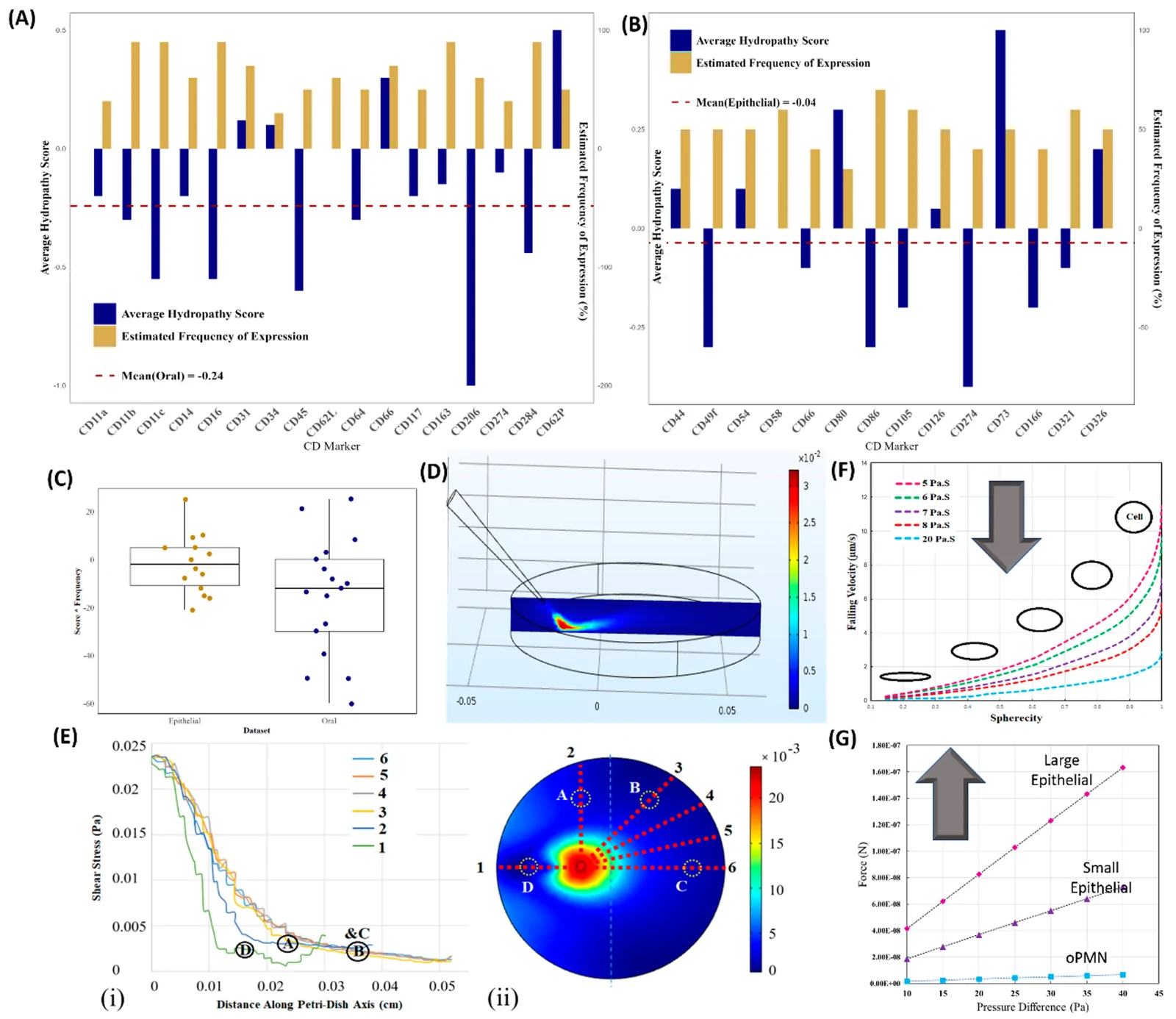
Computational Analysis of Hydrophilicity and Adhesion Dynamics. (**A**) Adhesion scores of oPMNs and (**B**) epithelial cells based on CD marker hydrophilicity and expression. (**C**) Comparative box plot of Score × Frequency between oral and epithelial markers reveals greater variability in oral tissue, suggesting diverse functional roles. (**D**) COMSOL version 6.4 simulation of falling velocity, assuming spherical shape for neutrophils (sphericity = 1) and non-spherical shape for epithelial cells (sphericity < 1), shows that neutrophils settle faster across viscosities ranging from 5 to 20 Pa·s due to their morphology. (**E**) Shear stress distribution at the bottom of a Petri dish induced by pipetting at a 45° angle: (**i**) shear stress along selected lines (1–6); (**ii**) contour plot of shear stress, pipette position, and resulting flow field. (**F**) Simulation of the vertical force magnitude applied to oPMNs and epithelial cells during pipette-induced suction. The simulation demonstrates that epithelial cells experience a higher suction force compared to oPMNs. (**G**) Force comparison of neutrophils (square) and epithelial cells (triangular) on a Petri dish surface shows epithelial cells, with a larger surface area and tendency to aggregate (pink), experience higher suction forces, relevant for cell separation.

Figure 5 illustrates that increased surface hydrophilicity is associated with enhanced is cell adhesion and that the presence of hydrophilic CD markers may further contribute to this biologically inspired adhesion mechanism. To examine this relationship, a computational hydrophilicity analysis was performed, as described in Section 2. Figure 5A presents the average hydropathy scores together with estimated expression frequencies of CD markers on oral neutrophils. Markers with negative hydropathy scores (e.g., CD11b and CD18) are more hydrophilic and are therefore expected to contribute more strongly to adhesion on hydrophilic surfaces. The red dashed line indicates the weighted mean hydropathy score and is shown for reference.

Figure 5B presents the corresponding analysis for epithelial cells. In this case, markers such as CD66 and CD105 exhibit higher hydrophobicity, whereas markers including CD49f and CD274 display more hydrophilic characteristics. Highly expressed markers such as CD44 and CD326 are likely to play important roles in epithelial adhesion and signaling. Together, these observations suggest that both surface chemistry and CD marker composition influence cell–substrate interactions.

The comparative box plot shown in Figure 5C summarizes the distribution of Score × Frequency values for CD markers across oral and epithelial tissues. In epithelial tissue, the median value is slightly above zero with relatively low variance, indicating a more uniform hydropathy–expression profile. This pattern is consistent with the stable barrier function of epithelial tissue. In contrast, oral tissue exhibits a broader distribution with a lower median value and several outliers, reflecting greater variability in CD marker hydrophilicity and expression. This variability is consistent with the adaptive nature of oral tissue, which is continuously exposed to a dynamic microbial environment and mechanical perturbations.

Overall, these results support the interpretation that epithelial tissues tend to exhibit more uniform adhesion-related properties, whereas oral tissues display a more diverse set of CD marker characteristics that may enable flexible immune and adhesion responses. These conclusions are based on descriptive computational analyses, and future studies incorporating formal statistical testing and larger datasets will be required to assess the significance of the observed trends quantitatively.

#### 3.2.2. Role of Physical Interactions in Adhesion

After loading a saliva sample containing both neutrophils and epithelial cells, pipetting is used to remove debris and other components, including epithelial cells, in order to purify the sample for subsequent image-based analysis. To better understand the physical mechanisms underlying this separation process, a computational fluid dynamics simulation was conducted to examine how pipetting influences shear stress distribution within a Petri dish. COMSOL Multiphysics (Version 5.5) was used to visualize the effects of pipette-driven flow on the substrate surface. The primary objective of this simulation was to qualitatively assess how shear stress generated during pipetting contributes to epithelial cell removal while preserving adherent neutrophils.

As shown in Figure 5D, the simulations indicate that shear stress originates near the point of fluid introduction and propagates radially toward the edges of the Petri dish. The highest shear stress is localized near the pipette tip, forming a high-stress region. With increasing distance from the point of fluid impact, the shear stress becomes more uniform, as illustrated by regions A, B, C, and D in Figure 5E. These regions experience relatively stable and moderate shear stress levels, making them suitable for microscopy imaging and reliable cell counting. The qualitative simulation suggests that imaging in these regions minimizes variability introduced by localized flow disturbances.

Quantitative estimates of applied forces derived from the simulation are shown in Figure 5F. For simplicity, the fluid was modeled as water, with an inlet mass flow rate of 25 µL/s and a pipette tip positioned 2 cm above the Petri dish surface. The corresponding CAD geometry is shown in Figure S6 in the SI. The average shear stress values within the predefined circular regions were approximately 0.0031 Pa (A), 0.0025 Pa (B), 0.0028 Pa (C), and 0.0024 Pa (D), as shown in Figure 5E(i,ii). These values indicate the presence of a shear stress field sufficient to apply forces to epithelial cells and the surrounding saliva matrix, thereby facilitating sample purification prior to neutrophil counting.

To further examine experimental observations showing that neutrophils attach to the substrate more rapidly than epithelial cells, an additional COMSOL simulation was performed to investigate differences in cell settling behavior. In this model, neutrophils were represented as spherical particles, while epithelial cells were modeled as non-spherical particles characterized by a lower sphericity factor, as illustrated in Figure 5F. The simulations demonstrate that neutrophils and epithelial cells settle at different rates due to differences in shape and effective drag. Specifically, spherical neutrophils exhibit higher settling velocities than irregularly shaped epithelial cells, allowing them to reach and adhere to the Petri dish surface more quickly.

Another important physical factor contributing to cell separation is the suction force applied during pipetting. During suction, cells with larger surface areas experience greater hydrodynamic forces, which promotes their detachment compared with smaller, more compact cells such as neutrophils. COMSOL simulations shown in Figure 5G illustrate that epithelial cells, due to their larger surface area and tendency to aggregate, are subjected to higher forces during suction, enabling their preferential removal from the surface. These simulations provide a qualitative comparison of the forces acting on epithelial cells versus neutrophils during the suction process and help explain the observed selective removal of epithelial cells.

The flow contours shown in the inset of Figure 5G depict circular cells representing neutrophils under the pressure field generated by pipette suction. The governing equations and simulation parameters are detailed in the Supplementary Materials section “COMSOL simulation” (Figures S6–S8). The modeled domain represents an elemental cylindrical section of the Petri dish with open boundary conditions along the side walls and a no-slip condition at the substrate surface. Pressure was applied at the top boundary to simulate pipette-induced suction. Pressure contours for the neutrophil model, epithelial cell model, and aggregated epithelial cells are presented in Figure 5G. Additional methodological details are provided in the Supplementary Information.

Overall, these simulations support a physically plausible mechanism in which differences in adhesion strength, settling behavior, and hydrodynamic forces collectively contribute to the selective removal of epithelial cells while retaining neutrophils during the BioSA isolation process. The simulations are intended to provide qualitative insight into the separation mechanism rather than exact quantitative prediction of forces in experimental conditions.

### 3.3. Optimization of BioSA Parameters

To optimize the concentration of the HCM, five concentrations (0.2, 0.4, 0.6, 0.8, and 1.0 mg/mL) were evaluated, with each condition tested in triplicate, resulting in a total of 15 experiments. Images obtained from each trial were analyzed using the AI-assisted oPMN counting pipeline. Among the tested concentrations, 0.6 mg/mL consistently yielded the highest oPMN counts and was therefore selected as the optimal HCM concentration for subsequent experiments.

In addition, settling times of 5, 10, 15, and 20 min were examined across multiple experimental groups, with three replicates per condition (12 tests in total). A settling time of 15 min (T1 = 15 min, T2 = 3 min) produced the highest oPMN yield, indicating that this duration provides sufficient time for stable oPMN adhesion while limiting nonspecific attachment of epithelial cells (see Supplementary Materials, Section “DNN-based oPMN counting: Training and Assessment,” Figures S2–S5 and Tables S2–S5).

The effect of ionic strength in the WB on epithelial cell removal and oPMN retention was also evaluated by diluting WB with water to final concentrations of 20%, 40%, 60%, 80%, and 100%. Each condition was tested in triplicate, for a total of 15 experiments. The results demonstrated that undiluted (100%) WB consistently provided the highest oPMN retention following the washing step. This observation suggests that higher ionic strength supports more effective separation, likely by maintaining surface hydrophilicity and stabilizing oPMN–substrate interactions, consistent with prior reports [135,136].

Finally, the influence of WB volume on oPMN retention was assessed using wash volumes of 5, 10, 15, 20, and 25 mL. Among these conditions, a volume of 15 mL resulted in the highest retained oPMN counts after isolation and was selected as the optimal wash volume. The complete dataset and corresponding statistical analyses supporting these parameter optimizations are provided in the Supplementary Materials, Section “Concentration of HCM”.

### 3.4. Limited Clinical Tests

Both the BioSA and HEMO methods were applied to each clinical sample to quantify oPMN levels. Each result’s point of BioSA presents oPMN counts derived from ten brightfield images per sample after isolation. The results reveal a wide range of oPMN concentrations, from approximately 500,000 to over 6 million cells per 10 mL, consistent with a continuum spanning healthy gingival conditions to severe periodontitis 112. Notably, the average oPMN counts obtained using the BioSA platform closely match those measured using the conventional HEMO method, supporting the quantitative validity of the proposed approach.

**Figure 6:**
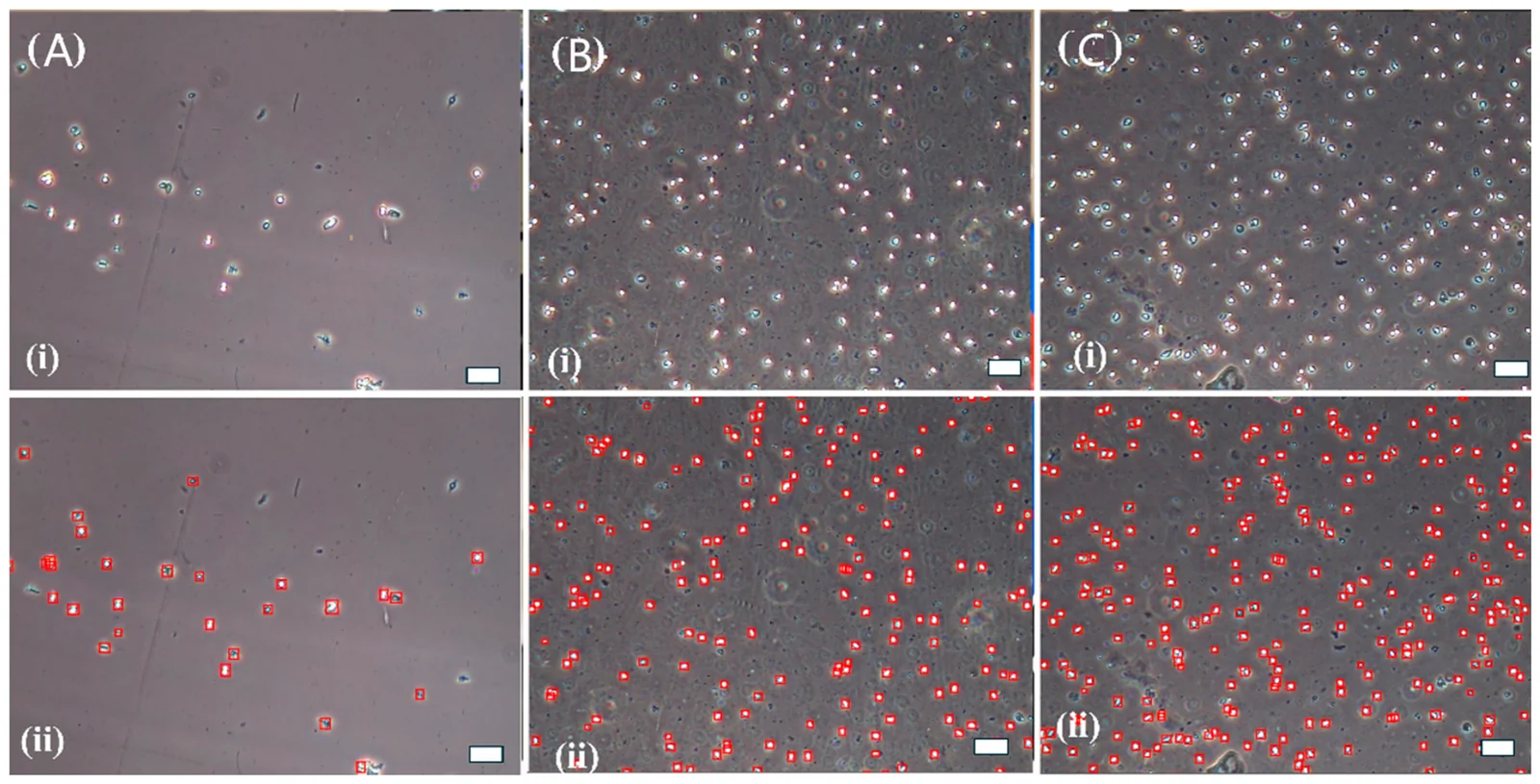
Clinical test: (**A**–**C**) present three different groups of brightfield microscopy images of samples from the healthy, moderate, and severe groups, shown before (**i**) and after (**ii**) AI-based detection and analysis. Scale bar: 10 µm.

Figure 6A–C, representative of hundreds of images, show brightfield microscopy images of representative clinical samples from the moderate and severe groups. In each pair of panels, the performance of the AI-based analysis can be assessed by comparing the raw brightfield image (i) with the corresponding AI-generated segmentation and detection results (ii).

Quantitative oPMN measurements obtained using the BioSA pipeline were further compared with those from the conventional HEMO method, as shown in Figure 6A, with zoomed-out insets illustrating agreement across the five PD stages shown in Figure 6B,C. This comparison reveals a strong linear relationship between the mean values produced by the two methods (see Supplementary Materials, Section “Clinical Data”). BioSA mean values, calculated from ten images per sample and shown in dark blue, were plotted against the corresponding HEMO values on the y-axis. As shown in Figure 6A, the data points align closely along a straight line, with a standard deviation of approximately 2.3%, indicating high concordance and a strong linear correlation between the two measurement techniques.

**Figure 7:**
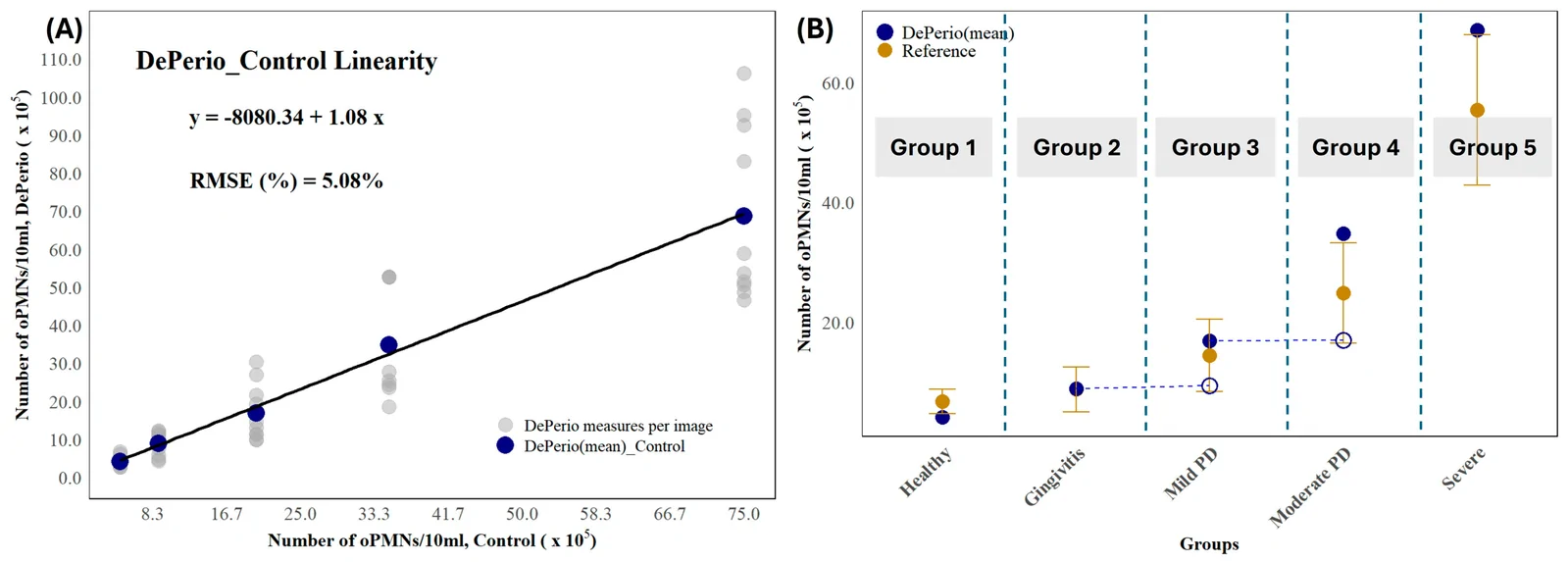
Clinical results: (**A**) Linearity assessment and prediction of various OIL levels with subgroups 1–5, (**B**) Comparison of mean oPMN counts measured by DePerio (blue) and the reference method (gold) across five periodontal clinical groups: healthy, gingivitis, mild periodontitis (PD), moderate PD, and severe PD. Error bars indicate the variability of the reference measurements. Open circles and dashed blue lines indicate the proposed boundaries between adjacent OIL groups.

Although some variation in oPMN counts was observed across individual BioSA images—attributable to the large substrate area and heterogeneous spatial distribution of cells—the averaged BioSA values consistently aligned with the corresponding HEMO-derived counts. In addition, analysis showed that averaging more than ten images per sample resulted in negligible improvement in measurement accuracy, supporting the use of ten images as a practical and efficient balance between robustness and throughput. As shown in Figure 7A and detailed in the SI, BioSA measurements, calculated from ten-image aggregates, were compared with HEMO counts using linear regression. The fitted regression line (y = 8080.34 + 1.08x) exhibits a slope close to 1.0, indicating strong proportional agreement between BioSA and the reference method across the measured range. While most BioSA data points cluster closely around the regression line, modest deviations are observed at higher oPMN concentrations, which are likely attributable to biological variability rather than systematic measurement error. Overall, these results demonstrate that BioSA provides oPMN quantification that is highly consistent with HEMO across a broad range of inflammatory states represented in this clinical cohort.

To complement the visual assessment shown in Figure 7A, quantitative agreement between BioSA and HEMO was further evaluated using linear regression, correlation analysis, and Bland–Altman statistics. As reported in Table S20, the slope and intercept are provided with 95% confidence intervals, along with R^2^, RMSE, MAE, MAPE, and Bland–Altman bias and limits of agreement. Together, these metrics provide a comprehensive assessment of agreement between the two methods within the context of the available clinical data.

#### Prediction of OIL Level

This study uses oPMN levels as quantitative biomarkers for estimating OIL, which can be approximately related to multiple statistical groups described in Section 2. In this statistical prediction, OIL levels are mapped into five groups, simply said, PD categories, which could alternatively be mapped into fewer (e.g., high and low OIL) or more granular groupings. The selection of five groups was made to align conceptually with the five standard stages of PD defined by the 2017 World Workshop guidelines. These mappings are intended to provide an interpretable framework for relating oPMN measurements to clinical inflammatory states, rather than to establish definitive diagnostic classifications in this study or in future clinical practice. Building on the measurements presented in Figure 7B, Tables S18 and S19 summarize the distribution of human subjects across the five PD categories in this cohort. The number of subjects in each group was 6 (group 1), 5 (group 2), 4 (group 3), 4 (group 4), and 2 (group 5), reflecting an uneven group distribution and a limited sample size, particularly in the severe category.

To define the five PD stages used in this analysis, oPMN thresholds were derived using a combined data-driven and literature-supported approach. First, an unsupervised k-means clustering algorithm (k = 5) was applied to the full oPMN dataset to identify natural groupings corresponding to different levels of OIL. Samples were assigned to the nearest cluster centroid using Euclidean distance, and centroids were iteratively updated until convergence to minimize the within-cluster sum of squares (WCSS). Operational thresholds (e.g., 0.5 × 10^6^ and 2 × 10^6^ cells per 10 mL [114]) were defined at inter-centroid midpoints and therefore arose directly from the observed oPMN distribution within this cohort rather than from manual tuning. Cluster assignments and centroid values are reported in Supplementary Materials. Exploratory k-means clustering identified five data-driven OIL strata within the present cohort. These strata were used for hypothesis-generating analysis and should not be interpreted as validated diagnostic stages or clinical classifications.

Interpretation of the resulting clusters was guided by two criteria: (i) the clinical PD-stage composition within each cluster and (ii) a monotonic increase in mean oPMN counts across clusters from health to severe inflammation. These data-driven clusters were then compared with previously published oPMN ranges reported in clinical studies that established salivary neutrophil counts as biomarkers of periodontal status, including quantifying oral inflammatory load: Oral neutrophil counts in periodontal health and disease [111], quantitation of the cellular content of saliva and buccal swab samples [73], and OIL (neutrophil counts) as oral health biomarkers [114]. The thresholds obtained from k-means clustering were broadly consistent with the reproducible oPMN ranges reported in these studies.

Agreement between BioSA-derived statistical PD category assignments and HEMO-based classifications was evaluated across 30 validation samples. Within this dataset, category-level assignments were concordant between the two methods. However, given the limited cohort size and small number of subjects in some PD categories, particularly the severe group, this agreement should be interpreted as preliminary and descriptive. Larger, independent cohorts will be required to robustly validate these thresholds and to assess the generalizability of OIL-based PD stage prediction.

Additional statistical analyses supporting the structure of the five data-driven oPMN groups are reported in the Supplementary Information. Shapiro–Wilk tests indicated approximate normality within individual groups, while Levene’s test demonstrated unequal variances across categories. This combination of findings supports the use of a clustering-based approach for defining oPMN-derived inflammatory categories, rather than reliance on variance-dependent parametric classification methods.

### 3.5. Repeatability and Sensitivity: BioSA Versus HEMO

**Figure 8:**
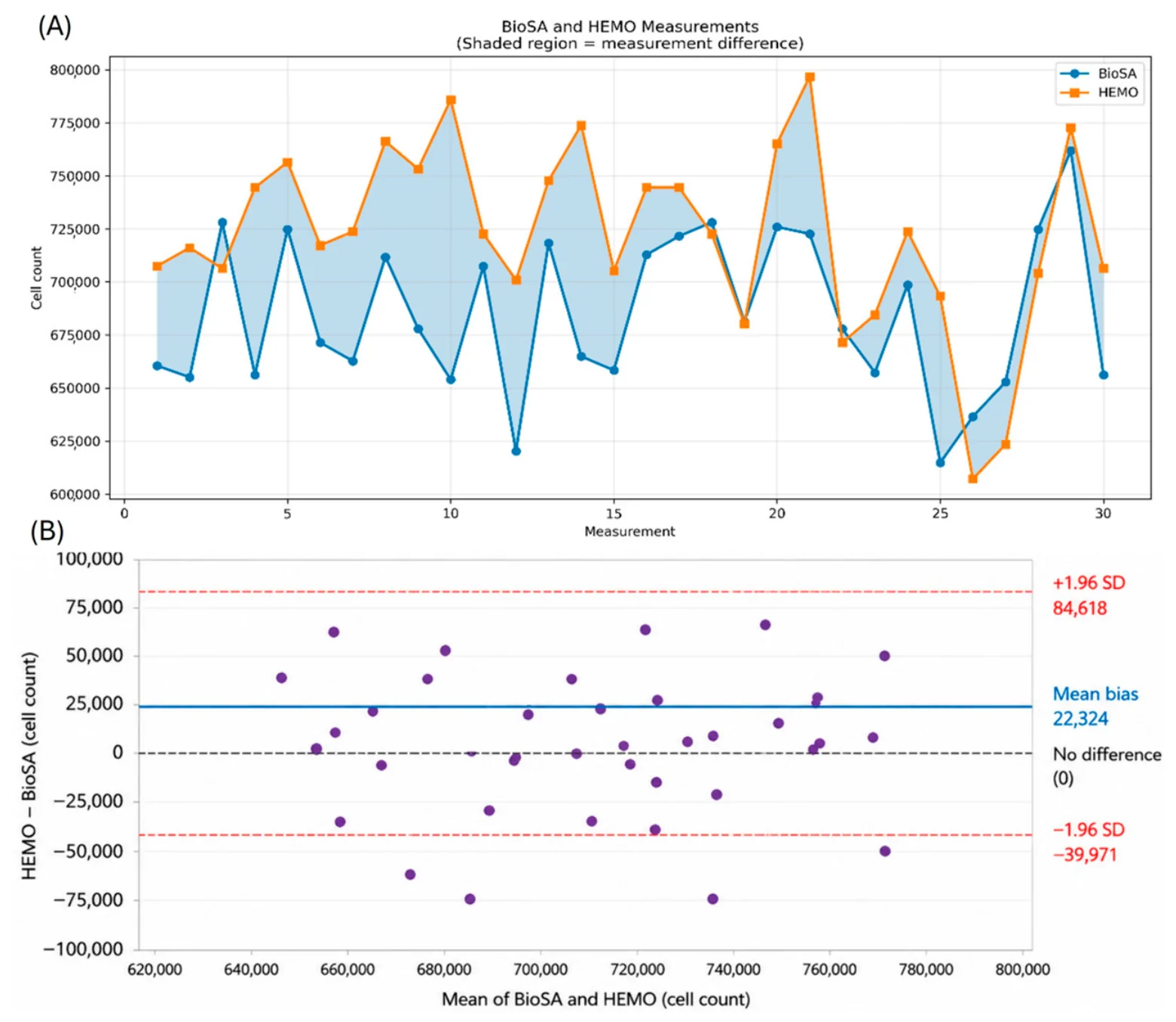
(**A**) Paired comparison of BioSA and HEMO oPMN counts across 30 test days. The analysis illustrates the consistency of the paired measurements and the differences between the two measurement approaches. The blue shaded region represents the difference between the paired BioSA and HEMO measurements. (**B**) Bland–Altman analysis of agreement between BioSA and HEMO oPMN measurements. The difference between paired HEMO and BioSA measurements (HEMO − BioSA) is plotted against the mean of the two measurements. The mean bias was 22,324 cells, indicating that HEMO measurements were, on average, 22,324 cells higher than the corresponding BioSA measurements. The 95% limits of agreement, calculated as the mean bias ± 1.96 standard deviations (SD), ranged from −39,971 to 84,618 cells. The solid blue line represents the mean bias, the red dashed lines represent the upper and lower 95% limits of agreement, and the black dashed line indicates zero difference between the two methods.

To evaluate the repeatability and sensitivity of the proposed method, 30 test groups were conducted over 30 consecutive days (one per day) using the BioSA platform, with three technical replicates per day, as shown in Figure 8A. An equal number of measurements were performed on the same samples using the standard HEMO method as a reference, enabling direct paired comparison and assessment of day-to-day consistency between the two approaches. To further assess agreement between BioSA and HEMO beyond correlation analysis, a Bland–Altman analysis (shown in Figure 8B) was performed using the paired measurements. The mean difference (HEMO − BioSA) was 22,324 cells, indicating that HEMO measurements were, on average, higher than the corresponding BioSA measurements. The 95% limits of agreement ranged from −39,971 to 84,618 cells, reflecting the variability in individual differences between the two methods. Most paired differences fell within these limits of agreement, although several measurements showed larger positive or negative deviations. These results demonstrate a systematic positive bias toward higher HEMO measurements and further characterize the agreement between BioSA and HEMO beyond the association assessed by correlation analysis.

Agreement between BioSA and HEMO was assessed using a paired method-comparison framework across the 30 test days. The complete paired dataset is provided in Table S18.1 (Supplementary Information), which reports BioSA and HEMO measurements, paired differences, and error metrics for each day. Summary agreement statistics—including mean difference, 95% confidence intervals, mean absolute error (MAE), root mean square error (RMSE), and correlation coefficients—are presented in Table S18.2. Bland–Altman bias and limits of agreement are detailed in Table S18.3, while regression analysis results describing the relationship between BioSA and HEMO are reported in Table S18.4. Corresponding visualizations are provided in Figures S10–S12, including the Bland–Altman plot (Figure S10), paired difference plot across test days (Figure S11), and regression-based agreement analysis (Figure S12).

Quantitatively, BioSA showed a mean difference of approximately −3.7 × 10^4^ oPMNs relative to HEMO, indicating a small but consistent underestimation (Table S18.2). Error-based metrics remained stable across measurements, with MAE ≈ 4.5 × 10^4^ and RMSE ≈ 5.5 × 10^4^, while correlation analysis demonstrated a statistically significant positive association between methods (Pearson r ≈ 0.50, *p* < 0.01). Bland–Altman limits of agreement further confirmed that paired differences remained within a consistent and bounded range (Table S18.3), supporting stable agreement between the two measurement approaches.

As shown in Figure 8, BioSA and HEMO exhibit similar temporal patterns across test days, with a relatively stable difference between methods. Paired analysis indicates that BioSA slightly underestimates HEMO on average; however, this bias remains consistent across measurements. The distribution of paired differences (Figure S11) shows no systematic drift over time, while the Bland–Altman analysis (Figure S10) confirms stable limits of agreement without strong dependence on measurement magnitude. Regression analysis (Figure S12) further demonstrates a consistent positive association between the two methods.

Overall, these results demonstrate that BioSA provides reproducible and reliable oPMN quantification relative to HEMO, while transparently characterizing the magnitude and direction of systematic differences between methods.

### 3.6. AI Framework Performance

Details of the DNN model, including its training procedure and performance characterization, are provided in the Supplementary Materials under the section titled “DNN-based oPMN Counting: Training and Assessment.” Figure S5 presents the confidence histogram for model predictions, and Table S3 summarizes the performance of the deep learning–based oPMN detection using standard metrics, including precision and recall. In addition, the response time of the custom-designed framework for transferring and processing each group of images was measured to be less than 10 s. Together, these results demonstrate that the developed hardware and software framework enables rapid and reliable AI-assisted oPMN quantification and is suitable for efficient estimation of OIL within the scope of this study.

### 3.7. Toward Fully Automated oPMN Quantification

**Figure 9:**
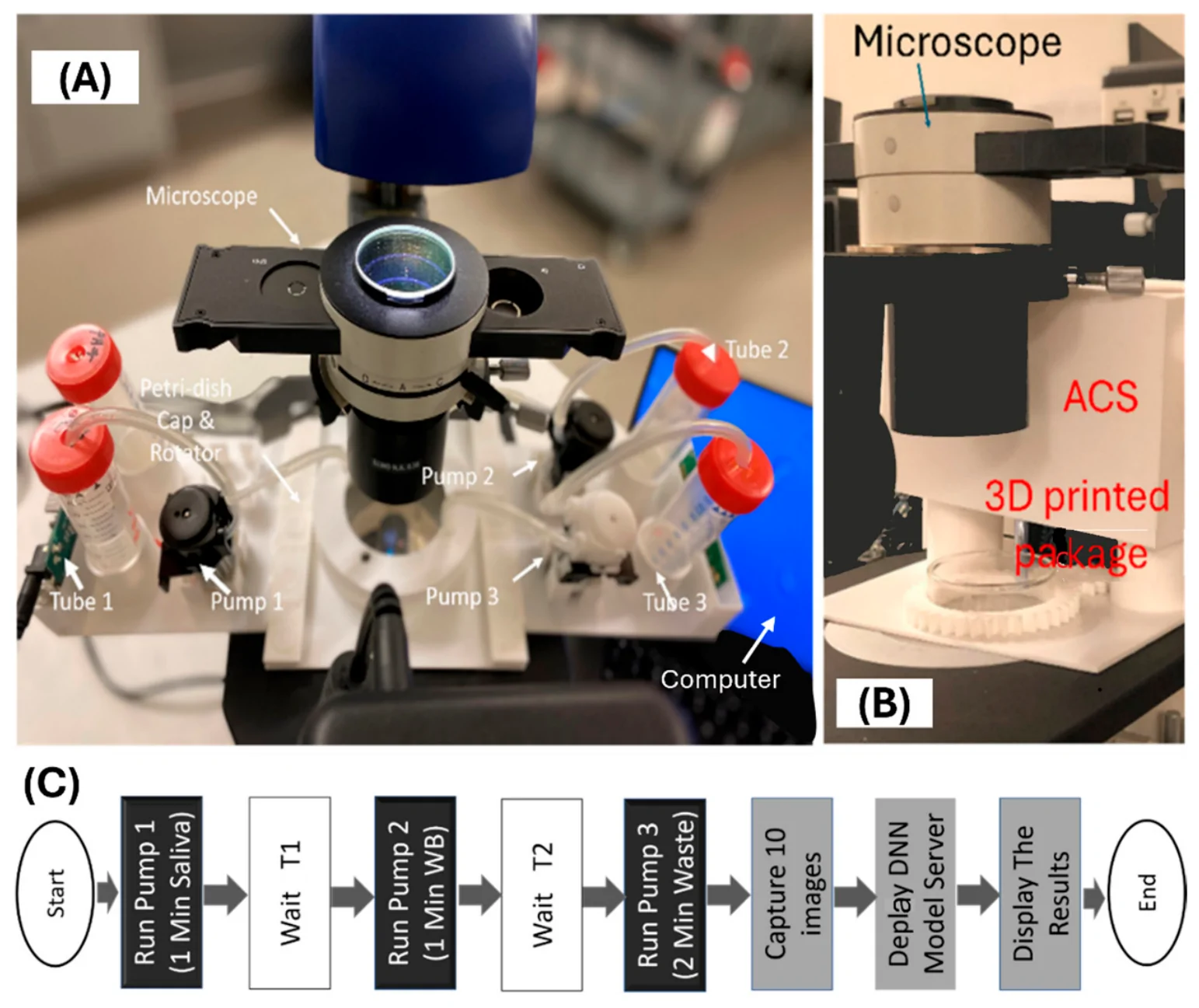
Fluidic handling and image positioning control system shown from the top (**A**) and side (**B**) perspectives, and (**C**) the workflow and system operation.

The proposed BioSA can be implemented either through a manual protocol, as shown in Figure 2B, or via a low-complexity Automated Control System (ACS), shown in Figure 9A,B. The ACS was designed with a focus on simplicity to demonstrate its functionality and suitability for OIL quantification. The system includes minimal fluidic handling and image positioning components: four pumps connected to tubing for saliva, WB, sanitizer, and waste collection; a servo motor for rotational control of the Petri dish holder; and gears of the HCPD controlled by a microcontroller.

Additionally, three custom-printed components were installed on the microscope. A flowchart outlining the program workflow and system operation, based on the protocol shown in Figure 9B, is presented in Figure 9C. It is noteworthy that the data presented in this paper were obtained manually.

### 3.8. Graphical User Interface (GUI)

To facilitate practical use of the AI-assisted oPMN quantification workflow, we developed a web-based GUI that allows users to upload microscopy images, execute the trained model, and visualize the results through an intuitive dashboard. The current version of the GUI is hosted as a web application and can be accessed through any computer, laptop, or smartphone equipped with a standard internet browser. This platform provides cross-device accessibility without requiring software installation and enables users to view oPMN counts, segmentation overlays, and analysis logs in real time. Looking ahead, dedicated Android and iOS applications can be integrated to further streamline the workflow. A mobile app would allow direct smartphone-based image capture, on-device or cloud-assisted inference, and automated reporting of results. Such integration would support real-time monitoring in clinical, community, and field settings and represents a key future step toward a fully portable and user-friendly diagnostic ecosystem.

It is important to note that although systemic factors—including chemotherapy, autoimmune therapies, infections, and chronic diseases—may influence the total number of oPMNs present in saliva, these variations do not compromise the specificity of the assay. The adhesion-based capture and AI quantification methods target biophysical properties unique to oPMNs, ensuring accurate detection even when biological abundance fluctuates. As a result, oPMN levels remain a useful indicator of OIL, but interpretation should consider comorbid conditions that may independently affect neutrophil availability.

To contextualize the performance of BioSA, we provide a benchmarking comparison with commonly used oPMN enumeration methods, including fluorescence microscopy, flow cytometry, and Pap cytology methods. The comparative metrics—summarized in Supplementary Table S1—highlight the differences in required instrumentation, sample-processing complexity, cost, operator dependence, analysis time, and quantitative performance. This benchmarking illustrates the potential of BioSA as a low-cost, label-free, and automated alternative suitable for clinical and point-of-care settings.

## 4. Conclusions

This study builds on the previously reported DePerio framework for adhesion-based oPMN isolation and AI-assisted quantification and further investigates BioSA, with an emphasis on the mechanistic and quantitative characterization of the isolation process. BioSA isolates oPMNs by leveraging their preferential hydrophilic adhesion to a CS-coated glass surface, without additional recognition elements. Across more than 200 saliva-based experiments, this strategy consistently enriched oPMNs from complex oral samples. The transparent CS coating enables brightfield imaging and integration with a DNN for automated oPMN detection and quantitative assessment of OIL. The primary contribution of this work is the expanded mechanistic characterization of the biologically inspired adhesion-based isolation process, together with rapid, low-cost quantification of oPMNs as a measure of OIL, providing early, non-invasive insight into inflammation and risk stratification rather than serving as a standalone diagnostic tool. Accordingly, BioSA does not replace or modify the standard periodontitis staging and grading framework, as defined by the 2017 World Periodontal Workshop [7], which is based on established clinical and radiographic measures including clinical attachment loss, probing depth, bleeding on probing, radiographic bone loss, tooth loss, and complexity factors. These parameters are less sensitive at early stages, such as periodontal health and gingivitis, where tissue damage has not occurred, and conventional markers may be absent or inconsistent. In contrast, oPMNs reflect the host inflammatory response to microbial burden and may rise before structural damage, making oral inflammatory load (OIL), quantified via oPMN concentration, a complementary inflammation-linked cellular biomarker for screening, monitoring, and preliminary exploratory, data-driven inflammation stratification rather than definitive clinical staging.

BioSA enables rapid, reproducible quantification of oPMNs using brightfield imaging and AI, with preliminary results supporting its role in augmenting, not replacing, established diagnostic frameworks. The reported “five levels” represent a hypothesis-generating OIL stratification that requires validation in larger, independent cohorts before any linkage to clinical stage or grade. Repeatability and quantitative agreement were confirmed in 90 additional experiments, showing high precision and close correspondence with conventional oPMN counting. Reported sensitivity, precision, and F1 scores reflect cell-level oPMN detection against manual image annotations and should not be interpreted as clinical diagnostic performance. By exploiting intrinsic neutrophil adhesion without fluorescent labels or complex procedures, BioSA offers a simple, scalable approach.

Combined with AI-assisted analysis, the platform provides rapid and reproducible OIL assessment. From a clinical workflow perspective, BioSA may support longitudinal monitoring through repeatable OIL measurements, enable earlier intervention by flagging elevated inflammatory burden, assist front-end triage and maintenance planning, objectively track treatment response and rebound trends, and improve patient engagement and documentation through quantitative reporting over time. In summary, BioSA delivers low-cost, objective quantification of oPMNs to complement periodontal evaluation, particularly at early stages where traditional markers are limited. While preliminary, the framework demonstrates feasibility and potential for screening, longitudinal monitoring, and timely clinical referral. Future work will validate BioSA in larger, diverse cohorts, integrate complementary techniques such as flow cytometry and Pap cytology, and explore mobile and fluidic systems for point-of-care and at-home monitoring, representing a promising step toward scalable saliva-based diagnostics for PD.

The transparent CS coating enables brightfield imaging and integration with a DNN for automated oPMN detection and quantitative assessment of OIL. The primary contribution of this work is the rapid, low-cost quantification of oPMNs as a measure of OIL, providing early, non-invasive insight into inflammation and risk stratification rather than serving as a standalone diagnostic tool. Periodontal diagnosis, as defined by the 2017 World Periodontal Workshop [7], is multifactorial, involving clinical attachment loss, probing depth, bleeding on probing, RBL, and patient history. These parameters are less sensitive at early stages, such as periodontal health and gingivitis, where tissue damage has not occurred, and conventional markers may be absent or inconsistent. In contrast, oPMNs reflect the host inflammatory response to microbial burden and may rise before structural damage, making OIL a complementary biomarker for screening, monitoring, and preliminary “statistical” PD staging. BioSA enables rapid, reproducible quantification of oPMNs using brightfield imaging and AI, with preliminary results supporting its role in augmenting, not replacing, established diagnostic frameworks. Repeatability and quantitative agreement were confirmed in 90 additional experiments, showing high precision and close correspondence with conventional oPMN counting. By exploiting intrinsic neutrophil adhesion without fluorescent labels or complex procedures, BioSA offers a simple, scalable approach. Combined with AI-assisted analysis, the platform provides rapid and reproducible OIL assessment. In summary, BioSA delivers low-cost, objective quantification of oPMNs to complement periodontal evaluation, particularly at early stages where traditional markers are limited. While preliminary, the framework demonstrates feasibility and potential for screening, longitudinal monitoring, and timely clinical referral. Future work will validate BioSA in larger, diverse cohorts, integrate complementary techniques like flow cytometry and Pap cytology, and explore mobile and fluidic systems for point-of-care and at-home monitoring, representing a promising step toward scalable saliva-based diagnostics for PD.

## Data Availability

All data generated or analyzed during this study are included in this published article and its Supplementary Information Files. All codes used in this paper will be made available upon request by reviewers.

## Supplementary Materials

The following supporting information can be downloaded at: https://www.mdpi.com/article/10.3390/biomimetics11090673/s1.
