# Supplementary material for: Mechanistic Characterization of Biologically Inspired Oral Neutrophil Isolation for AI-Assisted Oral Inflammatory Load Assessment
Source: Biomimetics (Basel). 2026 Sep 18;11(9):673. doi: 10.3390/biomimetics11090673 (PMC13604207; doi:10.3390/biomimetics11090673)
Supplement: Supplementary file 1 [file biomimetics-11-00673-s001.zip › biomimetics-4516008 SI-BioSA.pdf]

# *Supporting Information*

# AI-Assisted, Biologically Inspired Oral Neutrophil Quantification for Digital Periodontal Diagnostics

*Fatemeh Soheili[1,2], Mahdi S.M.H. Daneshvar[1], Navid Mohaghegh[1], John V. L. Nguyen[1,2,3], Abbas Panahi[1], Mahdi Amrollahi Biouki[1], Yasaman Tahernezhad[1], Chunxiang Sun[4], Saghi Forouhi[5,1], Michael Glogauer[4], Ebrahim Ghafar-Zadeh[1,2]**

[1]Biologically Inspired Sensors and Actuators Laboratory (BioSA), Department of Electrical Engineering and Computer Science, Lassonde School of Engineering, York University, 4700 Keele Street, Toronto, ON, M3J 1P3, Canada
[2]Department of Biology, York University, 4700 Keele Street, Toronto, ON, M3J 1P3, Canada
[3]Department of Biomedical Engineering, McGill University, 3775 University Street, Montreal, QC, H3A 2B4, Canada
[4]Faculty of Dentistry, University of Toronto, Toronto, ON, M5G 1G6, Canada
[5]Division of Electronics and Computer Engineering (ELDA), Department of Electrical Engineering (ISY), Linköping University, 581 83 Linköping, Sweden
*Corresponding authors: Ebrahim Ghafar-Zadeh (egz@yorku.ca)

# Background and Benchmark Comparison

Figure S1 illustrates the biological and diagnostic context motivating early detection of periodontal disease (PD). After routine cleaning, a pellicle layer rapidly forms and matures into plaque and eventually calculus, initiating gingival inflammation within days to weeks. Without intervention, this local inflammatory process deepens into periodontal pocketing, attachment loss, and bone resorption, ultimately leading to tooth loss. Importantly, PD is increasingly recognized as a systemic condition: chronic periodontal inflammation enables oral bacteria and inflammatory mediators to enter the bloodstream, contributing to cardiovascular disease, diabetes, rheumatoid arthritis, adverse pregnancy outcomes, oral cancer, neurodegeneration, and osteoporosis. Despite these risks, current clinical tools—including visual assessment, bleeding on probing, clinical attachment loss, and radiographic bone loss—identify PD only after irreversible structural damage has occurred. Molecular biosensing approaches have begun addressing this gap. Recent examples include aptamer-based electrochemical sensors for ODAM capable of nanomolar detection, sandwich-type aptamer configurations enabling patient stratification, and salivary biomarkers such as MMP-8, oxidative stress molecules, CRP, and IL-1β. Although promising, molecular markers often suffer from biological variability, limited specificity, and binary outputs that poorly reflect disease dynamics. Because neutrophils are direct effectors of oral immunity, oral polymorphonuclear neutrophils (oPMNs) represent a biologically integrated alternative that reflects real-time inflammatory activity. Salivary oPMN levels correlate with PD severity across the gingivitis–periodontitis spectrum, but systemic conditions and medical treatments can influence their abundance. These limitations underscore the need for a platform that captures oPMN biology directly and quantitatively, motivating our cellular biomarker–based strategy for early detection and dynamic severity scoring of periodontal disease. This figure was partially generated using AI-

assisted illustration tools, while all layout, content, and scientific interpretation were created and verified by the authors.

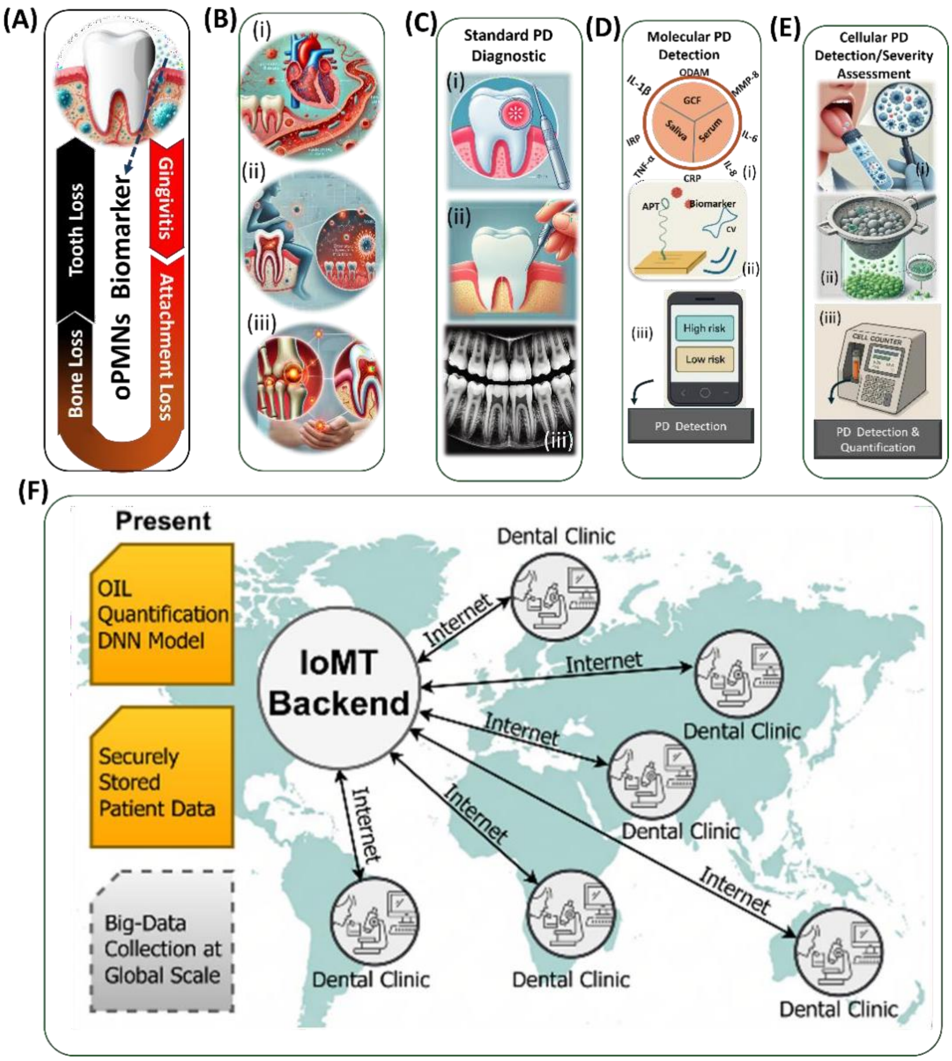


**Figure S1.** Overview of PD Progression, Associated Risks, and Diagnostic Methods. (A) PD Progression: The disease begins with pellicle formation shortly after oral cleaning and progresses through plaque accumulation and tartar deposition. This leads to gingivitis, clinical attachment loss, and eventual alveolar bone degradation. Diagnostic stages highlight increasing periodontal pocket depth as the disease advances. (B) Associated Systemic Conditions: PD is linked to several systemic diseases, including cardiovascular disease, adverse pregnancy outcomes (e.g., preterm birth), diabetes, rheumatoid arthritis, respiratory disease, and Alzheimer's disease. (C) Diagnostic Techniques: Clinical methods for PD assessment include (i) bleeding on probing (BOP), (ii) clinical attachment (CA) measurements to evaluate pocket depth, and (iii) radiographic imaging (X-ray) to detect bone loss. (D) Molecular Biomarkers: Biomarker-based diagnostics utilize saliva or gingival crevicular fluid (GCF) to measure (i) C-reactive protein (CRP), (ii) odontogenic ameloblast-associated protein (ODAM), (iii) matrix metalloproteinase-8 (MMP-8), and (iv) interleukin-1β (IL-1β). (E) Oral Neutrophils as Biomarkers: Oral polymorphonuclear neutrophils (oPMNs), markers of local inflammation, are (i) collected from the oral cavity, (ii) filtered to isolate the cells, and (iii) quantified for diagnostic insight. (F) The proposed IoMT framework for remote diagnostic and big-data collection.

As summarized in Table S1, the BioSA method offers substantial operational advantages compared with established laboratory-based approaches, such as HEMO fluorescence microscopy, Pap cytology, and flow cytometry. BioSA requires only a brightfield microscope

and basic laboratory supplies, resulting in a low setup cost and a rapid 15–20-minute workflow, which includes saliva deposition, brief settling, PBS washing, and brightfield imaging, followed by automated AI-based oPMN counting. In contrast, HEMO, Pap cytology, and flow cytometry require more complex instrumentation—fluorescence microscopes with filter sets, cytology staining stations, or high-cost flow cytometers—along with multistep staining or labeling procedures that extend total processing times to 45–90 minutes, 60–120 minutes, and 2–3 hours, respectively. The required level of expertise follows the same pattern: BioSA can be performed by non-specialized personnel, whereas HEMO demands microscopy experience, Pap cytology requires skilled cytotechnologists, and flow cytometry depends on highly trained operators familiar with panel design, instrument calibration, and gating. Cost per test is correspondingly lower for BioSA (<$1), using minimal disposables, while comparator methods require fluorescent antibodies, fixatives, dyes, or specialized cytometry consumables that significantly increase per-sample costs. These differences directly impact scalability and portability: BioSA offers high-throughput potential and can be deployed across a wide range of clinical settings, whereas the equipment and expertise demand of HEMO, Pap cytology, and flow cytometry restrict their practicality to specialized laboratories. Despite its operational simplicity, BioSA maintains high accuracy for periodontal disease staging, comparable to fluorescence-based neutrophil quantification, while offering far superior accessibility and efficiency for real-world screening.

**Table S1.** Condensed comparison of BioSA with standard oPMN analysis methods

| **Metric** | **BioSA** | **HEMO (Fluorescence)** | **Pap Cytology** | **Flow Cytometry** |
|---|---|---|---|---|
| **Total Time** | 15–20 min | 45–90 min | 60–120 min | 2–3 h |
| **Expertise Needed** | Low | Moderate | High | Very high |
| **Setup Equipment** | Brightfield microscope; basic labware | Fluorescence microscope; filter sets; staining station | Cytology staining unit; brightfield microscope | Flow cytometer; computer; QC beads |
| **Setup Cost** | **Low** | **High** | **High** | **Very High** |
| **Cost per Test** | **< $1** | Moderate | Moderate | High |
| **oPMN Counting** | Automated (AI based) | Manual | Manual | Automated |
| **Accuracy (PD)** | High | High | High | Very high |
| **Scalability** | High | Moderate | Moderate | Very low |
| **Portability** | Excellent | Limited | Limited | Poor |

# DNN based oPMN counting: Training and Assessment

## YOLOv4 model

The YOLOv4 architecture consists of three main blocks: Backbone, Neck, and Head. The Backbone is responsible for feature extraction and typically uses CSPDarknet[1] or CSPResNeXt[2]. Due to the superior performance of CSPDarknet[1], as indicated in the original YOLOv4 paper, we selected it as our backbone network in this study. As seen in Figure S2, the Neck combines features from the Backbone and prepares them for detection, mixing information from the contracting (spatially rich) and expanding (contextually rich) blocks of the network, and then propagating the combined features to the detection part of the model. The Head enables one-stage object detection for dense prediction, providing a vector that includes class label, prediction confidence score, and bounding box information (x, y, width, height).

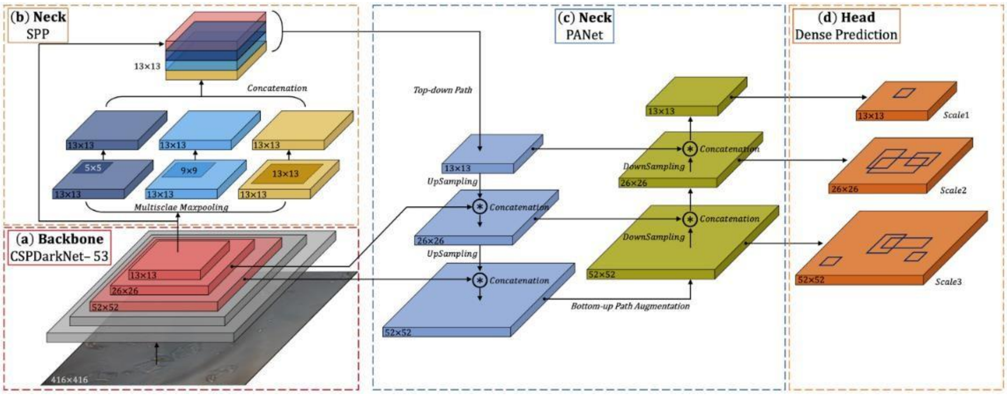


**Figure S2.**YOLOv4 overall structure. (a) Backbone: CSPDarkNet-53. (b-c) Neck: SPP and PANet, (d) Head: Dense prediction from three different scales.

Compared to precedent YOLO architectures, YOLOv4 also applies two model training modification strategies namely bag of freebies (BoF) and bag of specials (BoS), that can improve the overall performance even having a modest amount of training dataset. BoF is composed of strategies such as data augmentation, loss function modification, and hyperparameter tuning that in general, do not influence the algorithm testing FPS and the only cost is during the training phase; thus, it is an off-line procedure. In contrast, BoS strategies improve performance by model architecture redesign, though this has a minor impact on the runtime of the algorithm.

## Image Dataset

After sample preparation, 226 saliva microscopic images are captured using brightfield microscope with an x20 objective. These images were annotated by biologist expert using the graphical image annotation tool in Ref[3]. The cohort of microscopic images with the size of 1,080×1,920 comprises 4,617 oPMNs, in which train, validation, and test datasets were selected randomly. In each raw microscopic image within the dataset, oPMNs were confined with an appropriate bounding box and the results were saved in YOLO format. In each microscopic image, Neutrophils are the objects, while the remaining structures including

epithelial cells are considered as the background.

## Optimization and Quantification Methods

In order to optimize the performance of our algorithm, we used transfer-learning and utilized the weights of a pre-trained model on MS COCO dataset [4] for initializing our model's backbone weights. Compared to the randomly initialized network, it is hypothesized to achieve higher precision. The precision, sensitivity and $F_1\ score$ are the used metrics to evaluate our AI solution, which are defined in the equations S(1) to S(3).

$$Sensitivity = \frac{TP}{TP + FN} \quad \text{S(1)}$$

$$Presicion = \frac{TP}{TP + FP} \quad \text{S(2)}$$

$$F_1\ score\ = 2 \times \frac{Sensitivity \times Presicion}{Sensitivity + Presicion} \quad \text{S(3)}$$

Where $TP =$ true positive, $FP =$ false positive, and $FN =$ false negative. The sensitivity metric in equation S(1) provides a quantitative measurement of detected neutrophils, while precision gives a measure of the detection quality. A higher level of $TP$s is associated with higher sensitivity, whereas a smaller number of $FP$s determines the precision of the algorithm. $F_1\ score$ is the harmonic mean of sensitivity and precision and gives a measure of accuracy and the balance between two metrics. Also, we just preserve the class label probabilities above 50% to limit the number of false positives. Overall, there is a trade-off between increasing the $TP$ rate and decreasing the number of $FP$s. Based on our experience, 50% confidence holds the balance between these two parameters.

## Front-end and back-end software

The backend hardware consists of multiple servers for different purposes. The AI model is deployed on a server equipped with a 64-core Intel Xeon processor, 128 GB of RAM and a Nvidia A40 GUP for fast inference. On average, apart from loading the model into GPU's memory, running the AI model for each image takes 88ms on average. Another server, the gateway server, hosted in an AWS EC2 t2 micro instance, is used to facilitate access to the inference server. The gateway server runs an OpenVPN server, which lets the clients and inference server be mutually accessible in a secure way. End users can access the functionality of the model, i.e. running the Neutrophil detection on arbitrary images, through the front-end application. Currently, we have developed two front-end applications. The first is a Windows-based GUI application that enables users to connect to the gateway server via OpenVPN and submit tasks to the AI inference server in user-friendly fashion. To maximize the accessibility of the system and enable usage of it on other devices and operating systems, a second front-end application is made available as a web app. The web app has similar features to the desktop GUI application. The users can log into the application, upload microscope images of the saliva specimen and submit the batch to the AI server to detect.

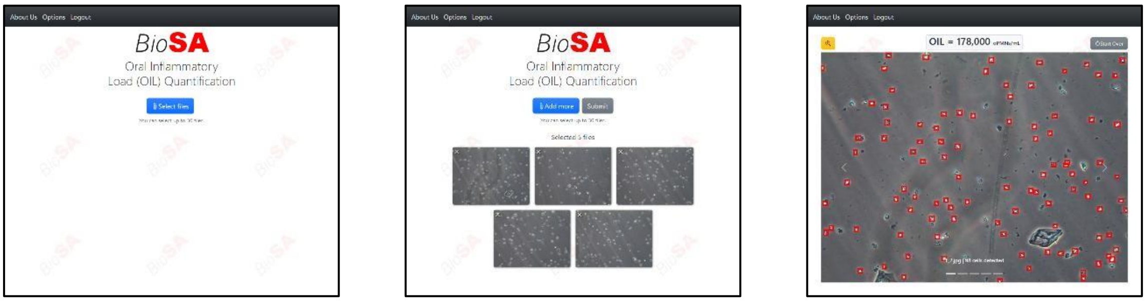


(a) (b) (c)

**Figure S3.** The user interface of the web application, before image selection, after image selection and the final results consisting of the OIL quantification and detected cells for each individual image.

## Deep Learning Detection and counting

In this work, the described algorithm in the last section was trained in over 9,000 iterations and the best-performing model on the validation dataset was used to compute the test accuracy. The results of the algorithm on the test dataset including sensitivity, precision and $F_1$ *score* are shown in Table S3.

To reveal the functionality of the implemented deep learning framework, as examples, five different images were chosen from the test set (Figure S4a). Then, the algorithm's results, together with the ground truth, were shown in Figure S4c and Figure S4b, respectively. Figure S4c depicts the detected oPMN after applying the deep learning algorithm with a confidence threshold of 50%. False positives and false negatives are shown in red and yellow, respectively.

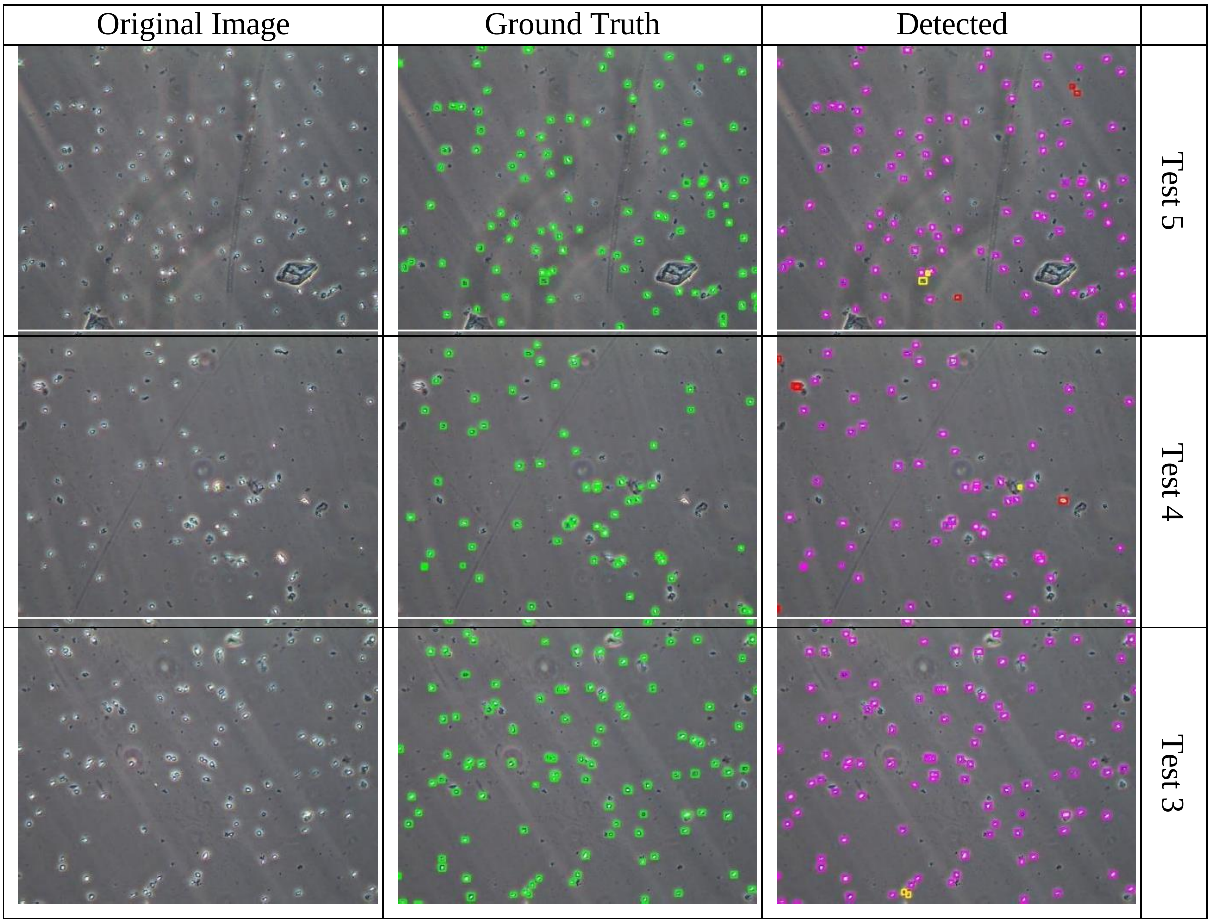

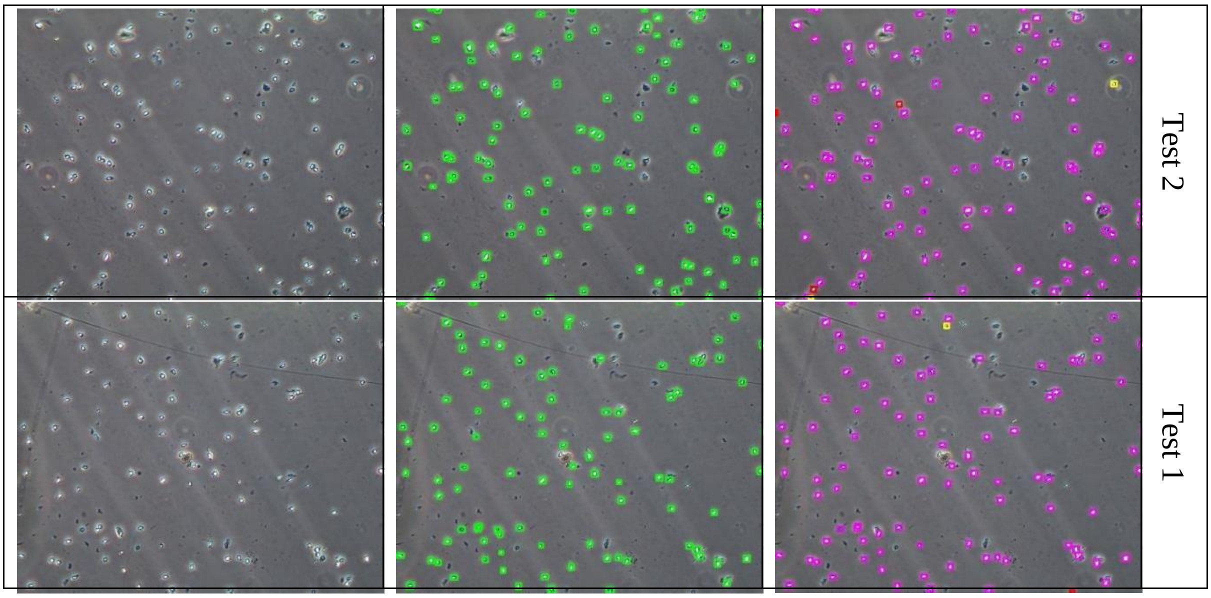


**Figure S4.** Five examples showing the performance of AI. Column (a) shows the original images, column (b) shows the ground truth, and column (c) shows the cells as detected by the AI model.

Figure S5 shows the histogram of confidence values for the five images. As seen in the figures the majority of detections have confidence above 90% with 96% of detections above 50%.

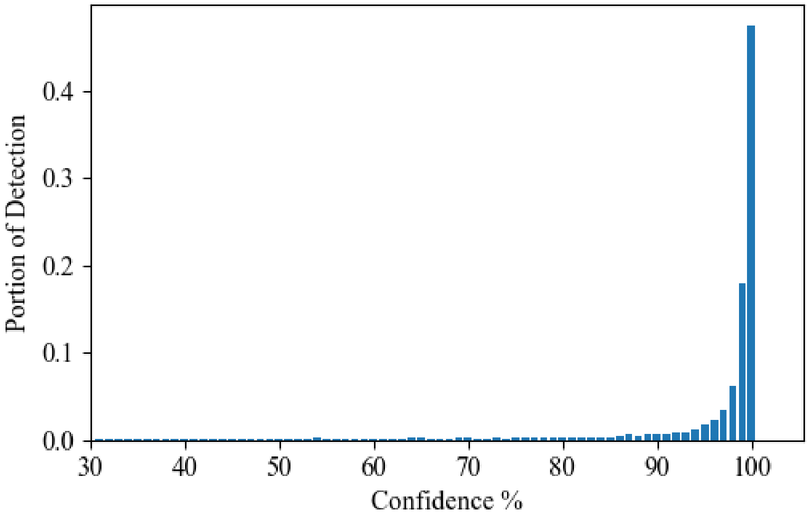


**Figure S5.** Confidence histogram of the detection on the five example images shown in Figure S4.

In the assessment of these five images, several types of errors were observed. These are summarized in Table S2. The first type of error occurs in some of the areas where two cells are close to each other and share a border; these cells were detected as a single cell, which counts as a false negative, as the other cell is not detected. The second type of error originates from some objects closely resembling neutrophils being detected as such, resulting in a false positive error. The last type of error is not detecting (false negative) the neutrophils that exhibit uncommon morphological features. These include old and decaying cells. In order to make the

AI models stronger to deal with these kinds of errors and mitigate their effect on the performance, we need to create a larger training dataset with more annotated cells in all these error-prone types and retrain the model. The retrained model can correctly identify these cells as it sees many examples of such cells in the training set.

**Table S2.** Types of errors observed and example image regions

| Error | Examples |
|---|---|
| Twin, neighboring cells Detected as a single cell (FN) | 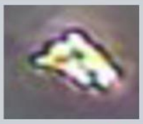 |
| Non-neutrophil, Detected wrongly as neutrophil (FP) | 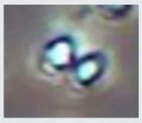 |
| Old, decaying, exploding cell, Not detected (FN) | 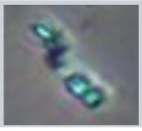 |

**Table S3.** Performance metrics for the assessment of five example images

| | |
|---|---|
| Total detections | 505 |
| False positives | 17 |
| False negatives | 9 |
| True positives | 488 |
| Sensitivity | 0.98 |
| Precision | 0.97 |
| $F_1$ score | 0.97 |

## Role of CD markers on Hydrophilicity of Oral Neutrophils

**Table S4.** Amino Acids and their Kyte–Doolittle Value.

| Amino Acid | 1-Letter Code | Kyte–Doolittle Value |
|---|---|---|
| Isoleucine | I | +4.5 |
| Valine | V | +4.2 |
| Leucine | L | +3.8 |
| Phenylalanine | F | +2.8 |
| Cysteine | C | +2.5 |
| Methionine | M | +1.9 |
| Alanine | A | +1.8 |
| Glycine | G | −0.4 |
| Threonine | T | −0.7 |
| Serine | S | −0.8 |
| Tryptophan | W | −0.9 |
| Tyrosine | Y | −1.3 |
| Proline | P | −1.6 |
| Histidine | H | −3.2 |
| Glutamate | E | −3.5 |
| Aspartate | D | −3.5 |
| Asparagine | N | −3.5 |

| | | |
|---|---|---|
| Glutamine | Q | −3.5 |

| Amino Acid | 1-Letter Code | Kyte–Doolittle Value |
|---|---|---|
| Lysine | K | −3.9 |
| Arginine | R | −4.5 |

**Table S5.** CD markers of Oral Neutrophil.

| CD Marker | UniProt ID | Extracellular Length (aa) |
|---|---|---|
| CD11b (ITGAM) | P11215 | ~1–1080 |
| CD14 | P08571 | 1–375 |
| CD16 (FCGR3B) | P08631 | 1–208 |
| CD45 (PTPRC) | P08575 | ~1–600 |
| CD66b (CEACAM8) | P31996 | ~1–301 |
| CD62L (SELL) | P14151 | 1–370 |
| CD63 | P08962 | ~1–219 |
| CD64 (FCGR1A) | P12314 | ~1–310 |
| CD66a (CEACAM1) | P13688 | ~1–310 |
| CD47 | Q08722 | 1–303 |
| CD88 (C5aR1) | P21730 | ~1–350 |
| CD89 (FCAR) | P15987 | ~1–272 |
| CD274 (PD-L1) | Q9NZQ7 | ~1–239 |
| CD64 (FcγRI) | P12314 | ~1–310 |
| CD206 (MRC1) | Q13654 | ~1–713 |

# COMSOL Simulation

The simulations were performed in the fluid simulation module of COMSOL. The pipette and a model of a circular petri dish were created in the CAD design area of COMSO. Then, the Navier stocks equations were solved within a meshed domain with the finite element method. The solved equations are provided by S(4) to S(5). The boundary conditions include the wall condition for the petri dish's surface and the pressure output for the surrounding walls of the petri dish. The governing equations used in the simulations are as follows:

Continuity Equation (Conservation of Mass):

$$\nabla . u = 0 \qquad \text{S(4)}$$

where u is the velocity vector of the fluid. This equation ensures mass conservation within the fluid domain. The flow simulation has been done by solving the Navier-Stokes Equation (Conservation of Momentum) in a meshed domain; the equation is as follows:

$$\rho \left(\frac{\partial u}{\partial t} + u . \nabla u\right) = -\nabla p + \mu \nabla^2 u + F \qquad \text{S(5)}$$

The fluid density is dynamic viscosity, p is the pressure, F represents any external forces applied to the fluid (e.g., gravitational forces if included). The boundary condition used in the simulation considers the wall condition on the Petri-dish Surface and the pipette wall by applying a no-slip boundary condition on the Petri-dish surface, enforcing $u$ = 0 on these boundaries. Also, the pressure outlet condition on the surrounding walls of the petri dish is considered.

## COMSOL Simulation details for calculation of falling velocity

The following equation (Navier-Stokes and continuity) has been solved in COMSOL for the cell falling in a fluid in cylindrical coordinates.

$$\rho \frac{\partial u}{\partial t} - \nabla.\mu(\nabla u + (\nabla u)^T) + \rho u.\nabla u + \nabla p = F$$
$$\nabla.u = 0 \qquad \text{S(6)}$$

In equation S(3), the $\rho$ denotes the density (kg/m$^3$), u is the velocity (m/s), $\mu$ is the viscosity (Ns/m$^2$), and the p is the pressure (Pa). The model uses the accelerating reference system of the cell. This means that the volume force density F is considered by

$$F_r = 0,\ F_z = -\rho\ (a + g) \qquad \text{S(7)}$$

where an (m/s$^2$) is the acceleration of the cell and $g$ = 9.81, m/s$^2$ is the acceleration due to gravity. The ODE that defines the force balance is:

$$m\ddot{x} = F_g + F_z \qquad \text{S(8)}$$

where m (kg) is the mass of the cell, x (m) is the position of the particle, Fg (N) is the gravitational force, and Fz is the z-component of the force that the water exerts on the cell. The gravitational force is given by:

$$F_g = -\rho_{cell} V_{cell} g \qquad \text{S(9)}$$

where $V_{cell}$ (m$^3$) is the volume of the cell and $\rho$ cell is the density of the cell. The following relation calculates the force that the fluid exerts on the cell:

$$F_z = 2\pi \int r\boldsymbol{z}.[-pI + \mu(\nabla\boldsymbol{u} + (\nabla\boldsymbol{u})^T)]\boldsymbol{n}dS \qquad \text{S(10)}$$

where $r$ (m) is the radial coordinate, z is the unit vector in the z direction, and n is the normal vector on the cell's surface.

In this simulation, we have considered the viscosity of the fluid as 5-20 cP (as shown in Figure S6 and used that in our prediction of the falling velocity of cells in the fluid. According to [5], saliva can hold a density of 1.002-1.012 g/ml, and neutrophils, this can have a density of 1.077-1.083 g/ml. Here, we have considered values of 1.002 g/ml and 1.077 g/ml for neutrophil/epithelial and saliva, respectively.

## Shear stress along the centerline (diameter)

In an attempt to understand the effect of the pipette's vertical position on the shear stress generated on the surface of the petri dish, using the same methodology of simulation and boundary conditions , the simulation was performed for various vertical distances of pipette from the surface of petri-dish and calculated along the diameter of the petri-dish as shown in Figure S6. The results show that pipetting is essential in the introduced mechanism, and its position affects the shear stress generated due to the flow of the pipette. While this simulation study is not meant to provide quantitative shear stress exerted on the cells in the sample, it aims to provide an understanding of the method used in the investigation for separating neutrophil cells from epithelial cells using a pipette and petri dish.

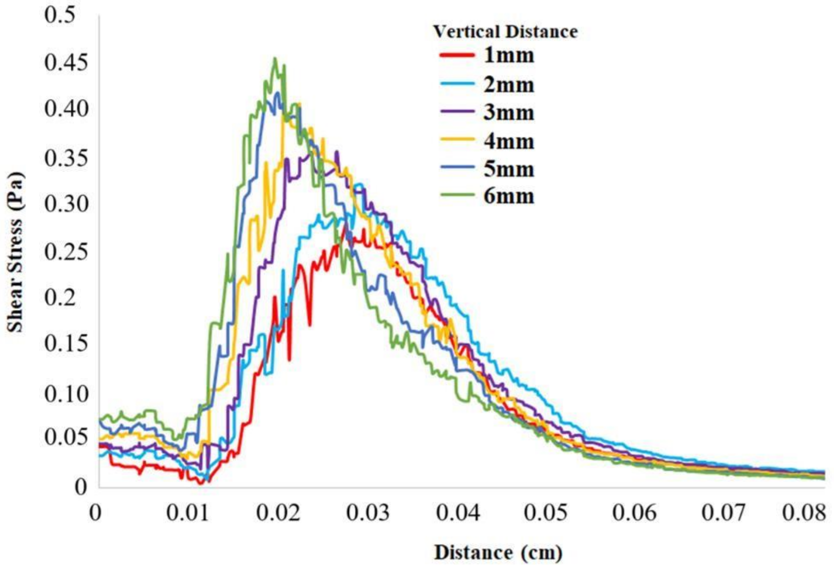


**Figure S6.** Shear stress along the centerline (diameter) of the Petri dish at different pipette heights to demonstrate the effect of the pipette positioning on the shear stress generated on the petri dish's surface. This figure illustrates that increasing the pipette height produces variations in shear stress distribution, providing insight into achieving optimal shear stress levels across the dish for even cell distribution for future engineering designs incorporating this methodology.

In another attempt, the effect of the neutrophil cell size on the falling velocity is simulated, as shown in Figure S7. The simulation was to show that in a constant shape, the falling velocity increases as the mass of cells increases due to an increase in their volume for a constant density. According to Figure S7, as the cell radiation increases, it experiences a higher falling velocity, considering other parameters constant in the simulation.

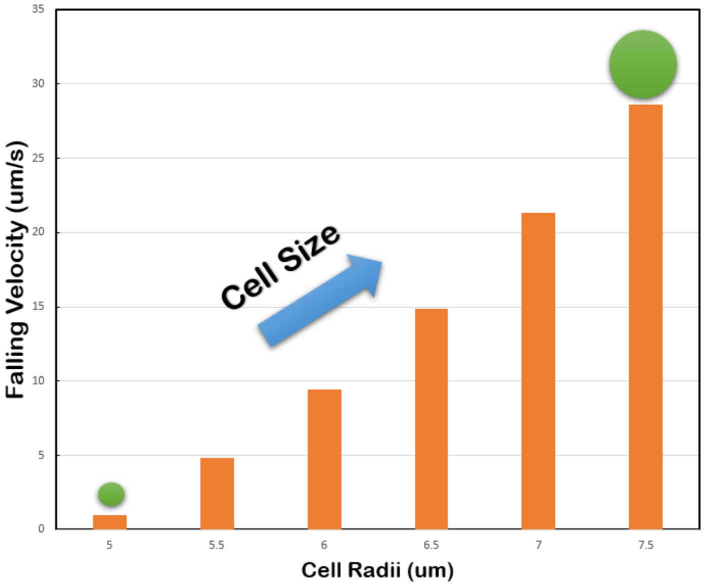


**Figure S7.** The falling velocity of the cell with a sphericity of 1 (complete circular) shows the effect of the cell size on the falling velocity.

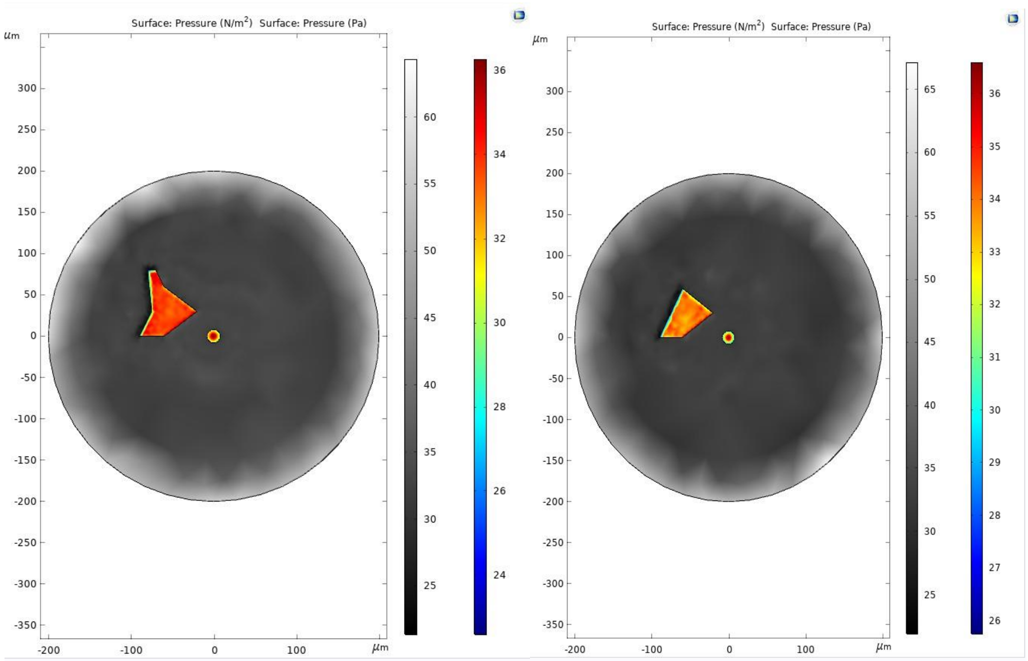


**Figure S8.** Pressure gradient around neutrophil cell under suction. This figure highlights how cells with larger surface areas interact with pressure gradients, with epithelial cells experiencing greater forces than smaller neutrophils. The counters of pressure acting on a neutrophil (in the center) and epithelial cell (right image: one epithelial cell; left: aggregated epithelial cell with higher surface area). Epithelial cells have been considered in two states, single and aggregated with another cell, which directly increases its active surface area.

# Statistical Analysis

## Characterization: Enhanced Hydrophilicity

In this section, we assess the effects of cornstarch (CSt) on increasing the adhesion of oPMNs to the surface of a petri-dish. Additionally, we study the optimal values for CSt concentration, waiting time (T1), volume of PBS, and the concentration of PBS in a mixture with water.

### *Collected data*

Table S6 shows the data collected from ten groups of experiments, where each group involved three samples taken from healthy human subjects. In this table, the number of oPMNs in each microscopic image before normalization by multiplying by 21,800 is displayed. Similarly, Table S7 were generated when the surface of the glass petri-dish was coated with CSt and introduced to the saliva sample.

**Table S6.** Collected data associated with experiments performed on the surface without CSt before washing.

| *Group* | Before | After | B-STDEV | A-STDEV |
|---|---|---|---|---|
| *G1* | 32.3 | 21.4 | 3.380083 | 4.829832 |
| *G2* | 32.1 | 20.2 | 3.453579 | 4.626718 |
| *G3* | 29.8 | 19.1 | 3.587552 | 4.445078 |
| *G4* | 28.1 | 14.7 | 3.114345 | 3.8666 |
| *G5* | 30.8 | 19.7 | 2.920873 | 3.920148 |
| *Gc* | 31.2 | 21.4 | 2.976983 | 3.149643 |
| *G7* | 27 | 15 | 3.343804 | 3.515564 |
| *G8* | 31.6 | 23 | 2.776137 | 3.497314 |
| *G2* | 28.8 | 16.9 | 2.619842 | 3.629574 |
| *G10* | 32.1 | 19.4 | 2.599785 | 2.622988 |

**Table S7.** Collected data associated with experiments performed on the surface with CSt after washing.

| | Before | After | B-STDEV | A-STDEV |
|---|---|---|---|---|
| *G1* | 28.7 | 22.4 | 3.40098 | 5.337498 |

| | | | | |
|---|---|---|---|---|
| *G2* | 30.7 | 26.8 | 4.547282 | 6.069962 |
| *G3* | 29.5 | 25.1 | 4.006938 | 7.109462 |
| *G4* | 33.2 | 29.6 | 4.184628 | 5.660781 |
| *G5* | 28.6 | 23.2 | 4.971027 | 5.95912 |
| *Gc* | 31.5 | 27.7 | 2.915476 | 7.394442 |
| *G7* | 32.2 | 28.1 | 3.119829 | 4.532598 |
| *G8* | 29.4 | 22.5 | 3.627059 | 5.147815 |
| *G2* | 31.9 | 28 | 2.806738 | 3.399346 |
| *G10* | 30.5 | 27.3 | 2.838231 | 5.292552 |

To strengthen the statistical interpretation of Figure 3, oPMN retention was analyzed using paired before–after measurements across surface conditions. Reduction rate and retention rate were calculated for each replicate. Normality (Shapiro–Wilk) and variance homogeneity (Levene's test) were assessed prior to group comparison. Because these assumptions were not fully satisfied, both parametric (ANOVA/Welch) and non-parametric (Kruskal–Wallis) approaches were used. The global comparison demonstrated a statistically significant effect of surface condition on oPMN retention. Corrected pairwise comparisons showed that CS-coated glass achieved significantly lower reduction rates compared to most alternative surfaces, supporting its role as the optimal hydrophilic substrate for BioSA.

Figure S9 visually illustrates these findings through paired before–after measurements for each surface condition. Each line represents a single replicate, showing the change in oPMN count following the washing process. While all surfaces exhibit a reduction in cell count, the magnitude of loss differs substantially across conditions. Cornstarch-treated glass consistently shows smaller decreases, indicating higher oPMN retention, whereas non-treated and polystyrene-based surfaces display larger drops in cell counts. The relatively tight clustering of paired differences for CS-coated glass further supports its stable and reproducible retention performance compared to other surfaces.

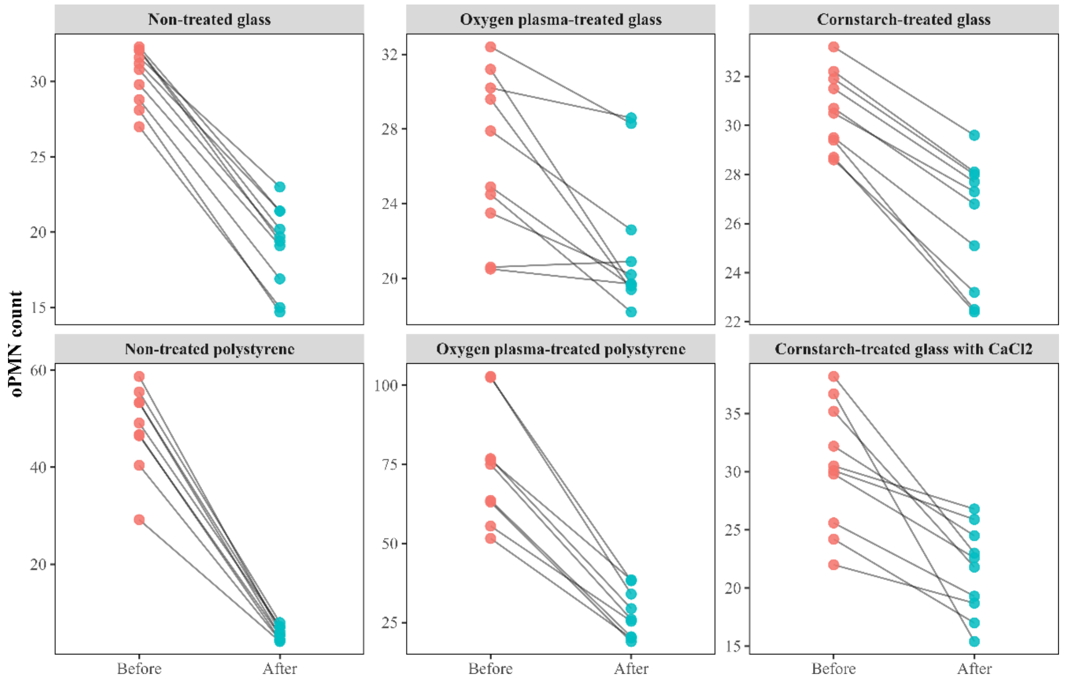


**Figure S9.** Surface-dependent oPMN retention before and after isolation. Paired oPMN counts for individual replicates before and after washing across different surface conditions. Lines indicate within-sample changes. Cornstarch-treated glass shows the highest retention, whereas non-treated and polystyrene surfaces exhibit greater cell loss. Detailed statistical analyses are provided in the Supplementary Information.

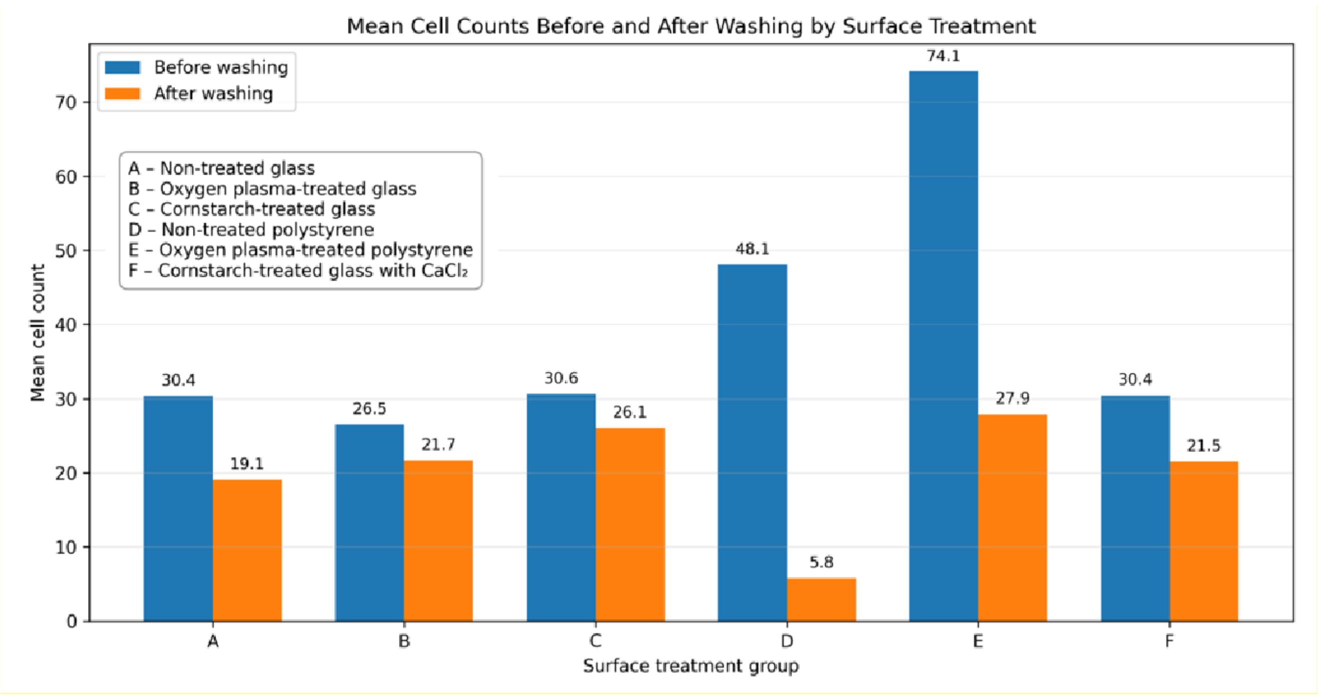

**Figure S10.** Mean cell counts before and after washing across different surface treatments. Blue and orange bars represent counts before and after washing, respectively, for (A) non-treated glass, (B) oxygen plasma-treated glass, (C) cornstarch-treated glass, (D) non-treated polystyrene, (E) oxygen plasma-treated polystyrene, and (F) cornstarch-treated glass with $CaCl_2$. Numbers above the bars indicate the mean cell count for each condition.

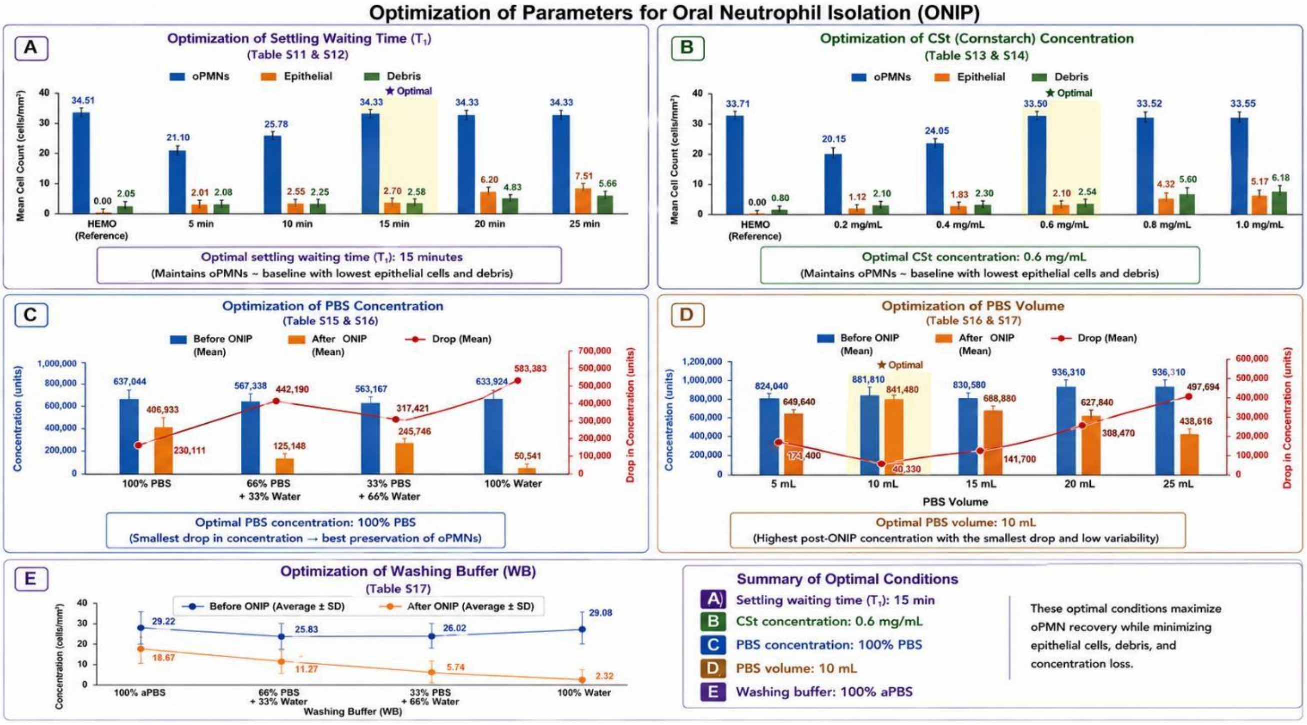


**Figure S11.** Optimization of parameters for oral neutrophil isolation (ONIP). (A) Effect of settling waiting time ($T_1$) on oral polymorphonuclear neutrophil (oPMN), epithelial cell, and debris counts. (B) Effect of cornstarch (CSt) concentration on the same counts. (C) Effect of phosphate-buffered saline (PBS) concentration on oPMN concentrations before and after ONIP and the corresponding concentration decrease. (D) Effect of PBS volume on these measurements. (E) Effect of washing buffer composition on oPMN counts before and after ONIP; error bars represent standard deviations. The selected optimal conditions were a settling time of 15 min, CSt concentration of 0.6 mg/mL, 100% PBS, PBS volume of 10 mL, and 100% PBS as the washing buffer, balancing oPMN preservation with reduced epithelial cell and debris contamination.

*TableS7.1 Raw_Clean*

| GroupID | Before | After | Source | ReductionRate | RetentionRate | Absolute Change | Letter | Surface |
|---|---|---|---|---|---|---|---|---|
| **G1** | 32.3 | 21.4 | Glass Bare | 33.74613 | 66.25387 | -10.9 | A | Non-treated glass |
| **G2** | 32.1 | 20.2 | Glass Bare | 37.0716511 | 62.9283489 | -11.9 | A | Non-treated glass |
| **G3** | 29.8 | 19.1 | Glass Bare | 35.9060403 | 64.0939597 | -10.7 | A | Non-treated glass |
| **G4** | 28.1 | 14.7 | Glass Bare | 47.6868327 | 52.3131673 | -13.4 | A | Non-treated glass |
| **G5** | 30.8 | 19.7 | Glass Bare | 36.038961 | 63.961039 | -11.1 | A | Non-treated glass |

| | | | | | | | | |
|---|---|---|---|---|---|---|---|---|
| **G6** | 31.2 | 21.4 | Glass Bare | 31.4102564 | 68.5897436 | -9.8 | A | Non-treated glass |
| **G7** | 27 | 15 | Glass Bare | 44.4444444 | 55.5555556 | -12 | A | Non-treated glass |
| **G8** | 31.6 | 23 | Glass Bare | 27.2151899 | 72.7848101 | -8.6 | A | Non-treated glass |
| **G9** | 28.8 | 16.9 | Glass Bare | 41.3194444 | 58.6805556 | -11.9 | A | Non-treated glass |
| **G10** | 32.1 | 19.4 | Glass Bare | 39.5638629 | 60.4361371 | -12.7 | A | Non-treated glass |
| **G1** | 29.8 | 22.6 | CaCl2 | 24.1610738 | 75.8389262 | -7.2 | F | Cornstarch-treated glass with CaCl2 |
| **G2** | 36.7 | 15.4 | CaCl2 | 58.0381471 | 41.9618529 | -21.3 | F | Cornstarch-treated glass with CaCl2 |
| **G3** | 38.2 | 23 | CaCl2 | 39.7905759 | 60.2094241 | -15.2 | F | Cornstarch-treated glass with CaCl2 |
| **G4** | 32.2 | 24.5 | CaCl2 | 23.9130435 | 76.0869565 | -7.7 | F | Cornstarch-treated glass with CaCl2 |
| **G5** | 25.6 | 19.3 | CaCl2 | 24.609375 | 75.390625 | -6.3 | F | Cornstarch-treated glass with CaCl2 |
| **G6** | 35.2 | 21.8 | CaCl2 | 38.0681818 | 61.9318182 | -13.4 | F | Cornstarch-treated glass with CaCl2 |
| **G7** | 24.2 | 17 | CaCl2 | 29.7520661 | 70.2479339 | -7.2 | F | Cornstarch-treated glass with CaCl2 |
| **G8** | 30.1 | 25.9 | CaCl2 | 13.9534884 | 86.0465116 | -4.2 | F | Cornstarch-treated glass with CaCl2 |

| | | | | | | | | |
|---|---|---|---|---|---|---|---|---|
| **G9** | 22 | 18.7 | CaCl2 | 15 | 85 | -3.3 | F | Cornstarch-treated glass with CaCl2 |
| **G10** | 30.5 | 26.8 | CaCl2 | 12.1311475 | 87.8688525 | -3.7 | F | Cornstarch-treated glass with CaCl2 |
| **G1** | 23.5 | 20.2 | Oxygen Plasma | 14.0425532 | 85.9574468 | -3.3 | B | Oxygen plasma-treated glass |
| **G2** | 27.9 | 22.6 | Oxygen Plasma | 18.9964158 | 81.0035842 | -5.3 | B | Oxygen plasma-treated glass |
| **G3** | 29.6 | 19.4 | Oxygen Plasma | 34.4594595 | 65.5405405 | -10.2 | B | Oxygen plasma-treated glass |
| **G4** | 20.5 | 19.7 | Oxygen Plasma | 3.90243902 | 96.097561 | -0.8 | B | Oxygen plasma-treated glass |
| **G5** | 20.6 | 20.9 | Oxygen Plasma | -1.4563107 | 101.456311 | 0.3 | B | Oxygen plasma-treated glass |
| **G6** | 24.5 | 18.2 | Oxygen Plasma | 25.7142857 | 74.2857143 | -6.3 | B | Oxygen plasma-treated glass |
| **G7** | 24.9 | 19.6 | Oxygen Plasma | 21.2851406 | 78.7148594 | -5.3 | B | Oxygen plasma-treated glass |
| **G8** | 32.4 | 28.3 | Oxygen Plasma | 12.654321 | 87.345679 | -4.1 | B | Oxygen plasma-treated glass |
| **G9** | 31.2 | 19.7 | Oxygen Plasma | 36.8589744 | 63.1410256 | -11.5 | B | Oxygen plasma-treated glass |
| **G10** | 30.2 | 28.6 | Oxygen Plasma | 5.29801325 | 94.7019868 | -1.6 | B | Oxygen plasma-treated glass |

| | | | | | | | | |
|---|---|---|---|---|---|---|---|---|
| **G1** | 28.7 | 22.4 | With CS | 21.9512195 | 78.0487805 | -6.3 | C | Cornstarch-treated glass |
| **G2** | 30.7 | 26.8 | With CS | 12.7035831 | 87.2964169 | -3.9 | C | Cornstarch-treated glass |
| **G3** | 29.5 | 25.1 | With CS | 14.9152542 | 85.0847458 | -4.4 | C | Cornstarch-treated glass |
| **G4** | 33.2 | 29.6 | With CS | 10.8433735 | 89.1566265 | -3.6 | C | Cornstarch-treated glass |
| **G5** | 28.6 | 23.2 | With CS | 18.8811189 | 81.1188811 | -5.4 | C | Cornstarch-treated glass |
| **G6** | 31.5 | 27.7 | With CS | 12.0634921 | 87.9365079 | -3.8 | C | Cornstarch-treated glass |
| **G7** | 32.2 | 28.1 | With CS | 12.7329193 | 87.2670807 | -4.1 | C | Cornstarch-treated glass |
| **G8** | 29.4 | 22.5 | With CS | 23.4693878 | 76.5306122 | -6.9 | C | Cornstarch-treated glass |
| **G9** | 31.9 | 28 | With CS | 12.2257053 | 87.7742947 | -3.9 | C | Cornstarch-treated glass |
| **G10** | 30.5 | 27.3 | With CS | 10.4918033 | 89.5081967 | -3.2 | C | Cornstarch-treated glass |
| **G1** | 102.8 | 34 | Oxygen Polystyrene | 66.92607 | 33.07393 | -68.8 | E | Oxygen plasma-treated polystyrene |
| **G2** | 51.6 | 20.1 | Oxygen Polystyrene | 61.0465116 | 38.9534884 | -31.5 | E | Oxygen plasma-treated polystyrene |
| **G3** | 63.1 | 19 | Oxygen Polystyrene | 69.889065 | 30.110935 | -44.1 | E | Oxygen plasma-treated polystyrene |
| **G4** | 102.5 | 38.2 | Oxygen Polystyrene | 62.7317073 | 37.2682927 | -64.3 | E | Oxygen plasma-treated polystyrene |

| | | | | | | | | |
|---|---|---|---|---|---|---|---|---|
| **G5** | 76.3 | 38.5 | Oxygen Polystyrene | 49.5412844 | 50.4587156 | -37.8 | E | Oxygen plasma-treated polystyrene |
| **G6** | 63.6 | 20.5 | Oxygen Polystyrene | 67.7672956 | 32.2327044 | -43.1 | E | Oxygen plasma-treated polystyrene |
| **G7** | 76.8 | 29.4 | Oxygen Polystyrene | 61.71875 | 38.28125 | -47.4 | E | Oxygen plasma-treated polystyrene |
| **G8** | 75 | 26 | Oxygen Polystyrene | 65.3333333 | 34.6666667 | -49 | E | Oxygen plasma-treated polystyrene |
| **G9** | 55.5 | 25.4 | Oxygen Polystyrene | 54.2342342 | 45.7657658 | -30.1 | E | Oxygen plasma-treated polystyrene |
| **G1** | 58.7 | 6.1 | Non-O2 Polystyrene | 89.6081772 | 10.3918228 | -52.6 | D | Non-treated polystyrene |
| **G2** | 53.3 | 7 | Non-O2 Polystyrene | 86.8667917 | 13.1332083 | -46.3 | D | Non-treated polystyrene |
| **G3** | 49.1 | 7.2 | Non-O2 Polystyrene | 85.3360489 | 14.6639511 | -41.9 | D | Non-treated polystyrene |
| **G4** | 29.2 | 4.1 | Non-O2 Polystyrene | 85.9589041 | 14.0410959 | -25.1 | D | Non-treated polystyrene |
| **G5** | 53.3 | 5.4 | Non-O2 Polystyrene | 89.8686679 | 10.1313321 | -47.9 | D | Non-treated polystyrene |
| **G6** | 46.7 | 4.5 | Non-O2 Polystyrene | 90.3640257 | 9.6359743 | -42.2 | D | Non-treated polystyrene |

| | | | | | | | | |
|---|---|---|---|---|---|---|---|---|
| **G7** | 40.4 | 4.4 | Non-O2 Polystyrene | 89.1089109 | 10.8910891 | -36 | D | Non-treated polystyrene |
| **G8** | 55.5 | 8 | Non-O2 Polystyrene | 85.5855856 | 14.4144144 | -47.5 | D | Non-treated polystyrene |
| **G9** | 46.5 | 5.4 | Non-O2 Polystyrene | 88.3870968 | 11.6129032 | -41.1 | D | Non-treated polystyrene |

*TableS7.2 Summary*

| Letter | Surface | Source | n | mean_before | sd_before | mean_after | sd_after |
|---|---|---|---|---|---|---|---|
| **A** | Non-treated glass | Glass Bare | 10 | 30.38 | 1.86773779 | 19.08 | 2.7579 |
| **B** | Oxygen plasma-treated glass | Oxygen Plasma | 10 | 26.53 | 4.32718795 | 21.72 | 3.7240 |
| **C** | Cornstarch-treated glass | With CS | 10 | 30.62 | 1.56687942 | 26.07 | 2.5906 |
| **D** | Non-treated polystyrene | Non-O2 Polystyrene | 9 | 48.0777778 | 8.96671313 | 5.78888889 | 1.3779 |
| **E** | Oxygen plasma-treated polystyrene | Oxygen Polystyrene | 9 | 74.1333333 | 18.4615817 | 27.9 | 7.6041 |
| **F** | Cornstarch-treated glass with CaCl2 | CaCl2 | 10 | 30.45 | 5.36744301 | 21.5 | 3.8026 |

*TableS7.3 Normality test*

| Letter | Surface | n | shapiro_p |
|---|---|---|---|
| **A** | Non-treated glass | 10 | 0.99683065 |
| **B** | Oxygen plasma-treated glass | 10 | 0.81608969 |
| **C** | Cornstarch-treated glass | 10 | 0.03673546 |
| **D** | Non-treated polystyrene | 9 | 0.20708896 |
| **E** | Oxygen plasma-treated polystyrene | 9 | 0.3824396 |
| **F** | Cornstarch-treated glass with CaCl2 | 10 | 0.27140694 |

*TableS7.4 _Equality Variance test*

| Df | F value | Pr(>F) |
|---|---|---|
| **5** | 3.69147548 | 0.00620447 |
| **52** | | |

*TableS7.5 Figure3_Anova test*

| Term | df | sumsq | meansq | statistic | p.value |
|---|---|---|---|---|---|
| **Letter** | 5 | 38021.195 | 7604.239 | 94.3300639 | 7.6719E-25 |
| **Residuals** | 52 | 4191.88127 | 80.6131013 | | |

*TableS7.6 Kruskal-Wallis test*

| statistic | p.value | parameter | method |
|---|---|---|---|
| **46.9183324** | 5.9035E-09 | 5 | Kruskal-Wallis rank sum test |

*TableS7.7 key test*

| Comparison | A vs C | B vs C | D vs C | E vs C | F vs C |
|---|---|---|---|---|---|
| **group_1** | A | B | D | E | F |
| **group_2** | C | C | C | C | C |
| **mean_group_1** | 37.4402813 | 17.1755292 | 87.8982454 | 62.1320279 | 27.9417099 |
| **mean_group_2** | 15.0277857 | 15.0277857 | 15.0277857 | 15.0277857 | 15.0277857 |
| **mean_difference_group_2_minus_group_1** | -22.412496 | -2.1477435 | -72.87046 | -47.104242 | -12.913924 |
| **cohens_d_group_2_minus_group_1** | -4.1223297 | -0.2227514 | -19.77791 | -8.3024223 | -1.2232216 |
| **wilcox_p** | 0.00018267 | 0.67758496 | 0.0002797 | 0.0002797 | 0.00728456 |
| **welch_p** | 5.1966E-08 | 0.62789841 | 4.8872E-15 | 3.6799E-11 | 0.01945041 |
| **wilcox_p_holm** | 0.00091336 | 0.67758496 | 0.0011188 | 0.0011188 | 0.01456911 |
| **welch_p_holm** | 1.559E-07 | 0.62789841 | 2.4436E-14 | 1.472E-10 | 0.03890081 |

*TableS7.8 Pairwise_Wilcox test*

| | A | B | C | D | E |
|---|---|---|---|---|---|
| **B** | 0.00853125 | | | | |

| | | | | | |
|---|---|---|---|---|---|
| **C** | 0.00274008 | 0.67758496 | | | |
| **D** | 0.0039158 | 0.0039158 | 0.0039158 | | |
| **E** | 0.0039158 | 0.0039158 | 0.0039158 | 0.0039158 | |
| **F** | 0.16170767 | 0.24244901 | 0.02913823 | 0.0039158 | 0.0039158 |

*TableS7.9 Pairwise_Welch*

| | **A** | **B** | **C** | **D** | **E** |
|---|---|---|---|---|---|
| **B** | 0.00293738 | | | | |
| **C** | 5.7162E-07 | 0.62789841 | | | |
| **D** | 6.0438E-10 | 2.1365E-07 | 7.3308E-14 | | |
| **E** | 1.9678E-06 | 1.4735E-06 | 5.1518E-10 | 6.363E-06 | |
| **F** | 0.22427418 | 0.22427418 | 0.07780162 | 1.9678E-06 | 6.9972E-05 |

## Optimization

The performance of the proposed method is influenced by several factors, including the hydrophilicity of the CSt coating on the surface, waiting time allowing the settlement of oPMNs which also results in the settling of epithelial cells in the long term, the volume of PBS

added to separate the epithelial cells from the surface, and the concentration of PBS in a mixture with water that can affect the breaking of weak affinity of epithelial cells and their removal during the ONIP. In this section, the optimal values for the concentration of CSt affecting hydrophilicity, the optimal waiting time, the PBS concentration, and the volume of PBS in the proposed isolation process are obtained experimentally, and the statistical analysis is applied and discussed accordingly.

## *Settling waiting time*

Table S8 presents the collected data for various settling times (T1) ranging from 5 to 25 minutes.

**Table S8**. Collected data for optimization of waiting time (T1).

| *Measurement* | HEMO | 5min | 10min | 15min | 20min | 25min |
|---|---|---|---|---|---|---|
| *oPMNs* | 34.51 | 21.1 | 25.78 | 34.33 | 34.33 | 34.33 |
| *Epithelial* | 0 | 2.01 | 2.55 | 2.7 | 6.2 | 7.51 |
| *Debri* | 2.05 | 2.08 | 2.25 | 2.58 | 4.83 | 5.66 |
| *O-SD* | 8.07 | 6.82 | 11.78 | 6.71 | 13.58 | 6.59 |
| *E-SD* | 0 | 1.69 | 1.38 | 1.97 | 2.99 | 3.09 |
| *D-SD* | 0.79 | 1.71 | 1.87 | 1.9 | 1.96 | 2.06 |

Table S9 compares different filtration times (5 min, 10 min, 15 min, 20 min, and 25 min) in terms of their effectiveness in maintaining oPMNs levels while minimizing the presence of epithelial cells and debris. Among the times tested, the 15-minute filtration time emerges as the optimal choice. It not only preserves oPMNs levels close to the baseline but also results in the lowest counts of epithelial cells and debris. Moreover, the measurements at 15 minutes show the least variability, indicating consistent and reliable results.

**Table S9**. Summarizing the optimization data for time optimization.

| Condition | Standard Filter | 5min | 10min | 15min | 20min | 25min |
|---|---|---|---|---|---|---|
| Mean_oPMNs | 34.51 | 21.1 | 25.78 | 34.33 | 34.33 | 34.33 |
| Mean_Epithelial | 0 | 2.01 | 2.55 | 2.7 | 6.2 | 7.51 |
| Mean_Debri | 2.05 | 2.08 | 2.25 | 2.58 | 4.83 | 5.66 |
| SD_oPMNs | 8.07 | 6.82 | 11.78 | 6.71 | 13.58 | 6.59 |
| SD_Epithelial | 0 | 1.69 | 1.38 | 1.97 | 2.99 | 3.09 |
| SD_Debri | 0.79 | 1.71 | 1.87 | 1.9 | 1.96 | 2.06 |
| Drop_oPMNs | 0 | -13.41 | -8.73 | -0.18 | -0.18 | -0.18 |
| Drop_Epithelial | 0 | 2.01 | 2.55 | 2.7 | 6.2 | 7.51 |
| Drop_Debri | 0 | 0.03 | 0.2 | 0.53 | 2.78 | 3.61 |

**SD, Mean, Drop terms represent standard deviation, mean value, and decreased values.

## *Concentration of HCM*

Table S10 presents the collected data for various HCM (cornstarch) concentrations, ranging from 0.2 mg/mL to 1 mg/mL.

**Table S10**. Collected data for optimization of concentration of HCM.

| CS mg(CS)/ml | HEMO | 0.2 | 0.4 | 0.6 | 0.8 | 1 |
|---|---|---|---|---|---|---|
| oPMNs | 33.71 | 20.15 | 24.05 | 33.5 | 33.52 | 33.55 |
| Epithelial | 0 | 1.12 | 1.83 | 2.1 | 4.32 | 5.17 |
| Debri | 0.8 | 2.1 | 2.3 | 2.54 | 5.6 | 6.18 |
| O-SD | 6.63009 | 5.03958 | 6.692297 | 5.947136 | 7.19649 | 6.8133 |
| E-SD | 0 | 1.67332 | 1.297771 | 1.68333 | 1.7252 | 2.56443 3 |
| D-SD | 0.60697 7 | 1.486784 | 1.76516 | 1.431782 | 1.49032 | 2.05899 8 |

Table S11 compares different concentrations of CSt (0.6 mg/ml, 0.8 mg/ml, and 1.0 mg/ml) in maintaining oPMNs levels, epithelial cells, and debris, using the proposed BioSA method against a standard Filter. The results show that while all three concentrations effectively maintain oPMNs levels close to the baseline value of 33.71, the concentration of 0.6 mg/ml is optimal. It not only maintains oPMNs levels with minimal deviation but also results in the lowest levels of epithelial cells and debris. Additionally, the standard deviations for oPMNs, epithelial cells, and debris at 0.6 mg/ml indicate consistent and reliable results, making this concentration the most balanced and effective option for achieving the desired outcomes.

**Table S11**. Summarizing the optimization data for CS optimization.

| *Condition* | *Standard Filter* | *0.2 mg/ml* | *0.4 mg/ml* | *0.6 mg/ml* | *0.8 mg/ml* | *1.0 mg/ml* |
|---|---|---|---|---|---|---|
| *Mean_oPMNs* | 33.71 | 20.15 | 24.05 | 33.5 | 33.52 | 33.55 |
| *Mean_Epithelial* | 0 | 1.12 | 1.83 | 2.1 | 4.32 | 5.17 |
| *Mean_Debri* | 0.8 | 2.1 | 2.3 | 2.54 | 5.6 | 3.18 |
| *SD_oPMNs* | 6.63009 | 5.03958 | 6.692297 | 5.947136 | 7.19649 | 6.8133 |
| *SD_Epithelial* | 0 | 1.67332 | 1.297771 | 1.68333 | 1.7252 | 2.564433 |
| *SD_Debri* | 0.606977 | 1.486784 | 1.76516 | 1.431782 | 1.49032 | 2.058998 |
| *Drop_oPMNs* | 0 | -13.56 | -9.66 | -0.21 | -0.19 | -0.16 |
| *Drop_Epithelial* | 0 | 1.12 | 1.83 | 2.1 | 4.32 | 5.17 |
| *Drop_Debri* | 0 | 1.3 | 1.5 | 1.74 | 4.8 | 5.38 |

## *PBS concentration*

Table S12 presents the collected data for various PBS concentration ranging from 0 to 100%.

**Table S12**. Summarizing the optimization data for PBS concentrations.

| *Concentration* | *Before* | *After* | *Drop* | *SD_Before* | *SD_After* |
|---|---|---|---|---|---|
| *PBS* | 637044.4 | 406933.3 | 230111.1 | 57931.13 | 69699.42 |
| *PBS_66%* | 567338.3 | 125148.1 | 442190.1 | 54391.07 | 53095.86 |
| *PBS_33%* | 563166.7 | 245745.5 | 317421.2 | 70940.6 | 120825.6 |
| *Water* | 633923.5 | 50540.6 | 583382.9 | 81685.94 | 26311.24 |

Table S13 presents the numerical analysis of concentration changes before and after ONIP, focusing on the reduction in oPMN levels under different conditions. The condition with 100% PBS showed the smallest drop in concentration (230,111.1 units), indicating it is the most

effective in preserving the initial material during ONIP. In contrast, using water resulted in the largest drop (583,382.9 units), highlighting significant material loss. Intermediate conditions with varying PBS concentrations in water showed reductions between these two extremes. The findings suggest that 100% PBS is optimal for minimizing concentration loss during ONIP.

**Table S13**. Summarizing the optimization data for PBS volume.

| *Volume* | *5mL* | *10mL* | *15mL* | *20mL* | *25mL* |
|---|---|---|---|---|---|
| *Mean_Before_ONIP* | 824040 | 881810 | 830580 | 936310 | 936310 |
| *Mean_After_ONIP* | 649640 | 841480 | 688880 | 627840 | 438616 |
| *Mean_Drop* | 174400 | 40330 | 141700 | 308470 | 497694 |
| *SD_Before_ONIP* | 183581 | 198815 | 195612 | 106639 | 143387 |
| *SD_After_ONIP* | 96578 | 144093 | 57769 | 88713 | 86421 |

## *PBS volume*

Table S13 presents the collected data for various PBS volumes ranging from 5 mL to 25 mL.

Table S14 presents numerical specifics and reinforces the findings visually. For instance, the 10 ml volume showed the smallest average decrease (40330), whereas the 20 ml volume exhibited a notable average drop (308470). Post-ONIP standard deviations also decrease, underscoring the process's effectiveness in stabilizing concentration levels. Based on these observations, the optimal volume condition appears to be 10 mL, where the post-ONIP concentration remains relatively high at 841480 with minimal drop. This suggests that ONIP at this volume minimally disrupts and maintains a higher concentration, making it the optimal condition for preserving higher concentrations after ONIP while reducing variability.

**Table S14**. Collected data for optimization of washing buffer (WB).

| *PBS Concentration* | Average | Standard Deviation |
|---|---|---|
| *100% aPBS Before* | 29.22222 | 2.657391 |
| *100% aPBS After* | 18.66667 | 3.197221 |
| *cc% PBS, 33% W Before* | 25.83333 | 3.254156 |
| *cc% PBS, 33% W After* | 11.27273 | 5.51769 |
| *33% PBS, cc% W Before* | 26.02469 | 2.495003 |
| *33% PBS, cc% W After* | 5.740741 | 2.43559 |
| *100% Water Before* | 29.07906 | 3.747062 |
| *100% Water After* | 2.318376 | 1.206938 |

## Clinical data

Table S15 shows the oPMN measurement results from Twenty-one clinical samples using the proposed BioSA method.

**Table S15.** Collected clinical data.

| *Info* | Hemo | BIOSA | B-STDEV |
|---|---|---|---|
| *Healthy_1* | 102460 | 89380 | 128027.4 |

| | | | |
|---|---|---|---|
| *Healthy_2* | 111180 | 95920 | 117315.6 |
| *Healthy_3* | 580000 | 540000 | 134118.7 |
| *Healthy_4* | 550000 | 570000 | 144867.5 |
| *Healthy_5* | 600000 | 610000 | 62615.73 |
| *Healthy_c* | 650000 | 670000 | 188793.5 |
| *Gingivitis_1* | 900000 | 890000 | 95244.2 |
| *Gingivitis_2* | 920000 | 920000 | 89910.14 |
| *Gingivitis_3* | 950000 | 950000 | 64705.98 |
| *Gingivitis_4* | 950000 | 950000 | 169550.2 |
| *Mild periodontal disease_5* | 1500000 | 1500000 | 207775.8 |
| *Mild periodontal disease_c* | 2000000 | 1850000 | 161025.1 |
| *Mild periodontal disease_7* | 2000000 | 2000000 | 141431.4 |
| *Mild periodontal disease_8* | 3000000 | 2900000 | 182391.9 |
| *Moderate PD_2* | 3100000 | 3100000 | 120295.7 |
| *Moderate PD_10* | 3500000 | 3490000 | 143433.4 |
| *Moderate PD_11* | 3900000 | 3700000 | 147597.4 |
| *Moderate PD_12* | 4200000 | 4300000 | 185863.4 |
| *Severe_13* | 5700000 | 5800000 | 113276.1 |
| *Severe_14* | 7500000 | 7500000 | 155515.2 |
| *Severe_15* | 9000000 | 9000000 | 71276.33 |

### *Linearity*

The comparative results between BioSA and HEMO demonstrate a high level of agreement across multiple statistical measures. The slope of the regression line (1.02) and its 95%

confidence interval (0.98–1.07) indicate that the two methods increase at nearly the same rate, suggesting proportional consistency between measurements. The intercept is small relative to the overall measurement range, further indicating that the two assays align closely across low and high values. The model's coefficient of determination ($R^2$ = 0.99) confirms that BioSA explains almost all of the variation observed in HEMO results, reflecting excellent linear correspondence between the two techniques.

Error-based metrics also show strong concordance. The absolute and relative errors (RMSE = $2.36\times10^5$; RMSE% = 9.45%; MAE = $2.06\times10^5$; MAPE = 12.4%) remain small in the context of the large oPMN counts, demonstrating that differences between methods are minor in magnitude and consistent across samples. Correlation analyses reinforce this pattern, with Pearson's r (0.995) and Spearman's ρ (0.993) both approaching 1.0, indicating near-perfect alignment in both absolute values and sample ranking.

The Bland–Altman analysis provides a sample-level assessment of agreement between the two measurement methods. The bias ($-1.93\times10^5$) represents the average difference between BioSA and HEMO, indicating that BioSA measurements are only slightly lower on average—a small offset relative to the multi-million oPMN range. The limits of agreement ($-6.81\times10^5$ to $2.94\times10^5$) define the interval within which most paired differences fall. Because this interval is narrow compared with the scale of the measurements, it suggests that the two methods remain consistently close across individual samples. Together, these values confirm that BioSA and HEMO provide closely matching results with minimal systematic disagreement.

Overall, these results demonstrate strong linear concordance, small and consistent measurement differences, and excellent sample-level agreement between BioSA and HEMO.

**Table S16.** Agreement and linearity metrics between BioSA and Control (HEMO) measurements.

| METRIC | VALUE | EXPLANATION |
|---|---|---|
| **SLOPE** | 1.02 | Indicates near-proportional scaling between BioSA and HEMO measurements. |
| **95% CI** | 0.98 – 1.07 | Confidence interval for slope. |
| **INTERCEPT** | $1.36\times10^5$ | Small offset between measurement methods; close to zero on the scale of measurements. |
| **95% CI** | $-2.4\times10^4$ – $3.0\times10^5$ | Confidence interval for intercept. |
| **$R^2$** | 0.99 | Shows excellent linear fit and shared variance (99%). |
| **RMSE** | $2.36\times10^5$ | Absolute prediction error in oPMN counts. |

| | | |
|---|---|---|
| **RMSE (%)** | 9.45% | Relative error normalized to mean HEMO values. |
| **MAE** | $2.06×10^5$ | Average absolute difference between paired measurements. |
| **MAPE (%)** | 12.4% | Average percentage deviation between methods. |
| **PEARSON R** | 0.995 | Very strong linear correlation. |
| **SPEARMAN Ρ** | 0.993 | Strong monotonic (rank-based) concordance. |
| **BA BIAS** | $–1.93×10^5$ | Mean difference between methods; small relative to the range. |
| **BA LOA (LOWER)** | $–6.81×10^5$ | Lower 95% limit of agreement. |
| **BA LOA (UPPER)** | $2.94×10^5$ | Upper 95% limit of agreement. |

**Table S17.** Summary statistics for log linear regression model.

| *Term* | *Estimate* | *Std. Error* | *p-value* | *Statistic Value* |
|---|---|---|---|---|
| *(Intercept)* | 4.20528 | 0.31810 | <2e-16 | |
| *BioSA* | 0.71599 | 0.02248 | <2e-16 | |
| *RMSE* | | | | 0.3857 |
| | *R-squared* | | | 0.8299 |
| *F-statistic* | | | 2.2e-16 | |

The Bland–Altman analysis across the 30-day repeatability study shows tighter limits of agreement due to controlled experimental conditions, whereas the clinical dataset exhibits wider variability reflecting biological heterogeneity. Despite this, both analyses demonstrate consistent bias direction and no proportional error, supporting robust agreement between BioSA and HEMO.

## *Categorization: PD prediction*

The *k*-means clustering algorithm involves the following steps mathematically and has been performed in R (package). The first step is the initialization: start by choosing $k$=5 (where k is the number of clusters we want to create). In practice, these can be chosen randomly, or they might be the means of randomly selected subsets of the data. The next step is the cluster assignment: every data point $x_i$ is assigned to the nearest cluster by minimizing the distance between xi and the cluster centroid $\mu_j$. Typically, Euclidean distance is used $d(x_i, \mu_j) = (\sum_{n=1}^{N=21}(x_{i,n} - \mu_{j,n})^2)^{0.5}$ where $N$ is the number of features. For each cluster, the centroid is

recalculated as the mean of all points assigned to that cluster $\mu j = (\sum_{x_i \in S_j} x_i)/|S_j|$, Here, $S_j$ is the set of all points assigned to the $j$-th cluster. Steps 2 and 3 are iterated and repeated until the centroids do not change significantly or the changes are within a specified tolerance level, indicating convergence. In the next step, evaluation should be performed. The quality of clustering can often be evaluated using the Within-Cluster Sum of Squares (WCSS), which the k-means algorithm tries to minimize in order to determine the belonging of each data point, $WCSS = \sum_{j=1}^{k=5} \sum_{x_i \in S_j} (x_i - \mu_j)^2$ .

Table S18 shows the results of a k-means clustering analysis, where samples have been grouped into five distinct categories based on their similarities. Each set of samples (e.g., 1-6, 7-11) is assigned to a specific category, indicating that those samples share similar characteristics according to the clustering method.

**Table S18.** K-means methods.

| ***Samples*** | ***1-6*** | ***7-11*** | ***12-15*** | ***16-19*** | ***20-21*** |
|---|---|---|---|---|---|
| ***Category*** | ***5*** | ***3*** | ***4*** | ***2*** | ***1*** |

## Accuracy of Proposed BioSA Method versus Control

Figure 7 in the main text compares BioSA and HEMO measurements across 30 test days. Because each BioSA value has a corresponding HEMO value from the same test day, the analysis was reframed as a paired method-comparison. For each day, the paired difference

between BioSA and HEMO was calculated, and agreement was summarized using the mean difference, 95% confidence interval, MAE, RMSE, correlation/regression, and Bland–Altman limits of agreement. This provides a clearer assessment of repeatability and method agreement than relying only on a fixed-effects model. The results showed that BioSA followed the same temporal pattern as HEMO but slightly underestimated HEMO on average. The Bland–Altman analysis was used to quantify this systematic offset and the expected range of paired differences. Overall, these results support BioSA as a reproducible method for oPMN quantification while transparently reporting the magnitude of method-level bias.

*Table S18.1 Paired BioSA–HEMO data for 30 days*

| Time | BioSA | HEMO | Difference_BioSA_minus_HEMO | Difference_HEMO_minus_BioSA | Mean_Methods | Absolute_Error | Percent_Error |
|---|---|---|---|---|---|---|---|
| 1 | 660540 | 707410 | -46870 | 46870 | 683975 | 46870 | 6.62557781 |
| 2 | 655090 | 716130 | -61040 | 61040 | 685610 | 61040 | 8.52359209 |
| 3 | 728120 | 706320 | 21800 | -21800 | 717220 | 21800 | 3.08641975 |
| 4 | 656180 | 744470 | -88290 | 88290 | 700325 | 88290 | 11.8594436 |
| 5 | 724850 | 756460 | -31610 | 31610 | 740655 | 31610 | 4.17867435 |
| 6 | 671440 | 717220 | -45780 | 45780 | 694330 | 45780 | 6.38297872 |
| 7 | 662720 | 723760 | -61040 | 61040 | 693240 | 61040 | 8.43373494 |
| 8 | 711770 | 766270 | -54500 | 54500 | 739020 | 54500 | 7.11237553 |
| 9 | 677980 | 753190 | -75210 | 75210 | 715585 | 75210 | 9.98552822 |
| 10 | 654000 | 785890 | -131890 | 131890 | 719945 | 131890 | 16.7822469 |
| 11 | 707410 | 722670 | -15260 | 15260 | 715040 | 15260 | 2.11161388 |
| 12 | 620210 | 700870 | -80660 | 80660 | 660540 | 80660 | 11.5085537 |
| 13 | 718310 | 747740 | -29430 | 29430 | 733025 | 29430 | 3.93586006 |
| 14 | 664900 | 773900 | -109000 | 109000 | 719400 | 109000 | 14.084507 |
| 15 | 658360 | 705230 | -46870 | 46870 | 681795 | 46870 | 6.64605873 |
| 16 | 712860 | 744470 | -31610 | 31610 | 728665 | 31610 | 4.24597365 |
| 17 | 721580 | 744470 | -22890 | 22890 | 733025 | 22890 | 3.07467057 |

| | | | | | | | |
|---|---|---|---|---|---|---|---|
| **18** | 728120 | 722670 | 5450 | -5450 | 725395 | 5450 | 0.75414781 |
| **19** | 681250 | 680160 | 1090 | -1090 | 680705 | 1090 | 0.16025641 |
| **20** | 725940 | 765180 | -39240 | 39240 | 745560 | 39240 | 5.12820513 |
| **21** | 722670 | 796790 | -74120 | 74120 | 759730 | 74120 | 9.30232558 |
| **22** | 677980 | 671440 | 6540 | -6540 | 674710 | 6540 | 0.97402597 |
| **23** | 657270 | 684520 | -27250 | 27250 | 670895 | 27250 | 3.98089172 |
| **24** | 698690 | 723760 | -25070 | 25070 | 711225 | 25070 | 3.46385542 |
| **25** | 614760 | 693240 | -78480 | 78480 | 654000 | 78480 | 11.3207547 |
| **26** | 636560 | 607130 | 29430 | -29430 | 621845 | 29430 | 4.84739677 |
| **27** | 652910 | 623480 | 29430 | -29430 | 638195 | 29430 | 4.72027972 |
| **28** | 724850 | 704140 | 20710 | -20710 | 714495 | 20710 | 2.94117647 |
| **29** | 761910 | 772810 | -10900 | 10900 | 767360 | 10900 | 1.41043724 |
| **30** | 656180 | 706320 | -50140 | 50140 | 681250 | 50140 | 7.09876543 |

*Table S18.2 Agreement summary: mean difference, 95% CI, MAE, RMSE, Pearson correlation*

| n_paired_days | 30 |
|---|---|
| **mean_BioSA** | 684847 |
| **mean_HEMO** | 722270.333 |
| **mean_difference_BioSA_minus_HEMO** | -37423.333 |
| **sd_difference** | 40538.5552 |
| **bland_altman_lower_loa** | -116878.9 |
| **bland_altman_upper_loa** | 42032.2349 |
| **paired_t_p** | 2.169E-05 |
| **paired_t_ci_low** | -52560.679 |
| **paired_t_ci_high** | -22285.988 |
| **paired_wilcox_p** | 0.0001102 |
| **pearson_r** | 0.50005927 |
| **pearson_p** | 0.00489404 |
| **spearman_rho** | 0.46274643 |
| **spearman_p** | 0.01002659 |
| **r_squared** | 0.25005928 |
| **slope_BioSA_on_HEMO** | 0.42803196 |
| **slope_ci_low** | 0.14108247 |

| | |
|---|---|
| **slope_ci_high** | 0.71498144 |
| **intercept** | 375692.217 |
| **MAE** | 45053.3333 |
| **RMSE** | 54672.6731 |
| **MAPE_percent** | 6.15601093 |

*Table S18.3 Bland–Altman bias and limits of agreement*

| term | estimate | std.error | statistic | p.value | conf.low | conf.high |
|---|---|---|---|---|---|---|
| **(Intercept)** | 665263.333 | 20604.3297 | 32.2875504 | 2.7703E-24 | 623122.748 | 707403.919 |
| **MethodControl** | 37423.3333 | 7401.29371 | 5.05632323 | 2.169E-05 | 22285.988 | 52560.6786 |
| **Day2** | 1635 | 28665.0873 | 0.05703803 | 0.9549061 | -56991.686 | 60261.6862 |
| **Day3** | 33245 | 28665.0873 | 1.1597732 | 0.25560104 | -25381.686 | 91871.6862 |
| **Day4** | 16350 | 28665.0873 | 0.57038026 | 0.57281301 | -42276.686 | 74976.6862 |
| **Day5** | 56680 | 28665.0873 | 1.97731824 | 0.05758054 | -1946.6862 | 115306.686 |
| **Day6** | 10355 | 28665.0873 | 0.36124083 | 0.72053794 | -48271.686 | 68981.6862 |
| **Day7** | 9265 | 28665.0873 | 0.32321548 | 0.74885123 | -49361.686 | 67891.6862 |
| **Day8** | 55045 | 28665.0873 | 1.92028022 | 0.06470583 | -3581.6862 | 113671.686 |
| **Day9** | 31610 | 28665.0873 | 1.10273517 | 0.27920966 | -27016.686 | 90236.6862 |
| **Day10** | 35970 | 28665.0873 | 1.25483658 | 0.21955699 | -22656.686 | 94596.6862 |
| **Day11** | 31065 | 28665.0873 | 1.0837225 | 0.28741609 | -27561.686 | 89691.6862 |
| **Day12** | -23435 | 28665.0873 | -0.817545 | 0.42028347 | -82061.686 | 35191.6862 |
| **Day13** | 49050 | 28665.0873 | 1.71114079 | 0.09773294 | -9576.6862 | 107676.686 |
| **Day14** | 35425 | 28665.0873 | 1.2358239 | 0.22644138 | -23201.686 | 94051.6862 |
| **Day15** | -2180 | 28665.0873 | -0.0760507 | 0.939901 | -60806.686 | 56446.6862 |
| **Day16** | 44690 | 28665.0873 | 1.55903938 | 0.12983468 | -13936.686 | 103316.686 |
| **Day17** | 49050 | 28665.0873 | 1.71114079 | 0.09773294 | -9576.6862 | 107676.686 |
| **Day18** | 41420 | 28665.0873 | 1.44496333 | 0.15918781 | -17206.686 | 100046.686 |
| **Day19** | -3270 | 28665.0873 | -0.1140761 | 0.90996363 | -61896.686 | 55356.6862 |
| **Day20** | 61585 | 28665.0873 | 2.14843232 | 0.04016305 | 2958.3138 | 120211.686 |
| **Day21** | 75755 | 28665.0873 | 2.64276188 | 0.01312099 | 17128.3138 | 134381.686 |
| **Day22** | -9265 | 28665.0873 | -0.3232155 | 0.74885123 | -67891.686 | 49361.6862 |
| **Day23** | -13080 | 28665.0873 | -0.4563042 | 0.65157034 | -71706.686 | 45546.6862 |
| **Day24** | 27250 | 28665.0873 | 0.95063377 | 0.34964637 | -31376.686 | 85876.6862 |
| **Day25** | -29975 | 28665.0873 | -1.0456971 | 0.30433914 | -88601.686 | 28651.6862 |
| **Day26** | -62130 | 28665.0873 | -2.167445 | 0.03855183 | -120756.69 | -3503.3138 |
| **Day27** | -45780 | 28665.0873 | -1.5970647 | 0.12109215 | -104406.69 | 12846.6862 |
| **Day28** | 30520 | 28665.0873 | 1.06470982 | 0.29579236 | -28106.686 | 89146.6862 |
| **Day29** | 83385 | 28665.0873 | 2.90893934 | 0.00689283 | 24758.3138 | 142011.686 |
| **Day30** | -2725 | 28665.0873 | -0.0950634 | 0.92491833 | -61351.686 | 55901.6862 |

*Table S18.4 Regression summary BioSA vs HEMO*

| term | estimate | std.error | statistic | p.value | conf.low | conf.high |
|---|---|---|---|---|---|---|
| **(Intercept)** | 375692.217 | 101354.25 | 3.70672386 | 0.00091712 | 168077.447 | 583306.986 |
| **HEMO** | 0.42803196 | 0.1400842 | 3.05553337 | 0.00489404 | 0.14108247 | 0.71498144 |

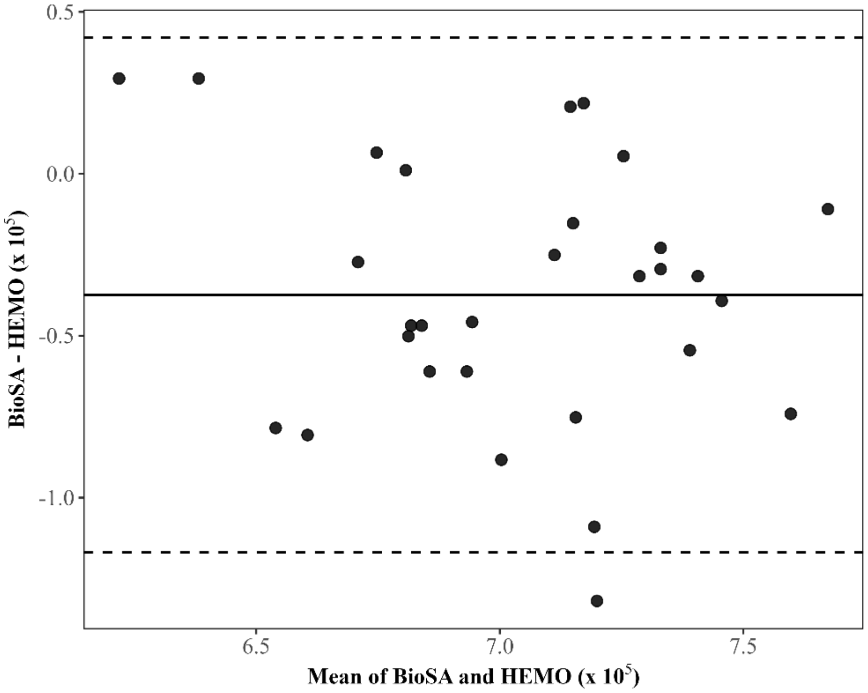


**Figure S12.** Bland–Altman analysis of agreement between BioSA and HEMO measurements. The plot shows the difference between BioSA and HEMO measurements against their mean for each paired test day. The central solid line represents the mean bias, while the dashed lines indicate the 95% limits of agreement. BioSA demonstrates a consistent measurement offset relative to HEMO, with no strong trend across the measurement range, supporting stable agreement between the two methods.

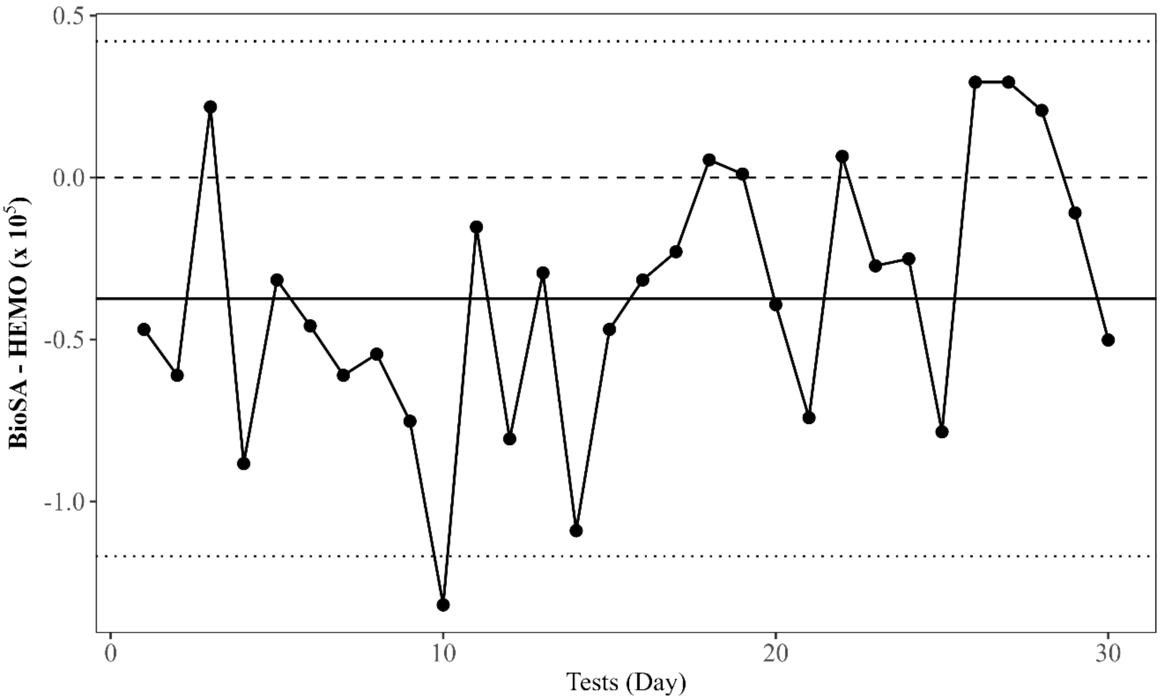


**Figure S13.** Paired differences between BioSA and HEMO across test days. Differences (BioSA − HEMO) are shown for each paired measurement across test days. The horizontal line represents the mean difference, illustrating a consistent offset between methods. The distribution of differences indicates stable agreement over time, with no systematic drift, supporting the reproducibility of BioSA relative to HEMO.

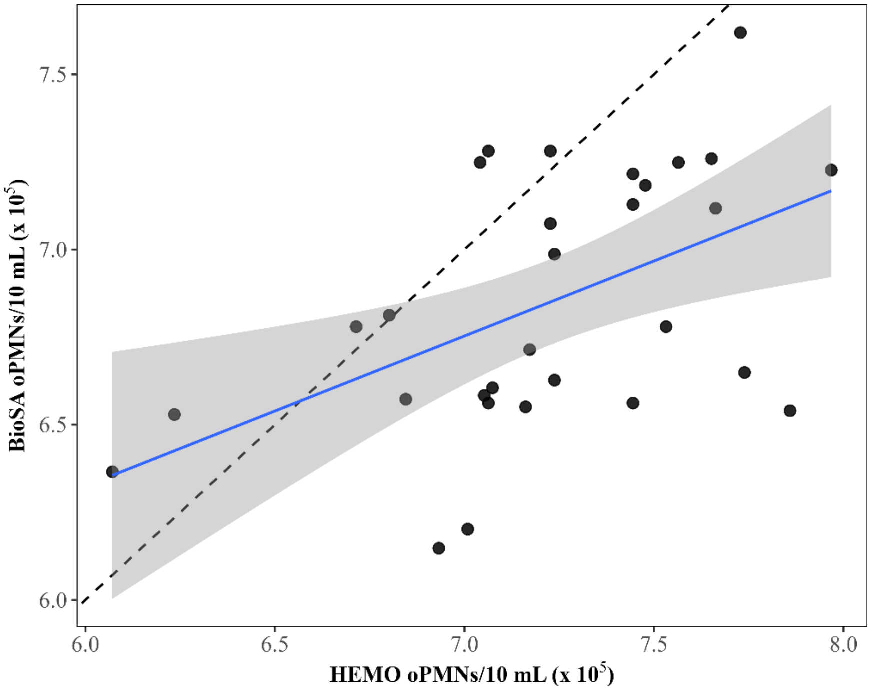


**Figure S14.** Agreement between BioSA and HEMO measurements across test days. Scatter plot comparing BioSA (DePerio) and HEMO oPMN counts for paired measurements. The fitted regression line (blue) with 95% confidence band (shaded) illustrates the relationship between the two methods, while the dashed line represents the line of identity (y = x). Although BioSA slightly underestimates HEMO values, a consistent positive association is observed, indicating reliable

agreement and reproducible measurement trends across methods. The spread of points around the identity line reflects measurement variability, while maintaining overall consistency between BioSA and the reference method.

Shapiro–Wilk tests were used to assess within-group normality for oPMN-based periodontal disease (PD) categories (Table S19). Results indicated that some groups exhibited approximate normality, while others (e.g., Gingivitis) deviated from normality at the conventional 0.05 significance level. Normality testing was not meaningful for the Severe group due to the very small sample size. Levene's test (Table S20) indicated unequal variances across groups. Given these findings, along with small and uneven group sizes, subsequent analyses were interpreted cautiously and focused on exploratory, descriptive, and data-driven approaches rather than formal parametric inference.

**Table S19.** Shapiro-Wilk Normality Test on categorization.

| ***Categories*** | ***w_value*** | ***p_value*** |
|---|---|---|
| ***Healthy*** | ***0.79528*** | ***0.05328*** |
| ***Gingivitis*** | ***0.69557*** | ***0.008602*** |
| ***Mild Periodontal*** | ***0.92565*** | ***0.5691*** |
| ***Moderate Periodontal*** | ***0.92601*** | ***0.5711*** |
| ***Severe Periodontal Disease*** | ***NA*** | ***NA*** |

**Table S20.** Levene's Test for Homogeneity of Variance.

| | | ***f_values*** | ***p_value*** |
|---|---|---|---|
| ***DF1*** | ***4*** | ***6.1187*** | ***0.003481*** |
| ***DF2*** | ***16*** | | |

Because group sizes were limited and variances differed across categories, we treated disease-stage prediction as an exploratory analysis. Data-driven clustering was used to define oPMN-

based inflammatory groupings and operational thresholds, providing an interpretable framework for assessing separability and feasibility rather than formal hypothesis testing.

## Statistical analysis methods

### *t-test*

*This test* is a statistical method used to determine if the means of two groups are significantly different from each other. It helps to assess whether the observed differences are due to actual differences in population means or just random chance. The test compares the means and calculates a p-value, which indicates the probability that the observed difference occurred by chance. A p-value less than 0.05 typically suggests that the difference is statistically significant.

### *Analysis of Variance (ANOVA)*

*ANOVA* is a statistical method used to compare the means of three or more groups by examining their variances. It breaks down the total variation in the data into variation between groups and within groups, with key components including Degrees of Freedom (DF), Sum of Squares (SS), Mean Square (MS), and the F-statistic. Degrees of Freedom measure the number of values that can vary in the analysis, divided into those for between-group variability and within-group variability. The Sum of Squares represents the total variation in the data, split into components for between-group and within-group variation. Mean Square is the average variation, calculated by dividing the Sum of Squares by the corresponding Degrees of Freedom. The F-statistic, which is the ratio of the Mean Square Between groups to the Mean Square Within groups, helps determine whether the observed differences between group means are statistically significant. ANOVA assumes that the data are normally distributed and that variances are similar across groups, and when these assumptions are met, a low p-value (less than 0.05) suggests that at least one group means is different, indicating a significant difference between the groups.

### *The Shapiro-Wilk test*

This test is a widely used statistical method for testing the normality of a dataset. In R, this test can be conveniently conducted using the shapiro.test function, which is part of the base R distribution. The function calculates the Shapiro-Wilk W statistic (w_value) and a corresponding p_value, providing a straightforward assessment of whether the data deviate significantly from a normal distribution. If the p_value is less than a chosen alpha level (commonly 0.05), it suggests that the data are not normally distributed, thereby rejecting the null hypothesis of normality. This method is particularly useful in preliminary data analysis to verify the assumption of normality before applying statistical techniques that depend on this assumption.

### *Levene's Test for Homogeneity of Variance*

*Levene's Test* is a statistical method used to determine whether multiple groups have equal variances, an important assumption for parametric tests like ANOVA. The test works by transforming the data into absolute deviations from the group means or medians and then conducting an ANOVA on these transformed values. By comparing the test statistic to a critical

value from the F-distribution, Levene's Test assesses whether the null hypothesis of equal variances can be rejected, indicating significant variance inequality among the groups.

### *The Mann-Whitney U test*

U test is a non-parametric statistical test used to assess whether there is a significant difference between the distributions of two independent groups. Unlike the t-test, this test does not require the assumption of normality, making it a robust alternative when the data do not follow a normal distribution. The Mann-Whitney U test works by ranking all observations from both groups together and comparing the sum of the ranks between the groups. A p-value less than the chosen significance level suggests rejecting the null hypothesis, indicating a significant difference between the groups. The Mann-Whitney U test is particularly useful when dealing with small sample sizes or data that contain outliers, providing a reliable method for comparison under these conditions

### *Quantization Error*

In the context of biological measurements, quantization error refers to the inaccuracy that arises when continuous biological data is approximated or measured using discrete values or categories. Biological measurements often deal with continuous variables—like concentrations of substances in blood, cell counts, or enzyme activities—that can theoretically take any value within a range. However, when these measurements are taken using instruments or methods that only allow for certain discrete steps or intervals, a quantization error is introduced. To make the quantization error more interpretable, it is often expressed as a percentage of the mean of the measured values. For this work, the standard deviation of the residuals is divided by the mean of the measured values. This ratio is then multiplied by 100 to convert it into a percentage.

### *The k-means clustering algorithm*

*k-means* is a method used to partition a dataset into k clusters, each represented by a centroid. The process starts with choosing the number of clusters, *k*, and initializing the centroids either randomly or by calculating the mean of subsets of the data. Each data point is then assigned to the nearest cluster based on the Euclidean distance to the centroid. The centroids are updated by calculating the mean of the points in each cluster, and this process is repeated until the centroids stabilize. The algorithm minimizes the Within-Cluster Sum of Squares (WCSS), measuring the variance within clusters to evaluate the clustering quality. k-means is effective for grouping data, especially when categories are not predefined or when there's overlap in data characteristics.

### *Longitudinal data analysis*

This method examines data collected from the same subject multiple times over a period. Fixed-effects models are particularly suited for this analysis as they focus on changes within each subject rather than comparing different subjects to each other. This approach helps control individual differences that are not directly measured but may impact the results. In simpler terms, fixed-effects models isolate variations within each subject over time. This means that with repeated measurements from the same person in the same group, the model uses everyone's data to understand how changes over time affect the outcome, while disregarding differences between different individuals. This approach is beneficial because it allows us to

discern the true effect of time and other time-varying factors within the same person, without being confounded by inherent differences between individuals. In a longitudinal study focusing on a single subject, the fixed-effects model investigates how time-varying predictors influence the outcome variable for that subject over time. Here is the mathematical formulation for a single subject $Y_t = \beta_0 + \beta_1 X_{t1} + \beta_2 X_{t2} + \cdots + \beta_p X_{tp} + \epsilon_t$ Where $\beta_0$ is the intercept term. Also $\beta_1, \beta_2, \ldots$ and $\beta_p$ are the coefficients for the time-varying predictors $X_{t1}, X_{t2}, \ldots X_{tp}$ . $\epsilon_t$ is the error term at time t where the measurements were performed in 30 differed groups or times, assumed to be independently and identically distributed with mean zero and constant variance.

# Further CS characterization results

Cornstarch (CS) was selected as the hydrophilic coating material due to its hydroxyl-rich polysaccharide structure, which confers strong water affinity and promotes cell adhesion; supporting references have been added. In this study, the CS layer was prepared by depositing the CS solution onto the dish surface, allowing it to settle and partially air-dry before a gentle rinse. This process produces a gel-like coating rather than a sharply defined dry thin film. Because the coating is applied manually, the resulting layer thickness is governed primarily by solution concentration rather than precise deposition control; however, the functional performance of the coating—namely hydrophilicity, transparency, and adhesion efficiency—was consistently achieved across CS concentrations up to 1%. Literature reports [6,7] indicate that starch-based coatings formed under similar casting conditions typically yield nanoscale surface layers, which aligns with the physical behavior of our coating. The CS-coated surface exhibited a contact angle of <25°, confirming strong hydrophilicity. The concentration-dependent enhancement of oPMN attachment is quantified in Figure 3 and further supported by tables and supplementary figures. Transparency of the coating is demonstrated in Figure S9_A(i)–S9_A(iii) and S9_B(i)–B(iv) (Supporting Information). To evaluate stability, CS-coated dishes were exposed to PBS for up to 20 minutes, during which more than 95% of the coating remained intact with no visible dissolution, peeling, or delamination, confirming that the hydrated CS matrix maintains structural integrity under wet experimental conditions. The coated surfaces appeared macroscopically uniform across repeated preparations, without cracks or patchiness, and remained sufficiently transparent for imaging. As the adhesion behavior is governed primarily by the hydrophilic polymeric network rather than a specific thickness value, precise dry-film thickness was not measured. To further illustrate the adhesion mechanism, Figure 9S_C(i)-9S_C(ii) shows how cornstarch binds both to the glass substrate and to hydrophilic residues on CD11b. The hydroxyl groups on glucose form hydrogen bonds with silanol groups on glass, creating a stable hydrated coating, while exposed polar residues on CD11b (such as serine, threonine, or aspartate) can form similar hydrogen bonds with the cornstarch matrix. These weak, reversible interactions support the preferential attachment of oPMNs to the CS-coated surface.

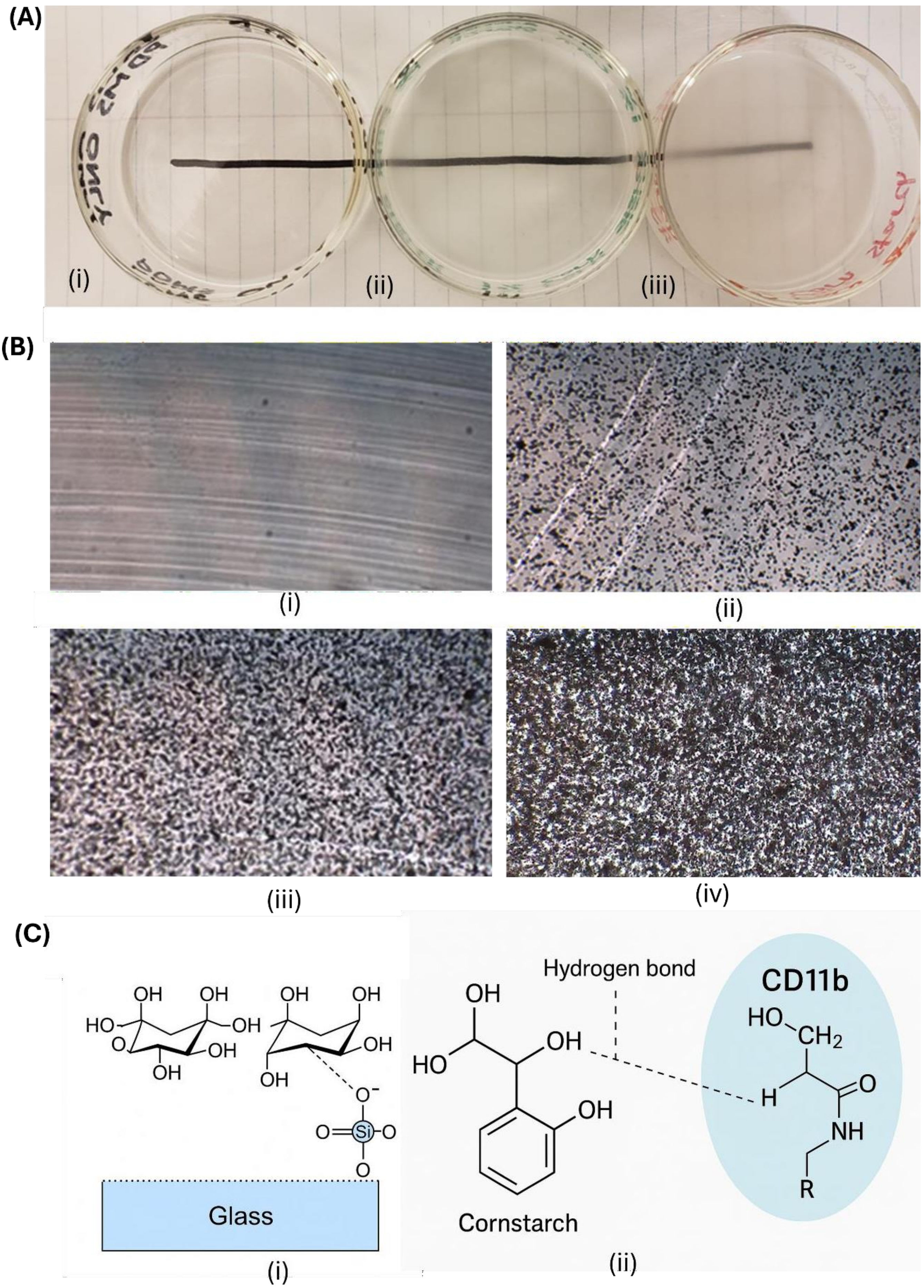
(A)
(i)
(ii)
(iii)
(B)
(i)
(ii)
(iii)
(iv)
(C)
OH
HO
O
OH
Glass
(i)
Hydrogen bond
CD11b
HO
CH2
H
O
NH
R
Cornstarch
(ii)

**Figure S15.** (A) Cornstarch–PDMS samples prepared in separate Petri dishes containing 0% (left), 1% (middle), and 5% (right) (w/w) cornstarch. Each sample is positioned in front of a drawn reference line on ruled paper to illustrate transparency differences. (B) Microscopy images of cornstarch–PDMS samples containing (i) 0% cornstarch, (ii) 0.1% (w/w) cornstarch, (iii) 1% (w/w) cornstarch, and (iv) 5% (w/w) cornstarch. (C) Interaction mechanisms between cornstarch, glass, and CD11b. *(i)* Schematic representation of how cornstarch (glucose units with multiple –OH groups) forms hydrogen bonds with surface silanol (Si–OH) groups on glass, producing a stable, hydrated polysaccharide layer. *(ii)* Illustration of how exposed polar amino acid residues on CD11b—shown here as a representative serine side chain—can form hydrogen bonds with hydroxyl groups on cornstarch. These reversible, non-covalent interactions explain how hydrophilic regions of oral neutrophils adhere to the CS-coated surface.

# References


1 Lin, T. Y. *et al.* in *2017 IEEE Conference on Computer Vision and Pattern Recognition (CVPR).* 936-944.

2 Redmon, J. & Farhadi, A. in *2017 IEEE Conference on Computer Vision and Pattern Recognition (CVPR).* 6517-6525.

3 *LabelImg. Git code*, <https://github.com/tzutalin/labelImg> (2015).

4 Lin, T.-Y. *et al.* in *European conference on computer vision.* 740-755 (Springer).

5 Hassani, M. *et al.* On the origin of low-density neutrophils. *Journal of leukocyte biology* **107**, 809-818 (2020).

6 Żołek-Tryznowska, Z. & Kałuża, A. The influence of starch origin on the properties of starch films: packaging performance. *Materials* **14**, 1146 (2021).

7 Matheus, J. R. V. *et al.* Biopolymers as green-based food packaging materials: a focus on modified and unmodified starch-based films. *Comprehensive Reviews in Food Science and Food Safety* **22**, 1148-1183 (2023).
